# Quinazolin-4(3*H*)-ones and 5,6-Dihydropyrimidin-4(3*H*)-ones from β-Aminoamides and Orthoesters

**DOI:** 10.3390/molecules23112925

**Published:** 2018-11-09

**Authors:** Joshua T. Gavin, Joel K. Annor-Gyamfi, Richard A. Bunce

**Affiliations:** Department of Chemistry, Oklahoma State University, Stillwater, OK 74078-3071, USA; jtgavin@csbsju.edu (J.T.G.); jannorg@okstate.edu (J.K.A.-G.)

**Keywords:** quinazolin-4(3*H*)-ones, 5,6-dihydropyrimidin-4(3*H*)-ones, heterocycle synthesis, 2-aminobenzamides, orthoesters

## Abstract

Quinazolin-4(3*H*)-ones have been prepared in one step from 2-aminobenzamides and orthoesters in the presence of acetic acid. Simple 2-aminobenzamides were easily converted to the heterocycles by refluxing in absolute ethanol with 1.5 equivalents of the orthoester and 2 equivalents of acetic acid for 12–24 h. Ring-substituted and hindered 2-aminobenzamides as well as cases incorporating an additional basic nitrogen required pressure tube conditions with 3 equivalents each of the orthoester and acetic acid in ethanol at 110 °C for 12–72 h. The reaction was tolerant towards functionality on the benzamide and a range of structures was accessible. Workup involved removal of the solvent under vacuum and either recrystallization from ethanol or trituration with ether-pentane. Several 5,6-dihydropyrimidin-4(3*H*)-ones were also prepared from 3-amino-2,2-dimethylpropionamide. All products were characterized by melting point, FT-IR, ^1^H-NMR, ^13^C-NMR, and HRMS.

## 1. Introduction

Quinazolin-4(3*H*)-ones are valuable scaffolds in pharmaceutical chemistry due to their diverse biological activities. Drugs possessing the quinazolinone unit are used as anti-anxiety, antibacterial anticonvulsant, antifungal, anti-inflammatory, and antitumor agents [1]. Some examples of 2-alkyl- and 2-arylquinazolin-4(3*H*)-ones currently on the market include the sedative-hypnotics methaqualone (quaalude, **1**) [2] and afloqualone (**2**) [3,4] as well as the antifungal albaconazole (**3**) [5]. Additionally, compound **4** [6] is currently under evaluation as an antibacterial agent against both Gram-positive and Gram-negative organisms.

**Figure 1:**
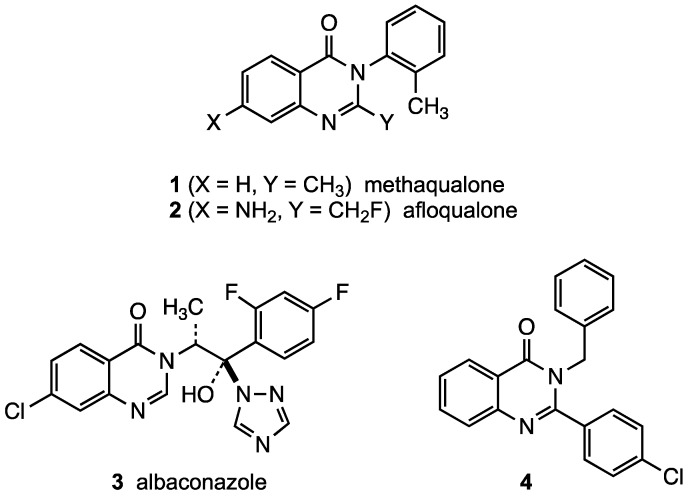
Bioactive quinazolin-4(3*H*)-ones.

In this study, we report the synthesis of 2-alkyl- and 2-arylquinazolin-4(3*H*)-ones from inexpensive 2-aminobenzamide and orthoester substrates without the need for metal catalysts or Lewis acids. The process is similar to earlier acid-catalyzed processes reported by our group for the preparation of 2-substituted 2,3-dihydroquinazolin-4(1*H*)-ones [7] and benzimidazoles [8] as well as for a synthesis of benzimidazo[1,2-*c*]quinazolines described by others [9]. Using the conditions disclosed herein, a small library of substituted quinazolin-4(3*H*)-ones has been generated with high yields (Figure 1). We also report the synthesis of a modest selection of 5,6-dihydropyrimidin-4(3*H*)-ones from 3-amino-2,2-dimethyl-propionamide under identical conditions.

To date, orthoesters have received minimal use for the preparation of these systems. Syntheses that did employ these building blocks focused almost exclusively on the use of triethyl orthoformate to prepare derivatives without C2 substitution [5,10]. Although the current method is somewhat limited by the sparse number of available orthoesters, the simplicity of our approach and its ability to provide a wide range of targets makes it a valuable addition to the field.

The current reaction is a variant of the classical Niementowski quinazoline synthesis, which involved the cyclocondensation of an *N*-alkylamide with anthranilic acid [11]. The original conditions for this transformation, however, proved somewhat limiting as they required extended heating at >200 °C and resulted in significant decarboxylation of the acid. Further work showed that methyl anthranilate was more stable at 200 °C and afforded higher yields with less degradation of the substrate [12]. The decarboxylation problem was also minimized through the application of microwave conditions, which considerably reduced the reaction time [13,14,15], and the use of thioamides, which permitted lower reaction temperatures [16]. Later efforts found that isatoic anhydride with amines and orthoesters under neat conditions gave clean cyclization to the quinazolinones at temperatures of 120 °C [17]. In a related procedure, heating isatoic anhydride with benzylamines at 120 °C in ionic liquids led to oxidation of the benzylic amine to the imine, followed by cyclization [18]. More recently, phosphoric acid was employed to promote the synthesis of 2,3-disubstituted quinazolin-4(3*H*)-ones from 2-amino-*N*- substituted benzamides and β-ketoesters [19]. Likewise, *n*-propanephosphonic anhydride was used as a condensing agent to assemble 2-fluoroalkyl-substituted derivatives from fluoroalkanoic acids and anthranilic acid [20]. Along a different line, two separate reports detailed the synthesis of quinazolinones by oxidative cyclization of aldehydes with 2-aminobenzamide under aerobic conditions [6] and in the presence of *tert*-butyl hydroperoxide (TBHP)-potassium iodide [21]. A similar oxidative condensation of primary alcohols with 2-aminobenzamide to generate quinazolinones was promoted by TBHP alone [22] and together with iodine and dimethyl sulfoxide [23,24]. Numerous metals such as manganese [25], iron [26], nickel [27], zinc [28], ruthenium [29], iridium [30], and platinum [31] in conjunction with TBHP were also reported to promote this reaction. The option of using primary alcohols for this transformation dramatically increased the scope of the synthesis due to the vast array of available substrates. Finally, other methods involved the reaction of lithium 2-(diethylaminocarbonyl)anilide with aryl- and alkylnitriles [32] as well as various copper [33,34,35,36,37,38,39,40] and palladium [2,41,42,43,44] catalyzed heterocyclizations.

## 2. Results and Discussion

The results of our study are summarized in Table 1. The products listed illustrate the breadth of systems accessible using our procedure. Several acid catalysts were evaluated, but the most convenient and inexpensive was acetic acid. For 2-aminobenzamide (**5**), the reaction required heating 1 equivalent of the amide with 1.5–2 equivalents of the orthoester and 2 equivalents of acetic acid in absolute ethanol at reflux (78 °C) for 12–24 h (Method 1). Less reactive ring-substituted derivatives (**7**, **9**, and **11**), more hindered 2-amino-*N*-substituted benzamides (**15** and **17**) and substrates incorporating a second basic nitrogen (**13** and **19**) required pressure tube conditions with 3 equivalents of the orthoester and 3 equivalents of acetic acid in ethanol at 110 °C for 12–72 h (Method 2). Comparisons of Methods 1 and 2 are shown in the Table for several substrates and clearly demonstrate the advantage of using higher temperatures. Beyond these few examples, however, additional comparisons were not possible as pure material was often difficult to isolate from reactions run at 78 °C. Two examples that did permit comparison were the chloro-substituted 2-aminobenzamides **9** and **11** with triethyl orthobenzoate. Using Method 1, this reaction gave low conversions, and except for a small amount of product that initially crystallized from the cooling reaction mixture, the quinazolinones were contaminated with unreacted starting material and inseparable by-products. Increasing the stoichiometry of the orthoester under these conditions failed to improve the conversion to a satisfactory level. Alternatively, increasing the amount of acid to 6 equivalents, improved the yields of **10d** and **12d** to 56% and 77%, respectively, but repeated crystallizations were required for purification. Reacting these substrates according to Method 2, with 2–3 equivalents of the orthoester and 3 equivalents of acetic acid, however, gave nearly quantitative conversions to the products. Under this protocol, trituration of the crude products with 5% ether in pentane was sufficient to obtain spectroscopically pure material. Clearly, Method 2 proved superior for the broadest range of substrates. All reported yields were isolated, but not all were fully optimized. Procedures and copies of the ^1^H and ^13^C-NMR spectra are given in the Supplemental Material.

The yields using Method 2 were generally high for all of the substrates evaluated. While reactions were followed by thin layer chromatography, attempts at preparative purifications using silica gel often led to decomposition of the products, even when the eluting solvent contained 1% triethylamine. Sublimation also afforded limited success. Thus, product purification was restricted to crystallization from ethanol or trituration with 5% ether in hexanes. Fortunately, all of the derivatives prepared were highly crystalline solids, which facilitated purification using these techniques.

**Scheme 1:**
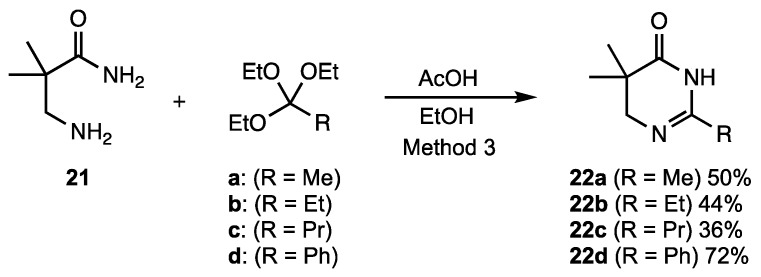
Synthesis 5,6-dihydropyrimidin-4(3*H*)-ones.

Finally, we were able to extend the cyclization process to the preparation of a small family of 5,6-dihydropyrimidin-4(3*H*)-ones from 3-amino-2,2-dimethylpropionamide (**21**) (see Scheme 1). These compounds were prepared by Method 3 using 1.5 equivalents of the orthoester and 2 equivalents of acetic acid in ethanol at reflux for 24 h. Cyclizations to produce **22a**–**d** were assisted by the Thorpe–Ingold (*gem*-dialkyl) effect [45,46], but the yields were modest due to difficulties in crystallizing the final products. Since the pyrimidinones were water-soluble and hygroscopic, it was necessary to utilize dry solvents for the crystallization. Best results were achieved by adding dry chloroform to the crude product and allowing the mixture to sit under nitrogen for four to seven days. The use of higher temperatures (Method 2) for these substrates had little effect on the yields.

There are a number of very positive features in this synthesis. The reaction was free of metals and corrosive Lewis acids, requiring only a 2–3-fold excess of acetic acid to promote the reaction. This simplified the purification of the final products and eliminated the possibility of residual contaminants. Both sets of conditions were tolerant of alkyl, halide and basic functional groups present on the 2-aminobenzamide reacting partner and a small library of substituted derivatives was prepared. Employing Method 2, it was possible to obtain high yields of quinazolinones from ring-substituted 2-aminobenzamides **7**, **9**, and **11**. This procedure also promoted excellent conversions of relatively hindered 2-amino-*N*-methyl- and 2-amino-*N*-phenylbenzamides **15** and **17**, respectively, to 2,3-disubstituted quinazolinones **16** and **18**. These hindered ring closures were likely facilitated by the rigid aromatic scaffold, which held the ortho-disposed reactive centers in close proximity. Finally, **13** and **19,** which both possessed basic nitrogen groups, gave superb yields as well. In ring closures of hydrazide **19**, it was noted that the less reactive amide nitrogen cyclized in preference to the more nucleophilic terminal nitrogen of the hydrazide to give exclusively the six-membered cyclic product, with none of the seven-membered ring observed. The preparation of the 5,6-dihydropyrimidin-4(3*H*)-ones was more limited due to challenges during the purification process. Additional experiments attempted to cyclize 3-aminopropionamide, which was commercially available as the hydrochloride salt. This substrate, however, lacked the *gem*-dimethyl moiety and did not undergo significant ring closure.

**Figure 2:**
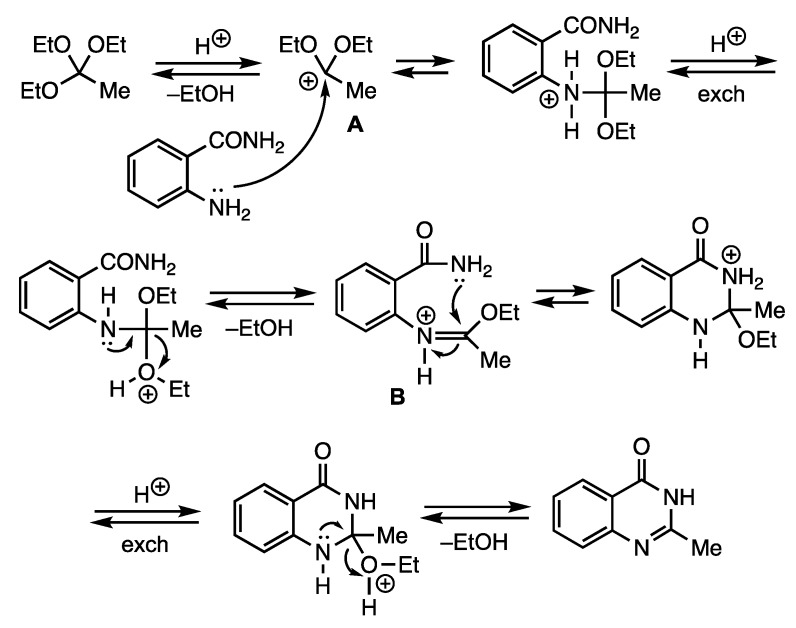
Mechanism for quinazolin-4(3*H*)-one formation.

A plausible mechanism for quinazolinone formation is depicted for the reaction of 2-aminobenzamide with triethyl orthoacetate (see Figure 2). The process involves initial protonation of the orthoester and loss of ethanol to afford the stabilized carbocation **A**. Subsequent attack on **A** by the aniline amino group, proton exchange, and loss of a second molecule of ethanol would then yield the iminium ion **B**. Finally, closure of the amide nitrogen on the iminium carbon, proton exchange, and loss of ethanol would generate the quinazolinone product. A similar mechanism is likely operating for the closure of the 5,6-dihydropyrimidin-4(3*H*)-ones.

## 3. Experimental Section

### 3.1. General Methods

Most commercial chemicals and solvents were used as received, except for 2-amino-4-methylbenzamide, which required recrystallization from ethanol. 2-Amino-*N*-phenylbenzamide was prepared from 2-nitrobenzoic acid, according to the following sequence: (*i*) SOCl_2_ (2.0 equiv), benzene, reflux, 2 h; (*ii*) PhNH_2_ (1.1 equiv), CH_2_Cl_2_, Et_3_N (2.0 equiv), 0 °C to r.t., 4 h; (*iii*) Fe (3.2 equiv)/NH_4_Cl (1.0 equiv), aq EtOH, 85 °C, 2 h [47]; 82% overall, white solid, m.p. 123–125 °C (lit [48] m.p. 128–129.5 °C). The spectral data matched those reported [48].

Unless otherwise indicated, all reactions were carried out under dry N_2_ in oven-dried glassware. Reactions were monitored by thin layer chromatography on silica gel GF plates (Analtech no. 21521, Newark, DE, USA). Band elution was monitored using a hand-held UV lamp (Fisher Scientific, Pittsburgh, PA, USA). Melting points were obtained using a MEL-TEMP apparatus (Cambridge, MA, USA) and are uncorrected. FT-IR spectra were run using a Varian Scimitar FTS 800 spectrophotometer (Randolph, MA, USA) as thin films or nujol mulls on NaCl disks. ^1^H- and ^13^C-NMR spectra were measured using a Bruker Avance 400 system (Billerica, MA, USA) in the indicated solvents at 400 MHz and 101 MHz, respectively, with (CH_3_)_4_Si as the internal standard; coupling constants (*J*) are given in Hz. High-resolution mass spectra (HRMS-ESI) were obtained using a Thermo LTQ-Orbitrap XL mass spectrometer (Thermo Scientific, Waltham, MA, USA).

### 3.2. General Procedures to Prepare Quinazolin-4(3H)-ones

#### 3.2.1. Method 1

The orthoester (1.5 equiv) was added to a mixture of the 2-aminobenzamide (1.0 equiv) in absolute ethanol (3 mL). Glacial acetic acid (2 equiv) was added and the reaction was heated at reflux for 12–24 h. The reaction mixture was cooled and concentrated under vacuum. If the crude product was pure by ^1^H-NMR, it was triturated with 5% ether in pentane. If it was not pure, it was recrystallized from ethanol. In some cases, it was necessary to remove excess orthoester under high vacuum at 50 °C prior to purification. The following compounds were prepared:

2-Methylquinazolin-4(3*H*)-one (**6a**). This compound was prepared from 2-aminobenzamide (125 mg, 0.92 mmol), triethyl orthoacetate (224 mg, 253 µL, 1.38 mmol) and acetic acid (110 mg, 105 µL, 1.84 mmol) in refluxing absolute ethanol (3 mL). Yield: 135 mg (92%) as an off-white solid, m.p. 236–238 °C (lit [40] m.p. 242–244 °C); IR (CHCl_3_): 3176, 1674 cm^−1^; ^1^H-NMR (400 MHz, CDCl_3_): δ 11.2 (br s, 1H), 8.28 (dd, *J* = 8.0, 1.0 Hz, 1H), 7.78 (td, *J* = 8.1, 1.5 Hz, 1H), 7.68 (d, *J* = 8.1 Hz, 1H), 7.48 (td, *J* = 8.0, 1.0 Hz, 1H), 2.58 (s, 3H); ^13^C-NMR (101 MHz, CDCl_3_): δ 163.9, 153.1, 149.6, 135.1, 127.2, 126.7, 126.4, 120.5, 22.4; HRMS (ESI): *m/z* for C_9_H_8_N_2_O [M + 1]^+^: calcd.: 161.0715; found: 161.0714.

2-Ethylquinazolin-4(3*H*)-one (**6b**). This compound was prepared from 2-aminobenzamide (125 mg, 0.92 mmol), triethyl orthopropionate (244 mg, 278 µL, 1.38 mmol) and acetic acid (110 mg, 105 µL, 1.84 mmol) in refluxing absolute ethanol (3 mL). Yield: 155 mg (89%) as an off-white solid, m.p. 235–237 °C (lit [15] m.p. 238–240 °C); IR (CHCl_3_): 3169, 1681 cm^−1^; ^1^H-NMR (400 MHz, CDCl_3_): δ 11.2 (br s, 1H), 8.29 (dd, *J* = 8.0, 1.2 Hz, 1H), 7.78 (ddd, *J* = 8.3, 7.0, 1.6 Hz, 1H), 7.71 (d, *J* = 8.1 Hz, 1H), 7.47 (ddd, *J* = 8.1, 7.1, 1.2 Hz, 1H), 2.82 (q, *J* = 7.3 Hz, 2H), 1.45 (t, *J* = 7.4 Hz, 3H); ^13^C-NMR (101 MHz, CDCl_3_): δ 164.0, 157.5, 149.5, 134.9, 127.4, 126.6, 126.4, 120.8, 29.3, 11.6; HRMS (ESI): *m/z* for C_10_H_10_N_2_O [M + 1]^+^: calcd.: 175.0871; found: 175.0873.

2-Propylquinazolin-4(3*H*)-one (**6c**). This compound was prepared from 2-aminobenzamide (125 mg, 0.92 mmol), triethyl orthobutyrate (262 mg, 298 µL, 1.38 mmol) and acetic acid (110 mg, 105 µL, 1.84 mmol) in refluxing absolute ethanol (3 mL). Yield: 160 mg (93%) as an off-white solid, m.p. 212–214 °C (lit [15] m.p. 210–212 °C); IR (CHCl_3_): 3165, 1675 cm^−1^; ^1^H-NMR (400 MHz, CDCl_3_): δ 11.2 (br s, 1H), 8.29 (d, *J* = 7.8 Hz, 1H), 7.77 (t, *J* = 7.8 Hz, 1H), 7.70 (d, *J* = 8.0 Hz, 1H), 7.47 (t, *J* = 7.8 Hz, 1H), 2.76 (t, *J* = 7.4 Hz, 2H), 1.92 (sextet, *J* = 7.4 Hz, 2H), 1.08 (t, *J* = 7.3 Hz, 3H) ; ^13^C-NMR (101 MHz, CDCl_3_): δ 169.3, 156.6, 149.5, 134.9, 127.4, 126.6, 126.4, 120.7, 38.0, 21.1, 13.9; HRMS (ESI): *m/z* for C_11_H_12_N_2_O [M + 1]^+^: calcd.: 189.1028; found: 189.1025.

2-Phenylquinazolin-4(3*H*)-one (**6d**). This compound was prepared from 2-aminobenzamide (125 mg, 0.92 mmol), triethyl orthobenzoate (309 mg, 312 µL, 1.38 mmol) and acetic acid (110 mg, 105 µL, 1.84 mmol) in refluxing absolute ethanol (3 mL). Yield: 134 mg (81%) as an off-white solid, m.p. 234–236 °C (lit [40] m.p. 233–235 °C); IR (CHCl_3_): 3196, 1668 cm^−1^; ^1^H-NMR (400 MHz, CDCl_3_): δ 11.1 (br s, 1H), 8.33 (d, *J* = 8.0 Hz, 1H), 8.21 (m, 2H), 7.86–7.78 (complex, 2H), 7.61–7.57 (complex, 3H), 7.52 (ddd, *J* = 7.9, 6.8, 1.2 Hz, 1H); ^13^C-NMR (101 MHz, CDCl_3_): δ 169.9, 151.5, 149.4, 134.9, 132.8, 131.7, 129.2, 128.1, 127.1, 126.9, 126.5, 121.0; HRMS (ESI): *m/z* for C_14_H_10_N_2_O [M + 1]^+^: calcd.: 223.0871; found: 223.0870.

Quinazolin-4(3*H*)-one (**6e**). This compound was prepared from 2-aminobenzamide (125 mg, 0.92 mmol), triethyl orthoformate (204 mg, 229 µL, 1.38 mmol) and acetic acid (110 mg, 105 µL, 1.84 mmol) in refluxing absolute ethanol (3 mL). Yield: 117 mg (87%) as an off-white solid, m.p. 212–214 °C (lit [16] m.p. 215.5–216.5 °C); IR (CHCl_3_): 3202, 1664 cm^−1^; ^1^H-NMR (400 MHz, CDCl_3_): δ 8.31 (dd, *J* = 8.0, 1.0 Hz, 1H), 8.08 (s, 1H), 7.28 (td, *J* = 8.1, 1.4 Hz, 1H), 7.77 (dd, *J* = 8.1, 1.0 Hz, 1H), 7.55 (td, *J* = 7.4, 1.4 Hz, 1H), NH not observed; ^13^C-NMR (101 MHz, CDCl_3_): δ 162.8, 149.0, 143.5, 135.1, 128.0, 127.6, 126.6, 122.7; HRMS (ESI): *m/z* for C_8_H_6_N_2_O [M + 1]^+^: calcd.: 147.0558; found: 147.0557.

#### 3.2.2. Method 2

The 2-aminobenzamide (1 equiv), the orthoester (2–3 equiv) and absolute ethanol (2–3 mL) were placed in a 15-mL Chemglass screw-cap pressure tube (No. CG-1880-01, Chemglass, Vineland, NJ, USA). Glacial acetic acid (3 equiv) was added, N_2_ was introduced to the vessel and the cap was tightened. The vessel was heated at 110 °C for 12–72 h, then cooled and concentrated to give the quinazolinone, which was purified by crystallization from absolute ethanol or trituration from 5% ether in pentane. The following compounds were prepared:

2,7-Dimethylquinazolin-4(3*H*)-one (**8a**). This compound was prepared from 2-amino-4-methylbenzamide (100 mg, 0.67 mmol), triethyl orthoacetate (217 mg, 245 µL, 1.34 mmol) and acetic acid (121 mg, 115 µL, 2.01 mmol) in absolute ethanol (2 mL) at 110 °C for 24 h to give 105 mg (90%) as a white solid, m.p. 260–262 °C (lit [49] m.p. 263–264 °C); IR (CHCl_3_): 3169, 1677 cm^−1^; ^1^H-NMR (400 MHz, CDCl_3_): δ 11.6 (br s, 1H), 8.16 (d, *J* = 8.1 Hz, 1H), 7.47 (br s, 1H), 7.29 (dd, *J* = 8.1, 1.0 Hz, 1H), 2.57 (s, 3H), 2.51 (s, 3H); ^13^C-NMR (101 MHz, CDCl_3_): δ 163.9, 153.2, 149.5, 146.0, 128.0, 126.8, 126.1, 117.9, 22.1, 22.0; HRMS (ESI): *m/z* for C_10_H_10_N_2_O [M + 1]^+^: calcd.: 175.0871; found: 175.0867.

2-Ethyl-7-methylquinazolin-4(3*H*)-one (**8b**). This compound was prepared from 2-amino-4-methylbenzamide (100 mg, 0.67 mmol), triethyl orthopropionate (236 mg, 269 µL, 1.34 mmol) and acetic acid (121 mg, 115 µL, 2.01 mmol) in absolute ethanol (2 mL) at 110 °C for 24 h. Yield: 114 mg (91%) as a white solid, m.p. 244–245 °C; IR (CHCl_3_): 3178, 1674 cm^−1^; ^1^H-NMR (400 MHz, CDCl_3_): δ 11.2 (br s, 1H), 8.17 (d, *J* = 8.1 Hz, 1H), 7.50 (br s, 1H), 7.30 (dd, *J* = 8.1, 0.9 Hz, 1H), 2.80 (q, *J* = 7.6 Hz, 2H), 2.51 (s, 3H), 1.43 (t, *J* = 7.6 Hz, 3H); ^13^C-NMR (101 MHz, CDCl_3_): δ 163.8, 157.4, 149.5, 145.8, 128.0, 127.0, 126.1, 118.2, 29.2, 22.0, 11.5; HRMS (ESI): *m/z* for C_11_H_12_N_2_O [M + 1]^+^: calcd.: 189.1028; found: 189.1031.

7-Methyl-2-phenylquinazolin-4(3*H*)-one (**8d**). This compound was prepared from 2-amino-4-methylbenzamide (100 mg, 0.67 mmol), triethyl orthobenzoate (300 mg, 303 µL, 1.34 mmol) and acetic acid (121 mg, 115 µL, 2.01 mmol) in absolute ethanol (2 mL) at 110 °C for 48 h. Yield: 133 mg (84%) as a light yellow solid, m.p. 239–241 °C (lit [40] m.p. 240–241 °C); IR (CHCl_3_): 3156, 1672 cm^−1^; ^1^H-NMR (400 MHz, CDCl_3_): δ 10.9 (br s, 1H), 8.21 (d, *J* = 8.1 Hz, 1H), 8.18 (m, 2H), 7.64 (br s, 1H), 7.61–7.56 (complex, 3H), 7.33 (dd, *J* = 8.2, 0.9 Hz, 1H), 2.54 (s, 3H); ^13^C-NMR (101 MHz, CDCl_3_): δ 163.3, 151.6, 149.6, 146.0, 132.9, 131.6, 1291, 128.5, 127.8, 127.2, 126.2, 118.5, 22.0; HRMS (ESI): *m/z* for C_15_H_12_N_2_O [M + 1]^+^: calcd.: 237.1028; found: 237.1023.

6-Chloro-2-methylquinazolin-4(3*H*)-one (**10a**). This compound was prepared from 2-amino-5-chlorobenzamide (100 mg, 0.59 mmol) triethyl orthoacetate (191 mg, 216 µL, 1.18 mmol) and acetic acid (106 mg, 101 µL, 1.77 mmol) in absolute ethanol (3 mL) at 110 °C for 24 h. Yield: 106 mg (92%) as a light tan powder, m.p. 288–290 °C (lit [49] m.p. 289–291 °C); IR (CHCl_3_): 3166, 1693, 1631 cm^−1^; ^1^H-NMR (400 MHz, CDCl_3_): δ 12.4 (br s, 1H), 8.00 (d, *J* = 2.5 Hz, 1H), 7.79 (dd, *J* = 8.7, 2.5 Hz, 1H), 7.59 (d, *J* = 8.7 Hz, 1H), 2.35 (s, 3H); ^13^C-NMR (101 MHz, DMSO-*d_6_*): δ 160.7, 154.9, 147.7, 134.4, 130.1, 128.9, 124.7, 121.9, 21.5; HRMS (ESI): *m/z* for C_9_H_7_^35^ClN_2_O [M + 1]^+^: calcd.: 195.0325; found: 195.0323.

6-Chloro-2-ethylquinazolin-4(3*H*)-one (**10b**). This compound was prepared from 2-amino-5-chlorobenzamide (100 mg, 0.59 mmol), triethyl orthopropionate (208 mg, 237 µL, 1.18 mmol) and acetic acid (106 mg, 101 µL, 1.77 mmol) in absolute ethanol (3 mL) at 110 °C for 24 h. Yield: 114 mg (92%) as a light tan powder, m.p. 262–264 °C; IR (nujol): 3169, 1681, 1627 cm^−1^; ^1^H-NMR (400 MHz, DMSO-*d_6_*): δ 12.3 (br s, 1H), 8.01 (d, *J* = 2.5 Hz, 1H), 7.79 (dd, *J* = 8.7, 2.5 Hz, 1H), 7.62 (d, *J* = 8.7 Hz, 1H), 2.62 (q, *J* = 7.5 Hz, 2H), 1.24 (t, *J* = 7.5 Hz, 3H); ^13^C-NMR (101 MHz, DMSO-*d_6_*): δ 160.8, 159.0, 147.7, 134.3, 130.1, 129.1, 124.7, 122.1, 27.8, 11.2; HRMS (ESI): *m/z* for C_10_H_9_^35^ClN_2_O [M + 1]^+^: calcd.: 209.0482; found: 209.0484.

6-Chloro-2-propylquinazolin-4(3*H*)-one (**10c**). This compound was prepared from 2-amino-5-chlorobenzamide (100 mg, 0.59 mmol), triethyl orthobutyrate (224 mg, 255 µL, 1.18 mmol) and acetic acid (106 mg, 101 µL, 1.77 mmol) in absolute ethanol (3 mL) at 110 °C for 24 h. Yield: 116 mg (88%) as a light tan powder, m.p. 252–254 °C (lit [33] m.p. 257–259 °C); IR (nujol): 3165, 1681, 1618 cm^−1^; ^1^H-NMR (400 MHz, CDCl_3_): δ 12.4 (br s, 1H), 8.01 (d, *J* = 2.4 Hz, 1H), 7.80 (dd, *J* = 8.7, 2.4 Hz, 1H), 7.62 (d, *J* = 8.7 Hz, 1H), 2.57 (t, *J* = 7.4 Hz, 2H), 1.74 (sextet, *J* = 7.4 Hz, 2H), 0.93 (t, *J* = 7.4 Hz, 3H); ^13^C-NMR (101 MHz, DMSO-*d_6_*): δ 160.8, 158.0, 147.7, 134.4, 130.1, 129.1, 124.7, 122.1, 36.3, 20.1, 13.5; HRMS (ESI): *m/z* for C_11_H_11_^35^ClN_2_O [M + 1]^+^: calcd.: 223.0638; found: 223.0637.

6-Chloro-2-phenylquinazolin-4(3*H*)-one (**10d**). This compound was prepared from 2-amino-5-chlorobenzamide (100 mg, 0.59 mmol), triethyl orthobenzoate (264 mg, 267 µL, 1.18 mmol) and acetic acid (106 mg, 101 µL, 1.77 mmol) in absolute ethanol (3 mL) at 110 °C for 24 h. Yield: 147 mg (97%) as white crystals, m.p. 292–293 °C (lit [37] m.p. 295–296 °C); IR (nujol): 3151, 1679 cm^−1^; ^1^H-NMR (400 MHz, DMSO-*d_6_*): δ 12.7 (br s, 1H), 8.18 (dm, *J* = 6.8 Hz, 2H), 8.10 (d, *J* = 2.5 Hz, 1H), 7.87 (dd, *J* = 8.7, 2.5 Hz, 1H), 7.77 (d, *J* = 8.7 Hz, 1H), 7.64–7.53 (complex, 3H); ^13^C-NMR (101 MHz, DMSO-*d_6_*): δ 161.8, 153.3, 148.0, 135.2, 132.9, 132.1, 131.2, 130.2, 129.1, 128.3, 125.3, 122.7; HRMS (ESI): *m/z* for C_14_H_9_^35^ClN_2_O [M + 1]^+^: calcd.: 257.0482; found: 257.0477.

7-Chloro-2-methylquinazolin-4(3*H*)-one (**12a**). This compound was prepared from 2-amino-4-chlorobenzamide (100 mg, 0.59 mmol) triethyl orthoacetate (191 mg, 216 µL, 1.18 mmol) and acetic acid (106 mg, 101 µL, 1.77 mmol) in absolute ethanol (3 mL) at 110 °C for 24 h. Yield: 108 mg (94%) as a light tan powder, m.p. 293–295 °C (lit [38] m.p. 295–297 °C); IR (nujol): 3163, 1674, 1636, 1609 cm^−1^; ^1^H-NMR (400 MHz, DMSO-*d_6_*): δ 12.3 (br s, 1H), 8.06 (d, *J* = 8.5 Hz, 1H), 7.62 (dd, *J* = 1.6 Hz, 1H), 7.48 (d, *J* = 8.5, 1.7 Hz, 1H), 2.35 (s, 3H); ^13^C-NMR (101 MHz, DMSO-*d_6_*): δ 161.1, 156.0, 150.1, 138.8, 127.8, 126.1, 125.7, 119.5, 21.5; HRMS (ESI): *m/z* for C_9_H_7_^35^ClN_2_O [M + 1]^+^: calcd.: 195.0325; found: 195.0322.

7-Chloro-2-ethylquinazolin-4(3*H*)-one (**12b**). This compound was prepared 2-amino-4-chlorobenzamide (100 mg, 0.59 mmol), triethyl orthopropionate (208 mg, 237 µL, 1.18 mmol) and acetic acid (106 mg, 101 µL, 1.77 mmol) in absolute ethanol (3 mL) at 110 °C for 24 h. Yield: 115 mg (93%) as a light tan powder, m.p. 235–237 °C (lit [29] m.p. 205–207 °C); IR (nujol): 3172, 1681, 1620, 1607 cm^−1^; ^1^H-NMR (400 MHz, DMSO-*d_6_*): δ 12.3 (br s, 1H), 8.07 (d, *J* = 8.5 Hz, 1H), 7.65 (dd, *J* = 2.0 Hz, 1H), 7.49 (dd, *J* = 8.5, 2.0 Hz, 1H), 2.62 (q, *J* = 7.5 Hz, 2H), 1.23 (t, *J* = 7.5 Hz, 3H); ^13^C-NMR (101 MHz, DMSO-*d_6_*): δ 161.2, 160.1, 150.1, 138.9, 127.8, 126.2, 126.0, 119.7, 27.9, 11.2; HRMS (ESI): *m/z* for C_10_H_9_^35^ClN_2_O [M + 1]^+^: calcd.: 209.0482; found: 209.0481.

7-Chloro-2-propylquinazolin-4(*3H)*-one (**12c**). This compound was prepared from 2-amino-4-chlorobenzamide (100 mg, 0.59 mmol), triethyl orthobutyrate (224 mg, 255 µL, 1.18 mmol) and acetic acid (106 mg, 101 µL, 1.77 mmol) in absolute ethanol (3 mL) at 110 °C for 24 h. Yield: 117 mg (89%) as a light tan powder, m.p. 214–216 °C; IR (nujol): 3169, 1681, 1613, 1603 cm^−1^; ^1^H-NMR (400 MHz, DMSO-*d_6_*): δ 12.3 (br s, 1H), 8.06 (d, *J* = 8.5 Hz, 1H), 7.65 (dd, *J* = 1.8 Hz, 1H), 7.49 (dd, *J* = 8.5, 1.9 Hz, 1H), 2.57 (t, *J* = 7.4 Hz, 2H), 1.73 (sextet, *J* = 7.4 Hz, 2H), 0.93 (t, *J* = 7.4 Hz, 3H); ^13^C-NMR (101 MHz, DMSO-*d*_6_): δ 161.2, 159.1, 150.1, 138.9, 127.8, 126.2, 125.9, 119.6, 36.4, 20.2, 13.5; HRMS (ESI): *m/z* for C_11_H_11_^35^ClN_2_O [M + 1]^+^: calcd.: 223.0638; found: 223.0635.

7-Chloro-2-phenylquinazolin-4(3*H*)-one (**12d***)*. This compound was prepared from 2-amino-4-chlorobenzamide (100 mg, 0.59 mmol), triethyl orthobenzoate (264 mg, 267 µL, 1.18 mmol) and acetic acid (106 mg, 101 µL, 1.77 mmol) in absolute ethanol (3 mL) at 110 °C for 24 h. Yield: 148 mg (98%) as light yellow crystals, m.p. 276–278 °C (lit [43] m.p. > 300 °C); IR (nujol): 3151, 1679 cm^−1^; ^1^H-NMR (400 MHz, DMSO-*d*_6_): δ 12.7 (br s, 1H), 8.18 (dm, *J* = 6.9 Hz, 2H), 8.15 (d, *J* = 8.5 Hz, 1H), 7.81 (d, *J* = 2.0 Hz, 1H), 7.64–7.53 (complex, 4H); ^13^C-NMR (101 MHz, DMSO-*d_6_*): δ 162.1, 154.3, 150.4, 139.6, 132.8, 132.2, 129.1, 128.42, 128.38, 127.3, 127.1, 120.3; HRMS (ESI): *m/z* for C_14_H_9_^35^ClN_2_O [M + 1]^+^: calcd.: 257.0482; found: 257.0480.

2-Methylpyrido[2,3-*d*]pyrimidin-4(3*H*)-one (**14a**). This compound was prepared from 2-aminonicotinamide (126 mg, 0.92 mmol), triethyl orthoacetate (224 mg, 253 µL, 1.38 mmol) and acetic acid (165 mg, 158 µL, 2.76 mmol) in absolute ethanol (3 mL) at 110 °C for 12 h. Yield: 127 mg (86%) as an off-white solid, m.p. 261–263 °C (lit [14] m.p. 262–264 °C); IR (CHCl_3_): 3207, 1698 cm^−1^; ^1^H-NMR (400 MHz, DMSO-*d*_6_): δ 12.5 (br s, 1H), 8.90 (dd, *J* = 4.6, 1.9 Hz, 1H), 8.45 (dd, *J* = 7.8, 1.9 Hz, 1H), 7.48 (td, *J* = 7.8, 4.6 Hz, 1H), 2.40 (s, 3H); ^13^C-NMR (101 MHz, DMSO-*d*_6_): δ 162.3, 158.9, 157.9, 155.7, 135.3, 121.7, 115.6, 21.8; HRMS (ESI): *m/z* for C_8_H_7_N_3_O [M + 1]^+^: calcd.: 162.0667; found: 162.0671.

2-Ethylpyrido[2,3-*d*]pyrimidin-4(3*H*)-one (**14b**). This compound was prepared from 2-aminonicotinamide (126 mg, 0.92 mmol) and triethyl orthopropionate (244 mg, 278 µL, 1.38 mmol) and acetic acid (165 mg, 158 µL, 2.76 mmol) in absolute ethanol (3 mL) at 110 °C for 12 h. Yield: 148 mg (92%) as an off-white solid, m.p. 197–198 °C; IR (CHCl_3_): 3198, 1674 cm^−1^; ^1^H-NMR (400 MHz, CDCl_3_): δ 11.6 (br s, 1H), 9.02 (dd, *J* = 4.6, 2.1 Hz, 1H), 8.62 (dd, *J* = 7.9, 2.1 Hz, 1H), 7.44 (dd, *J* = 7.9, 4.6 Hz, 1H), 2.91 (q, *J* = 7.6 Hz, 2H), 1.48 (t, *J* = 7.6 Hz, 3H); ^13^C-NMR (101 MHz, CDCl_3_): δ 164.3, 161.2, 159.4, 156.5, 135.9, 122.0, 115.8, 29.2, 11.0; HRMS (ESI): *m/z* for C_9_H_9_N_3_O [M + 1]^+^: calcd.: 176.0824; found: 176.0819.

2-Propylpyrido[2,3-*d*]pyrimidin-4(3*H*)-one (**14c**). This compound was prepared from 2-aminonicotinamide (126 mg, 0.92 mmol), triethyl orthobutyrate (262 mg, 298 µL, 1.38 mmol) and acetic acid (165 mg, 158 µL, 2.76 mmol) in absolute ethanol (3 mL) at 110 °C for 12 h. Yield: 164 mg (87%) as a tan solid, m.p. 168–170 °C; IR (CHCl_3_): 3205, 1674 cm^−1^; ^1^H-NMR (400 MHz, CDCl_3_): δ 11.4 (br s, 1H), 9.00 (dd, *J* = 4.6, 2.1 Hz, 1H), 8.60 (dd, *J* = 7.9, 2.1 Hz, 1H), 7.43 (dd, *J* = 7.9, 4.6 Hz, 1H), 2.81 (t, *J* = 7.5 Hz, 2H), 1.97 (sextet, *J* = 7.5 Hz, 2H), 1.07 (t, *J* = 7.5 Hz, 3H); ^13^C-NMR (101 MHz, CDCl_3_): δ 164.5, 160.5, 159.4, 156.5, 135.9, 122.0, 115.7, 37.7, 20.5, 13.7; HRMS (ESI): *m/z* for C_10_H_11_N_3_O [M + 1]^+^: calcd.: 190.0980; found: 190.0977.

2-Phenylpyrido[2,3-*d*]pyrimidin-4(3*H*)-one (**14d**). This compound was prepared from 2-aminonicotinamide (126 mg, 0.92 mmol) and triethyl orthobenzoate (309 mg, 312 µL, 1.38 mmol) and acetic acid (165 mg, 158 µL, 2.76 mmol) in absolute ethanol (3 mL) at 110 °C for 12 h. Yield: 164 mg (80%) as an off-white solid, m.p. 181–182 °C (lit [35] m.p. 178–180 °C); IR (CHCl_3_): 3210, 1672 cm^−1^; ^1^H-NMR (400 MHz, DMSO-*d_6_*): δ 12.8 (br s, 1H), 8.99 (dd, *J* = 4.5, 2.0 Hz, 1H), 8.54 (dd, J = 7.8, 2.0 Hz, 1H), 8.25 (d, *J* = 8.1 Hz, 2H), 7.67–7.56 (complex, 3H), 7.55 (dd, *J* = 7.8, 4.5 Hz, 1H); ^13^C-NMR (101 MHz, DMSO-*d_6_*): δ 163.4, 159.2, 156.6, 155.9, 136.0, 132.9, 132.4, 129.1, 128.5, 122.7, 116.6; HRMS (ESI): *m/z* for C_13_H_9_N_3_O [M + 1]^+^: calcd.: 224.0824; found: 224.0820.

2,3-Dimethylquinazolin-4(3*H*)-one (**16a**). This compound was prepared from 2-amino-*N*-methylbenzamide (100 mg, 0.67 mmol), triethyl orthoacetate (224 mg, 253 µL, 1.38 mmol) and acetic acid (121 mg, 115 µL, 2.01 mmol) in absolute ethanol (2 mL) at 110 °C for 12 h. Yield: 102 mg (87%) as a light tan solid, m.p. 108–109 °C (lit [19] m.p. 112–114 °C); IR (CHCl_3_): 1661 cm^−1^; ^1^H-NMR (400 MHz, CDCl_3_): δ 8.27 (dd, *J* = 8.0, 1.2 Hz, 1H), 7.72 (ddd, *J* = 8.3, 7.2, 1.5 Hz, 1H), 7.61 (d, *J* = 8.0 Hz, 1H), 7.44 (td, *J* = 8.0, 1.0 Hz, 1H), 3.63 (s, 3H), 2.63 (s, 3H); ^13^C-NMR (101 MHz, CDCl_3_): δ 162.5, 154.6, 147.4, 134.3, 126.9, 126.8, 126.5, 120.4, 31.2, 23.8; HRMS (ESI): *m/z* for C_10_H_10_N_2_O [M + 1]^+^: calcd.: 175.0871; found: 175.0874.

2-Ethyl-3-methylquinazolin-4(3*H*)-one (**16b**). This compound was prepared from 2-amino-*N*-methylbenzamide (100 mg, 0.67 mmol), orthopropionate (236 mg, 269 µL, 1.34 mmol) and acetic acid (121 mg, 115 µL, 2.01 mmol) in absolute ethanol (2 mL) at 110 °C for 24 h. Yield: 105 mg (83%) as a light tan solid, m.p. 118–120 °C (lit [50] m.p. 120–121 °C); IR (CHCl_3_): 1684 cm^−1^; ^1^H-NMR (400 MHz, CDCl_3_): δ 8.26 (dd, *J* = 8.1, 1.2 Hz, 1H), 7.72 (ddd, (*J* = 8.2, 7.0, 1.5 Hz, 1H), 7.65 (d, *J* = 8.0 Hz, 1H), 7.43 (ddd, *J* = 8.2, 7.0, 1.3 Hz, 1H), 3.64 (s, 3H), 2.87 (q, *J* = 7.4 Hz, 2H), 1.41 (t, *J* = 7.4 Hz, 3H); ^13^C-NMR (101 MHz, CDCl_3_): δ 162.6, 157.8, 147.3, 134.0, 126.9, 126.7, 126.3, 120.2, 30.3, 28.9, 11.1; HRMS (ESI): *m/z* for C_11_H_12_N_2_O [M + 1]^+^; calcd.: 189.1028; found: 189.1025.

2-Phenyl-3-methylquinazolin-4(3*H*)-one (**16d**). This compound was prepared from 2-amino-*N*-methylbenzamide (100 mg, 0.67 mmol), triethyl orthobenzoate (300 mg, 303 µL, 1.34 mmol) and acetic acid (121 mg, 115 µL, 2.01 mmol) in absolute ethanol (2 mL) at 110 °C for 48 h. Yield: 140 mg (89%) as a light tan solid, m.p. 133–135 °C (lit [19] m.p. 134–138 °C); IR (CHCl_3_): 1674 cm^−1^; ^1^H-NMR (400 MHz, CDCl_3_): δ 8.34 (d, *J* = 7.8 Hz, 1H), 7.79 (complex, 2H), 7.60–7.49 (complex, 6H), 3.50 (s, 3H); ^13^C-NMR (101 MHz, CDCl_3_): δ 162.7, 156.1, 147.3, 135.4, 134.3, 130.1, 128.9, 128.0, 127.5, 127.0, 126.7, 120.6, 34.3; HRMS (ESI): *m/z* for C_15_H_12_N_2_O [M + 1]^+^: calcd.: 237.1028; found: 237.1024.

2-Methyl-3-phenylquinazolin-4(3*H*)-one (**18a**). This compound was prepared from 2-amino-*N*-phenylbenzamide (100 mg, 0.47 mmol), triethyl orthoacetate (228 mg, 258 µL, 1.41 mmol) and acetic acid (85 mg, 81 µL, 1.41 mmol) in absolute ethanol (2 mL) at 110 °C for 24 h. Yield: 105 mg (95%) as a white solid, m.p. 142–143 °C (lit [21] m.p. 142–144 °C); IR (CHCl_3_): 1684 cm^−1^; ^1^H-NMR (400 MHz, CDCl_3_): δ 8.27 (dd, *J* = 7.9, 0.7 Hz, 1H), 7.77 (td, *J* = 8.1, 1.3 Hz, 1H), 7.68 (d, *J* = 8.1 Hz, 1H), 7.60–7.44 (complex, 4H), 7.27 (d, *J* = 7.1 Hz, 2H), 2.25 (s, 3H); ^13^C-NMR (101 MHz, CDCl_3_): δ 162.3, 154.2, 147.5, 137.8, 134.6, 130.0, 129.3, 128.0, 127.1, 126.8, 126.6, 120.8, 24.4; HRMS (ESI): *m/z* for C_15_H_12_N_2_O [M + 1]^+^: calcd.: 237.1028; found: 237.1023.

2-Ethyl-3-phenylquinazolin-4(3*H*)-one (**18b**). This compound was prepared from 2-amino-*N*-phenylbenzamide (100 mg, 0.47 mmol), triethyl orthopropionate (248 mg, 283 µL, 1.41 mmol) and acetic acid (85 mg, 81 µL, 1.41 mmol) in absolute ethanol (2 mL) at 110 °C for 24 h. Yield: 108 mg (92%) as an off-white solid, m.p. 120–122 °C (lit [21] m.p. 124–125 °C); IR (CHCl_3_): 1684 cm^−1^; ^1^H-NMR (400 MHz, CDCl_3_): δ 8.28 (dd, *J* = 8.0, 1.4 Hz, 1H), 7.77 (ddd, *J* = 8.2, 6.8, 1.5 Hz, 1H), 7.73 (dd, *J* = 8.1, 1.5 Hz, 1H), 7.59–7.47 (complex, 4H), 7.27 (m, 2H), 2.45 (q, *J* = 7.4 Hz, 2H), 1.22 (t, *J* = 7.4 Hz, 3H); ^13^C-NMR (101 MHz, CDCl_3_): δ 162.5, 157.8, 147.6, 137.4, 134.5, 129.9, 129.2, 128.3, 127.1, 127.0, 126.6, 120.8, 29.3, 11.7; HRMS (ESI): *m/z* for C_16_H_14_N_2_O [M + 1]^+^: calcd.: 251.1184; found: 251.1179.

2,3-Diphenylquinazolin-4(3*H*)-one (**18d**). This compound was prepared from 2-amino-*N*-phenylbenzamide (100 mg, 0.47 mmol), triethyl orthobenzoate (316 mg, 319 µL, 1.41 mmol) and acetic acid (85 mg, 81 µL, 1.41 mmol) in absolute ethanol (2 mL) at 110 °C for 24 h. Yield: 132 mg (95%) as an off-white solid, m.p. 154–156 °C (lit [21] m.p. 154–155 °C); IR (CHCl_3_): 1692, 1664 cm^−1^; ^1^H-NMR (400 MHz, CDCl_3_): δ 8.37 (d, *J* = 8.0 Hz, 1H), 7.85–7.78 (complex, 2H), 7.54 (ddd, *J* = 8.1, 5.6, 2.8 Hz, 1H), 7.36–7.13 (complex, 10H); ^13^C-NMR (101 MHz, CDCl_3_): δ 162.3, 155.2, 147.5, 137.7, 135.5, 134.7, 129.3, 129.1, 129.0 (2C), 128.4, 128.0, 127.8, 127.3, 127.2, 121.0; HRMS (ESI): *m/z* for C_20_H_14_N_2_O [M + 1]: calcd.: 299.1184; found: 299.1180.

3-Amino-2-methylquinazolin-4(3*H*)-one (**20a**). This compound was prepared from 2-aminobenzhydrazide (100 mg, 0.67 mmol), triethyl orthoacetate (217 mg, 245 µL, 1.34 mmol) and acetic acid (121 mg, 115 µL, 2.01 mmol) in absolute ethanol (2 mL) at 110 °C for 24 h. Yield: 97 mg (82%) a tan solid, m.p. 147–149 °C (lit [51] m.p. 146–148 °C); IR (CHCl_3_): 3455, 3413, 1635 cm^−1^; ^1^H-NMR (400 MHz, CDCl_3_): δ 7.72 (dd, *J* = 8.0, 1.2 Hz, 1H), 7.26 (td, *J* = 8.1, 1.2 Hz, 1H), 6.79 (d, *J* = 8.1 Hz, 1H), 6.75 (td, *J* = 8.1, 0.9 Hz, 1H), 5.81 (br s, 2H), 2.61 (s, 3H); ^13^C-NMR (101 MHz, CDCl_3_): δ 165.0, 161.9, 147.0, 132.4, 127.9, 116.9, 116.3, 106.0, 11.1; HRMS (ESI): *m/z* for C_9_H_9_N_3_O [M + 1]^+^: calcd.: 176.0824; found: 177.0898.

3-Amino-2-ethylquinazolin-4(3*H*)-one (**20b**). This compound was prepared from 2-aminobenzhydrazide (100 mg, 0.67 mmol), triethyl orthopropionate (235 mg, 269 µL, 1.34 mmol) and acetic acid (121 mg, 115 µL, 2.01 mmol) in absolute ethanol (2 mL) at 110 °C for 24 h. Yield: 100 mg (80%) as a tan solid, m.p. 116–117 °C (lit [52] m.p. 121.5–122.5 °C); IR (CHCl_3_): 3421, 3320, 1627 cm^−1^; ^1^H-NMR (400 MHz, CDCl_3_): δ 7.73 (dd, *J* = 7.9, 1.5 Hz, 1H), 7.25 (td, *J* = 8.1, 1.6 Hz, 1H), 6.79 (d, *J* = 8.2 Hz, 1H), 6.74 (t, *J* = 8.1 Hz, 1H), 5.82 (br s, 2H), 2.95 (q, *J* = 7.6 Hz, 2H), 1.44 (t, *J* = 7.6 Hz, 3H); ^13^C-NMR (101 MHz, CDCl_3_): δ 166.0, 164.7, 146.8, 132.2, 127.7, 116.7, 116.1, 106.0, 19.0, 10.9; HRMS (ESI): *m/z* for C_10_H_11_N_3_O [M + 1]^+^: calcd.: 190.0980; found: 190.0978.

3-Amino-2-phenylquinazolin-4(3*H*)-one (**20d**). This compound was prepared from 2-aminobenzhydrazide (100 mg, 0.67 mmol), triethyl orthobenzoate (300 mg, 303 µL, 1.34 mmol) and acetic acid (121 mg, 115 µL, 2.01 mmol) in absolute ethanol (2 mL) at 110 °C for 24 h. Yield: 151 mg (95%) as a tan solid, m.p. 160–161 °C (lit [53] m.p. 181–182 °C); IR (CHCl_3_): 3435, 3316, 1612 cm^−1^; ^1^H-NMR (400 MHz, CDCl_3_): δ 8.15 (m, 2H), 7.87 (dd, *J* = 8.0, 1.5 Hz, 1H), 7.55 (m, 3H), 7.31 (ddd, *J* = 8.4, 7.2, 1.5 Hz, 1H), 6.82 (d, *J* = 8.4 Hz, 1H), 6.80 (td, *J* = 7.9, 1.1 Hz, 1H), 5.90 (br s, 2H); ^13^C-NMR (101 MHz, CDCl_3_): δ 164.6, 162.8, 147.1, 132.5, 131.7, 129.1, 127.8, 126.9, 123.9, 116.8, 116.2, 105.8; HRMS (ESI): *m/z* for C_14_H_11_N_3_O [M + 1]^+^: calcd.: 238.0980; found: 238.0974.

#### 3.2.3. Method 3: General Procedure to Prepare 5,6-dihydropyrimidin-4(3*H*)-ones

The orthoester (1.5 equiv) was added to a mixture of the 3-amino-2,2-dimethylpropionamide (1.0 equiv) in anhydrous ethanol (3 mL). Glacial acetic acid (2 equiv) was added and the reaction was heated at reflux for 24 h. The reaction mixture was cooled and concentrated under vacuum. The product was hygroscopic and was best crystallized by adding purified chloroform and allowing it to sit under nitrogen for 4–7 days. Filtration and drying yielded the pure product.

2,5,5-Trimethyl-5,6-dihydropyrimidin-4(3*H*)-one (**22a**). This compound was prepared from 3-amino-2,2-dimethylpropionamide (100 mg, 0.86 mmol), triethyl orthoacetate (209 mg, 236 µL, 1.29 mmol) and acetic acid (103 mg, 99 µL, 1.72 mmol) in absolute ethanol (3 mL) at 110 °C for 24 h. Yield: 60 mg (50%) as a white solid, m.p. 196–198 °C; IR (nujol): 3223, 1669 cm^−1^; ^1^H-NMR (400 MHz, D_2_O): δ 3.35 (s, 2H), 2.23 (s, 3H), 1.16 (s, 6H), NH exchanged; ^13^C-NMR (101 MHz, D_2_O): δ 184.4, 165.3, 43.0, 23.3 (2C), 18.4; HRMS (ESI): *m/z* for C_7_H_12_N_2_O [M + 1]^+^: calcd.: 141.1028; found: 141.1028.

2-Ethyl-5,5-dimethyl-5,6-dihydropyrimidin-4(3*H*)-one (**22b**). This compound was prepared from 3-amino-2,2-dimethylpropionamide (100 mg, 0.86 mmol), triethyl orthopropionate (227 mg, 259 µL, 1.29 mmol) and acetic acid (103 mg, 99 µL, 1.72 mmol) in absolute ethanol (3 mL) at 110 °C for 24 h. Yield: 59 mg (44%) as a white solid, m.p. 185–187 °C; IR (nujol): 3184, 1642 cm^−1^; ^1^H-NMR (400 MHz, D_2_O): δ 3.36 (s, 2H), 2.50 (q, *J* = 7.7 Hz, 2H), 1.24 (t, *J* = 7.7 Hz, 3H), 1.15 (s, 6H), NH exchanged; ^13^C-NMR (101 MHz, D_2_O): δ 184.5, 169.7, 51.0, 43.8, 26.5, 23.3 (2C), 10.7; HRMS (ESI): *m/z* for C_8_H_14_N_2_O [M + 1]^+^: calcd.: 155.1184; found: 155.1187.

5,5-Dimethyl-2-propyl-5,6-dihydropyrimidin-4(3*H*)-one (**22c**). This compound was prepared from 3-amino-2,2-dimethylpropionamide (100 mg, 0.86 mmol), triethyl orthobutyrate (245 mg, 279 µL, 1.29 mmol) and acetic acid (103 mg, 99 µL, 1.72 mmol) in absolute ethanol (3 mL) at 110 °C for 24 h. Yield: 52 mg (36%) as a white solid, m.p. 118–120 °C; IR (nujol): 3199, 1652 cm^−1^; ^1^H-NMR (400 MHz, D_2_O): δ 3.37 (s, 2H), 2.46 (t, *J* = 7.4 Hz, 2H), 1.69 (sextet, *J* = 7.4 Hz, 2H), 1.16 (s, 6H), 0.96 (t, *J* = 7.4 Hz, 3H), NH exchanged; ^13^C-NMR (101 MHz, D_2_O): δ 184.4, 168.5, 51.0, 43.8, 34.5, 23.3 (2C), 20.2, 12.3; HRMS (ESI): *m/z* for C_9_H_16_N_2_O [M + 1]^+^: calcd.: 169.1341; found: 169.1339.

5,5-Dimethyl-2-phenyl-5,6-dihydropyrimidin-4(3*H*)-one (**22d**). This compound was prepared from 3-amino-2,2-dimethylpropionamide (100 mg, 0.86 mmol), triethyl orthobenzoate (289 mg, 292 µL, 1.29 mmol) and acetic acid (103 mg, 99 µL, 1.72 mmol) in absolute ethanol (3 mL) at 110 °C for 24 h. Yield: 126 mg (72%) as a white solid, m.p. 153–155 °C; IR (nujol): 3231, 1700, 1651 cm^−1^; ^1^H-NMR (400 MHz, CDCl_3_): δ 8.46 (br s, 1H), 7.78 (d, *J* = 7.6 Hz, 2H), 7.53–7.43 (complex, 2H), 3.62 (s, 2H), 1.21 (s, 6H), NH exchanged; ^13^C-NMR (101 MHz, CDCl_3_): δ 177.2, 151.5, 133.3, 131.4, 129.0, 126.4, 57.7, 36.5, 23.4 (2C); HRMS (ESI): *m/z* for C_12_H_14_N_2_O [M + 1]^+^: calcd.: 203.1184; found: 203.1180.

## 4. Conclusions

We have developed an efficient strategy for the synthesis of 2-alkyl- and 2-arylquinazolin-4(3*H*)-ones. The procedure is straightforward, and gives the desired heterocycles in yields ≥80% without contamination by residual metals or Lewis acids. Although almost all of the compounds have been reported previously, the current work provides a unified approach that allows the convenient preparation of a broad range of targets derived from 2-aminobenzamides; ring-substituted 2-aminobenzamides, 2-aminonicotinamides, 2-amino-*N*-methyl (and *N*-phenyl) benzamides; as well as 2-aminobenzhydrazides. The conditions are mild and purification of the final products is easily accomplished. Additionally, we have extended the method to the synthesis of 5,6-dihydropyrimidin-4(3*H*)-ones, although these compounds proved difficult to purify due to their hygroscopic nature. The only limitation to our approach is that relatively few substituted orthoesters are available commercially. Nevertheless, the simplicity and high yields provided by the current procedure, as well as its ability to provide the target heterocycles free of contaminants, makes this method an important contribution to the field.

## Figures and Tables

**Table 1:**
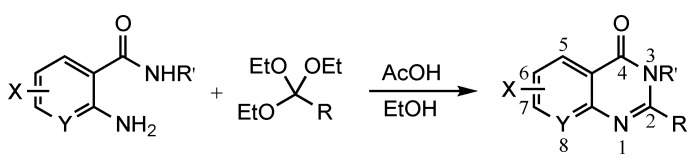
Synthesis of quinazolin-4(3*H*)-ones.

Substrate	Product	R	Method ^a^	Yield (%)
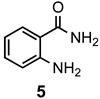	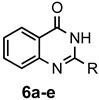	**a**: Me **b**: Et **c**: Pr **d**: P h**e**: H	1 1 1 1 1	92 89 93 81 87
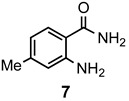	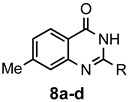	**a**: Me **b**: Et **c**: Pr **d**: Ph	2 2 2 2	90 91 ND ^b^ 84
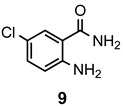	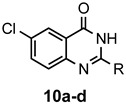	**a**: Me **b**: Et **c**: Pr **d**: Ph	2 2 2 1(2)	92 92 88 33(97)
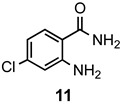	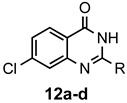	**a**: Me **b**: Et **c**: Pr **d**: Ph	2 2 2 1(2)	94 93 89 7(98)
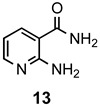	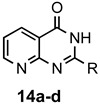	**a**: Me **b**: Et **c**: Pr **d**: Ph	1(2) 1(2) 1(2) 1(2)	71(86) 70(92) 55(87) 68(80)
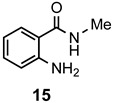	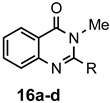	**a**: Me **b**: Et **c**: Pr **d**: Ph	2 2 2 2	87 83 ND ^b^ 89
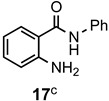	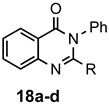	**a**: Me **b**: Et **c**: Pr **d**: Ph	2 2 2 2	95 92 ND ^b^ 95
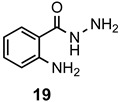	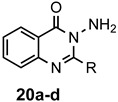	**a**: Me **b**: Et **c**: Pr **d**: Ph	1(2) 2 2 2	21(82) 80 ND ^b^ 95

^a^ For 1 equiv of the 2-aminobenzamide: Method 1: 1.5 equiv of orthoester, 2 equiv of AcOH, 3 mL of EtOH, 78 °C, 12–24 h; Method 2: 2–3 equiv of orthoester, 3 equiv of AcOH, 3 mL of EtOH, 110 °C, pressure tube, 12–72 h; ^b^ ND = not done; ^c^ Prepared from 2-nitrobenzoic acid as described in the experimental section.

## Supplementary Materials

The following are available online. Electronic Supplementary Information (ESI) available: Copies of ^1^H and ^13^C-NMR spectra of the final products.
