# Supplementary material for: Quinazolin-4(3H)-ones and 5,6-Dihydropyrimidin-4(3H)-ones from β-Aminoamides and Orthoesters
Source: Molecules. 2018 Nov 9;23(11):2925. doi: 10.3390/molecules23112925 (PMC6278269; doi:10.3390/molecules23112925)

Quinazolin-4(3*H*)-ones and 5,6-dihydropyrimidin-4(3*H*)-ones from β-aminoamides and orthoesters

Joshua T. Gavin, Joel K. Annor-Gyamfi and Richard A. Bunce*
Department of Chemistry, Oklahoma State University, Stillwater, OK 74078-3071

Supplementary Information

Copies of ^{1}H and ^{13}C NMR spectra for quinazolin-4(3*H*)-ones and
5,6-dihydropyrimidin-4(3*H*)-ones.. 1-34

[1]H Spectrum of 2-Methylquinazolin-4(3*H*)-one (**6a**)

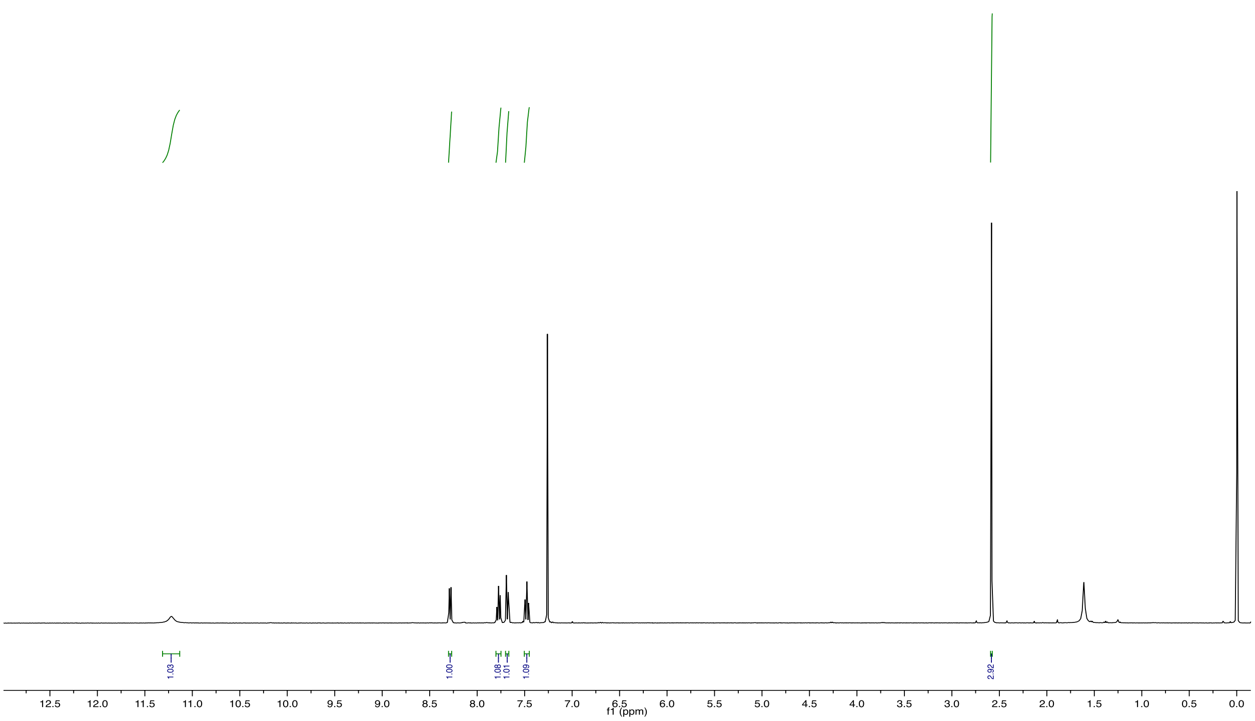

[13]C Spectrum of **6a**

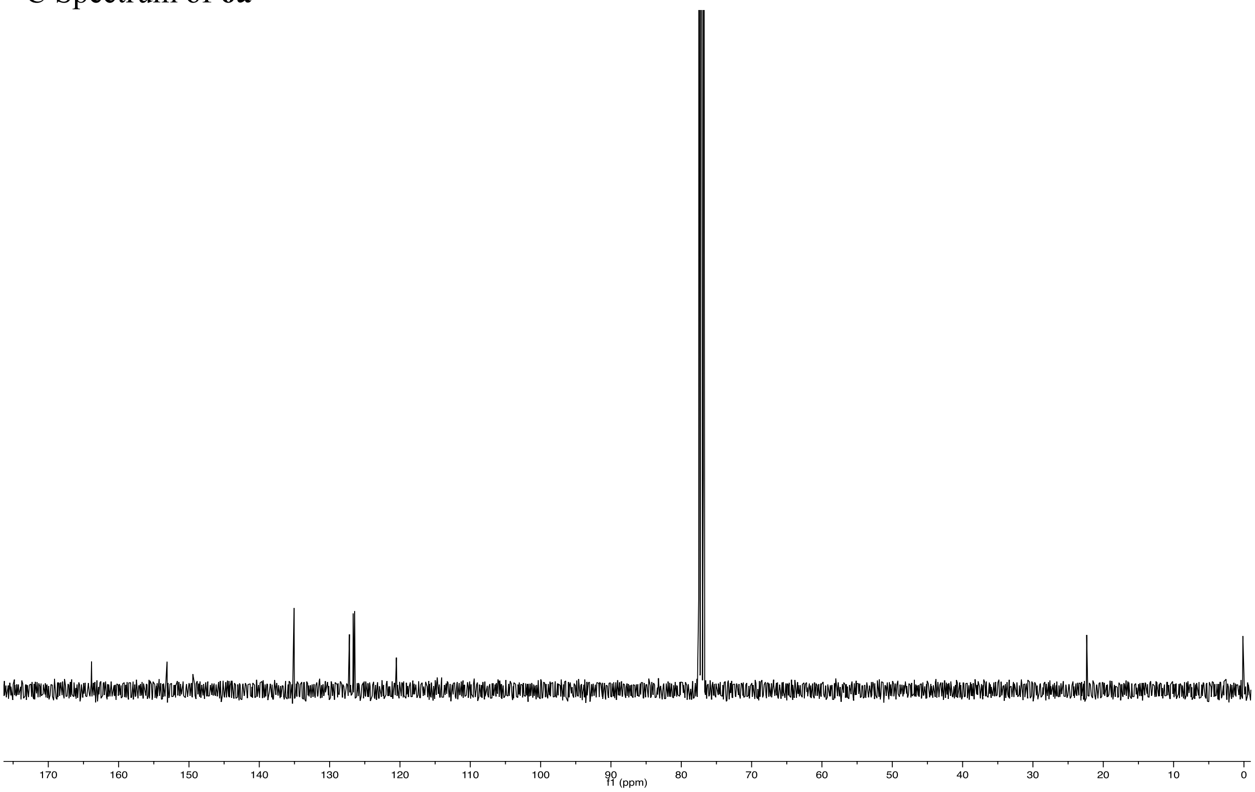

[1]H Spectrum of 2-Ethylquinazolin-4(3*H*)-one (**6b**)

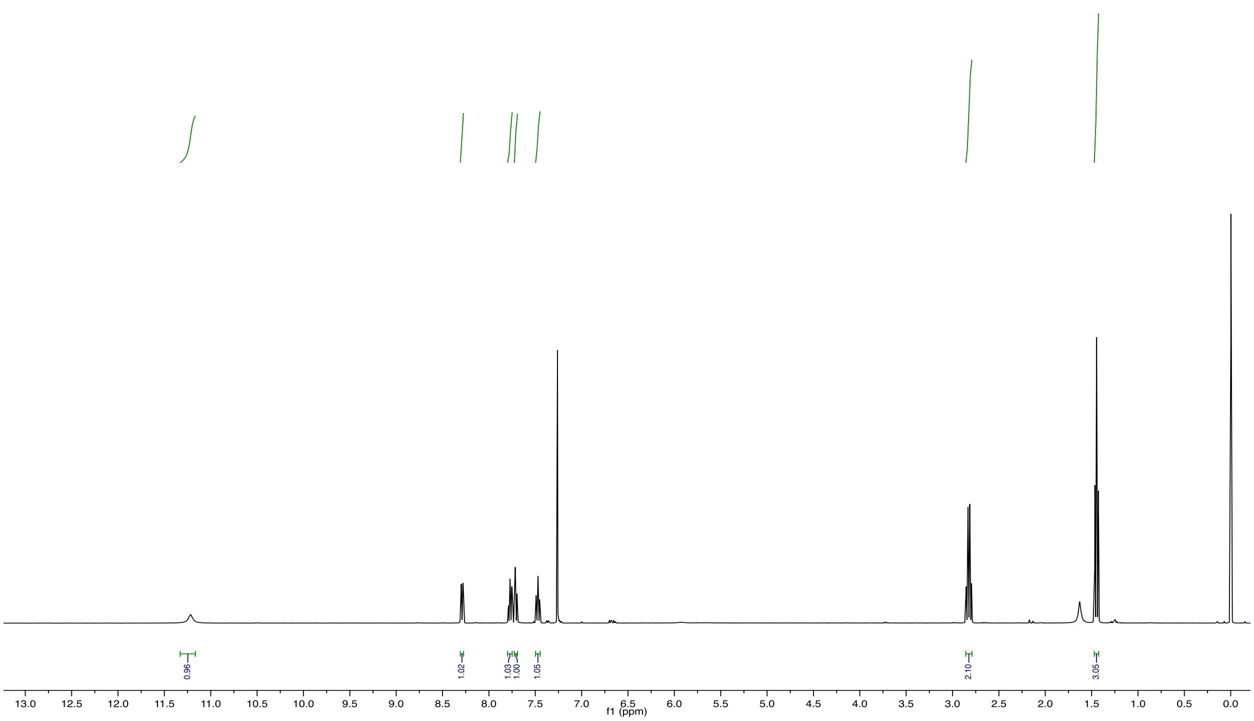

[13]C Spectrum of **6b**

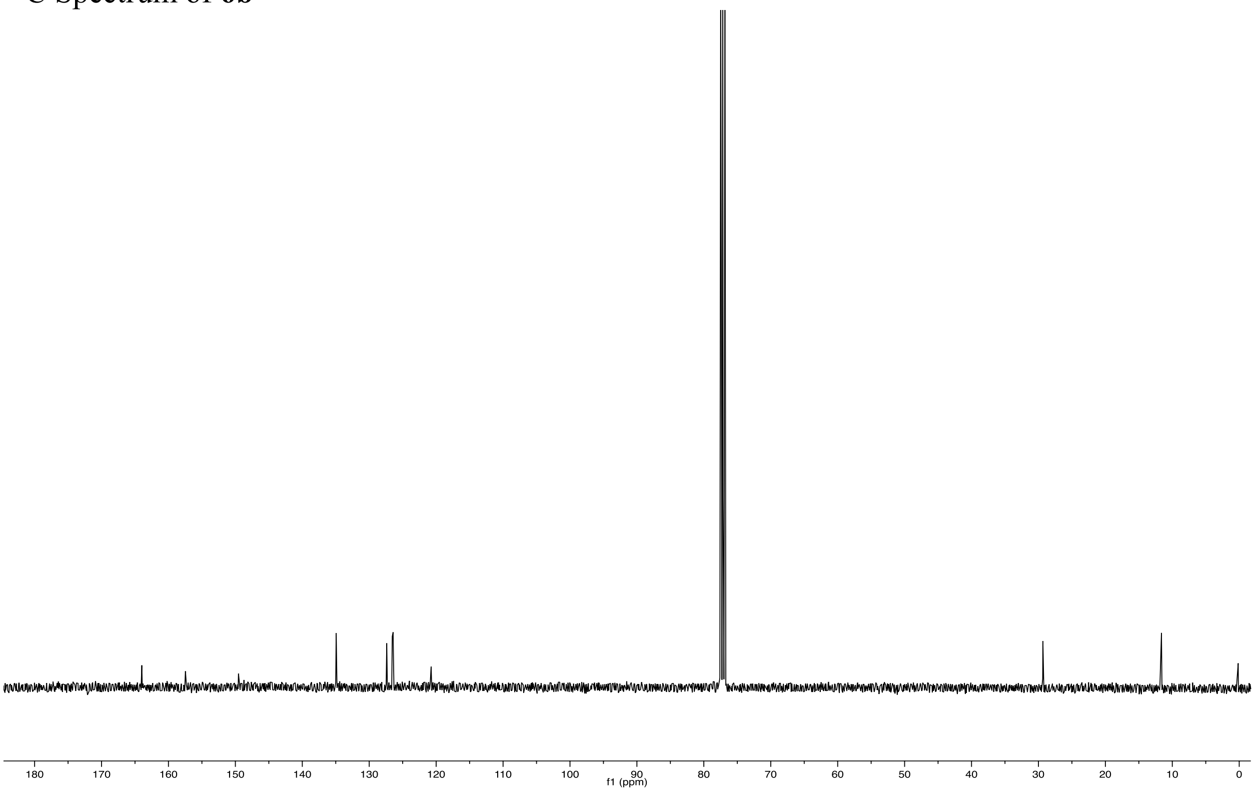

[1]H Spectrum of 2-Propylquinazolin-4(3*H*)-one (**6c**)

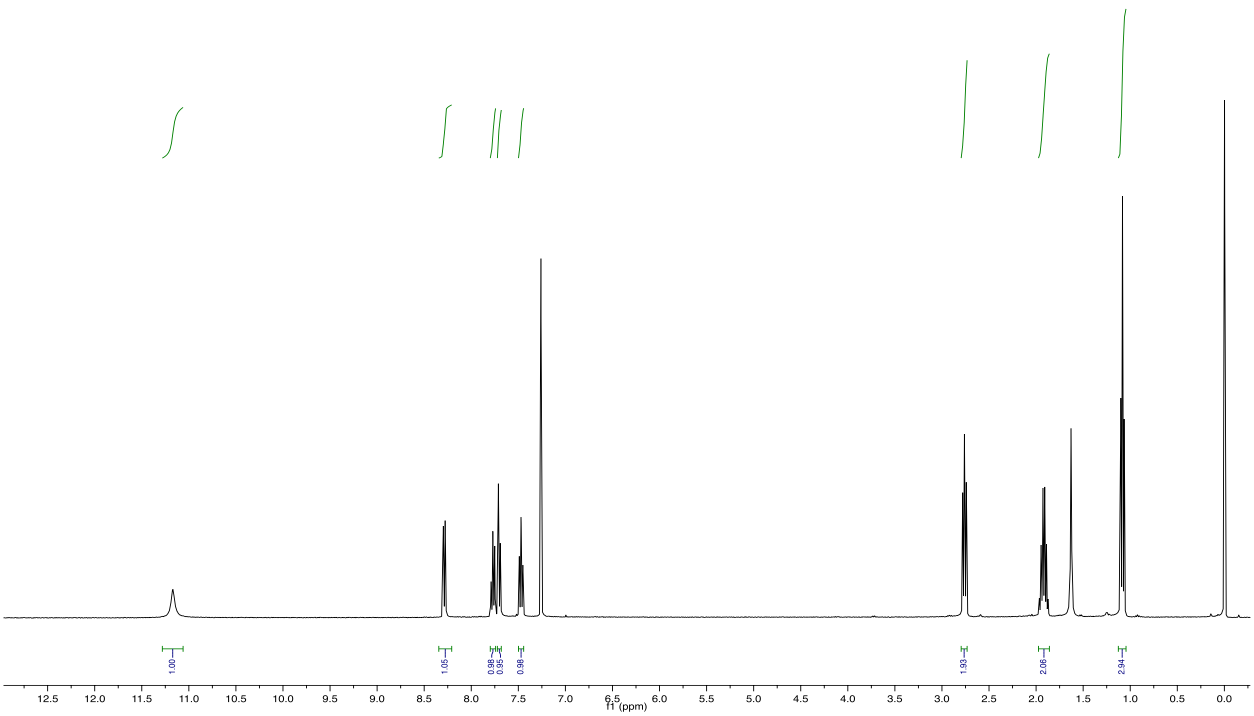

[13]C Spectrum of **6c**

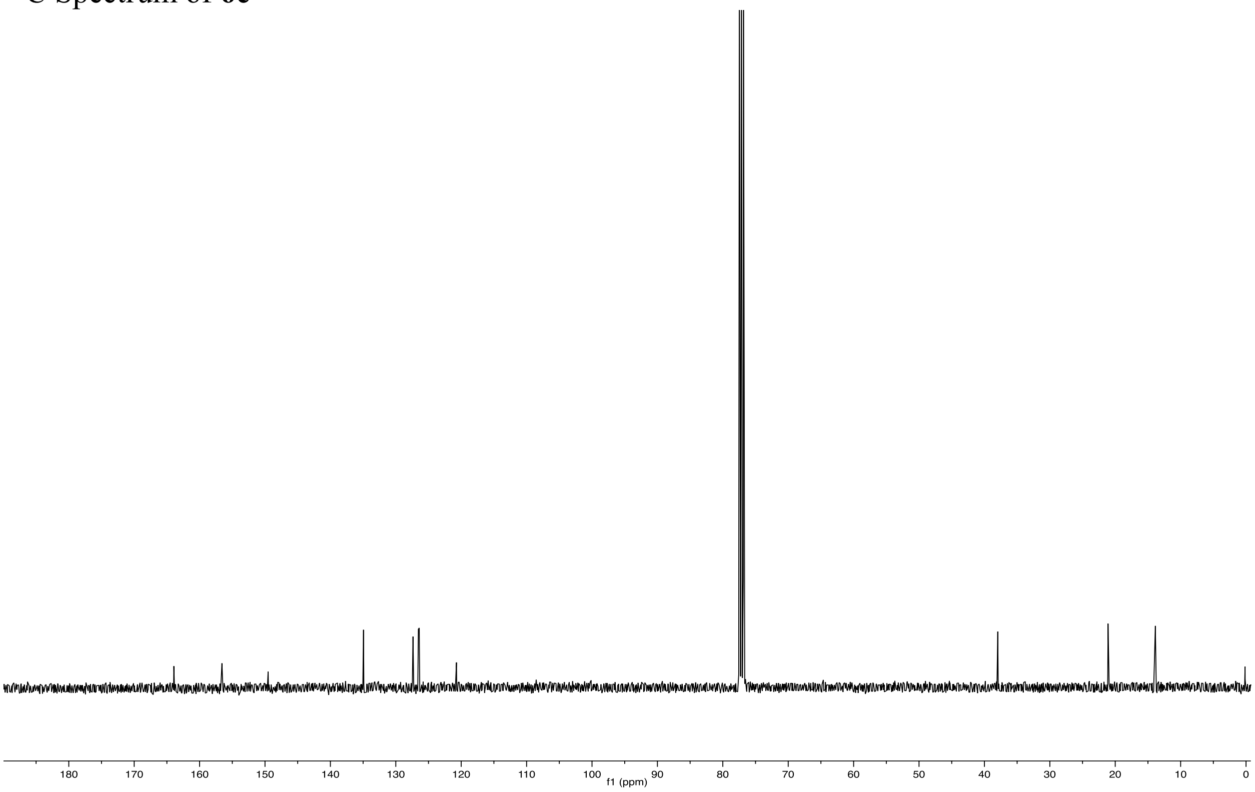

[1]H Spectrum of 2-Phenylquinazolin-4(3*H*)-one (**6d**)

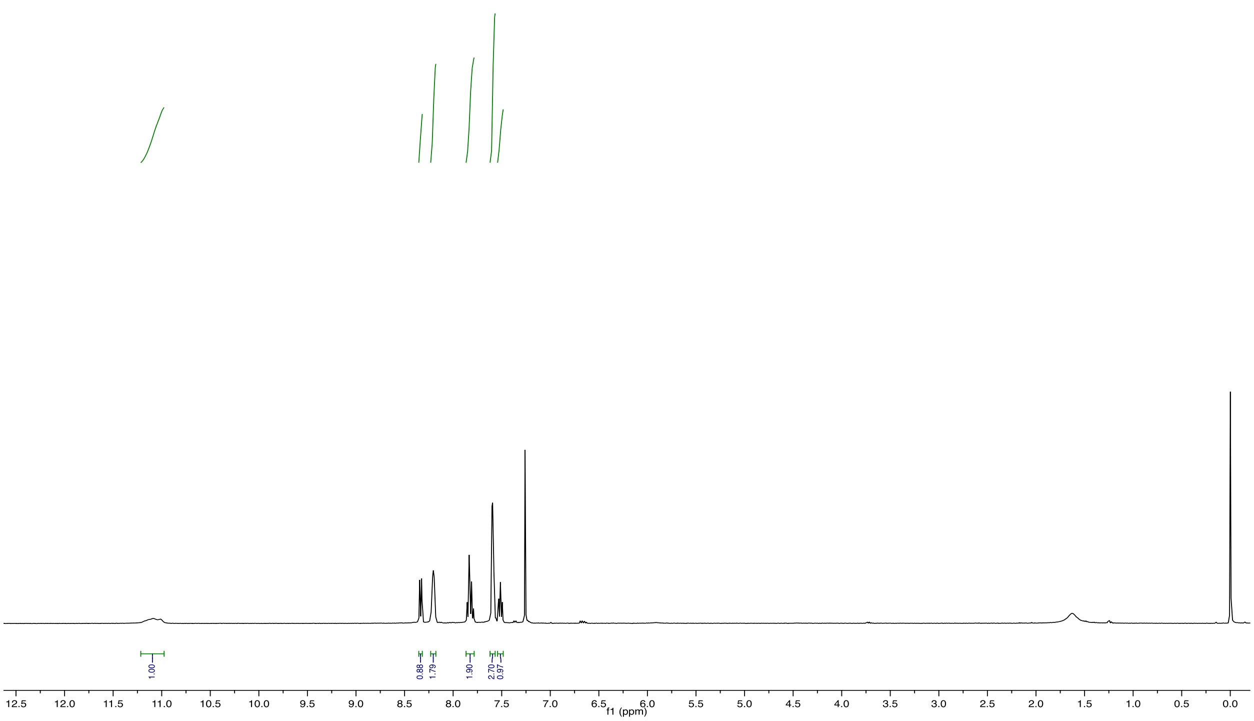

[13]C Spectrum of **6d**

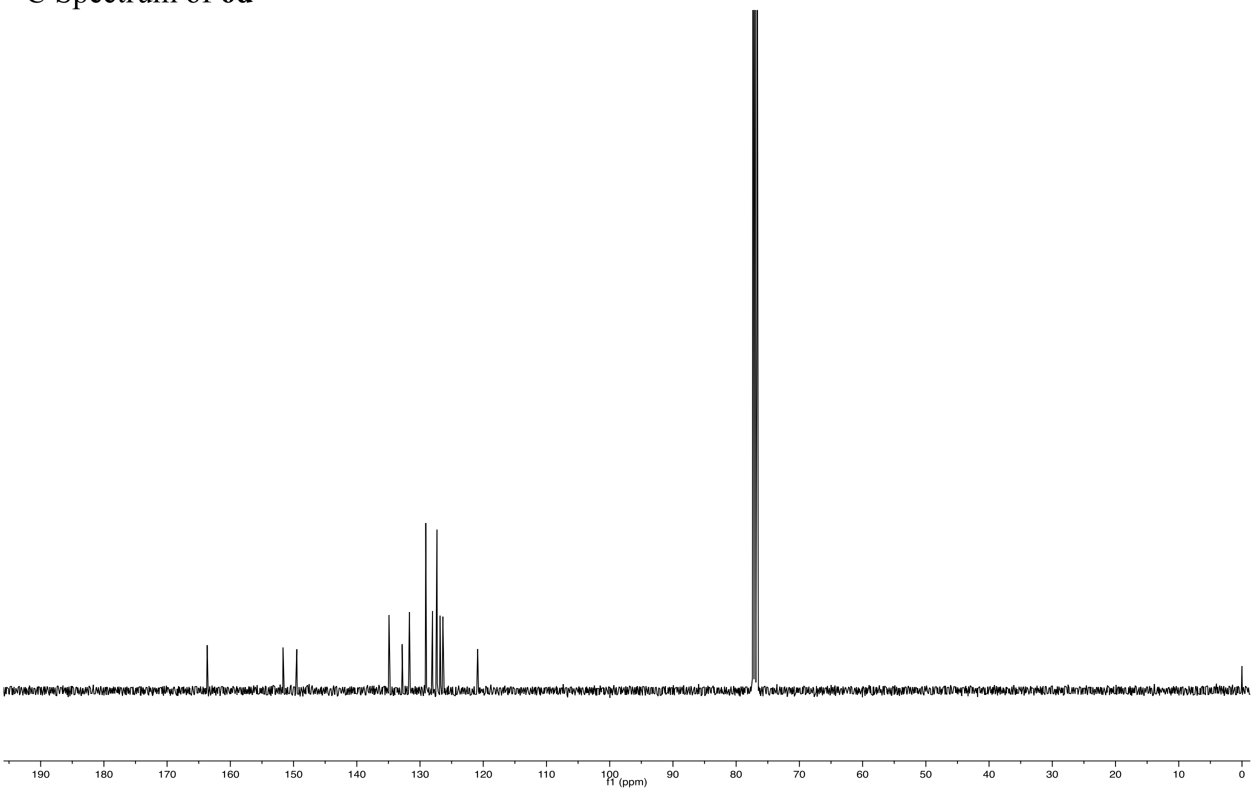

^{1}H Spectrum of Quinazolin-4(3*H*)-one (**6e**)

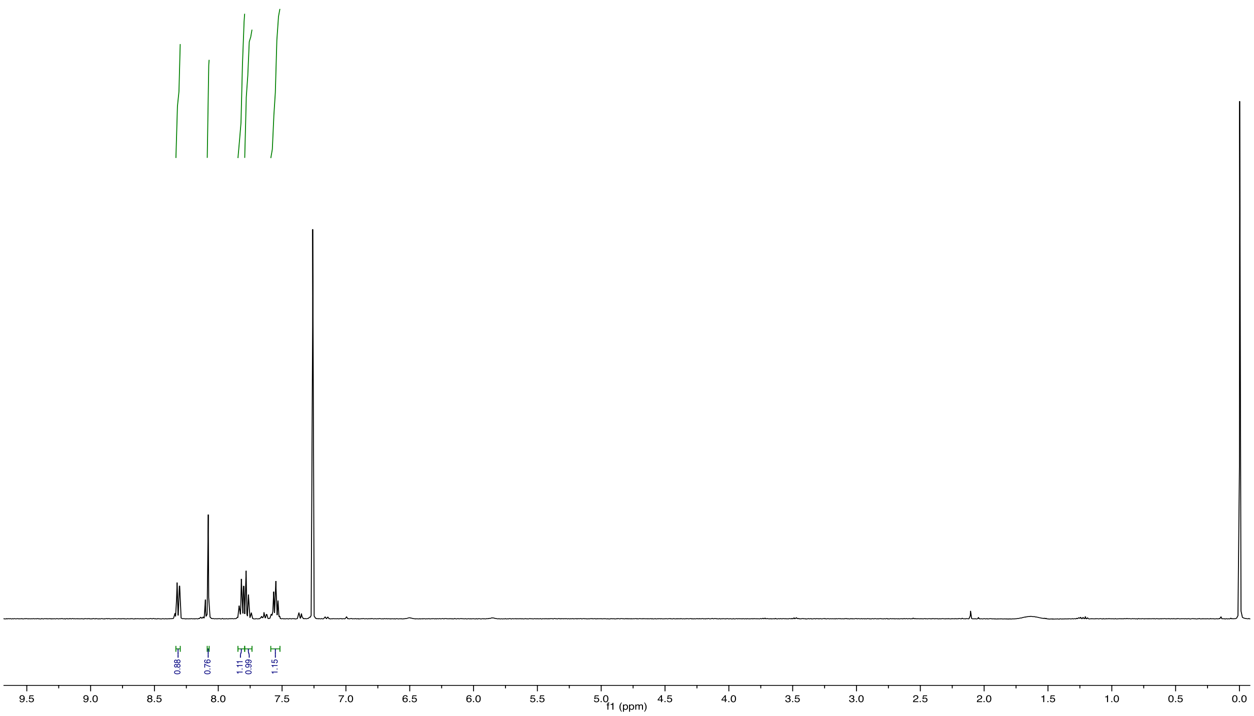

^{13}C Spectrum of **6e**

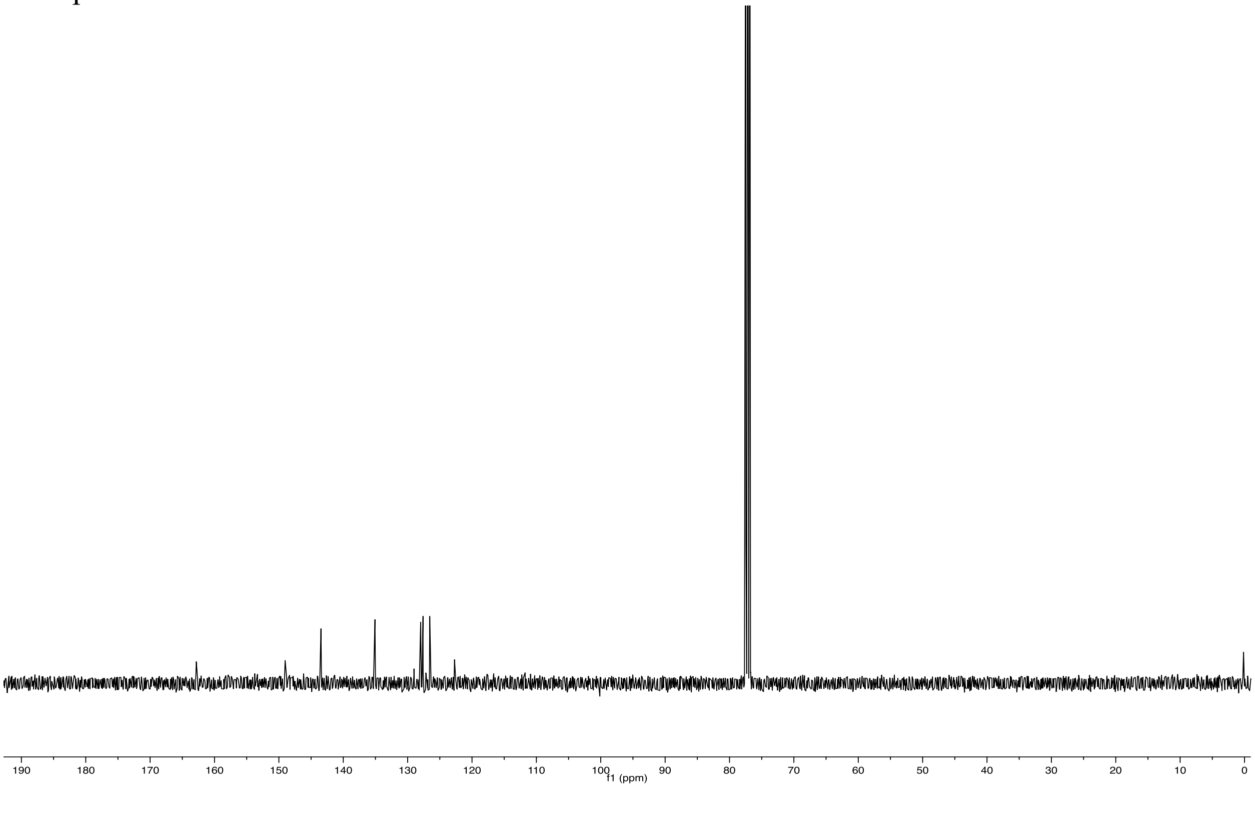

[1]H Spectrum of 2,7-Dimethylquinazolin-4(3*H*)-one (**8a**)

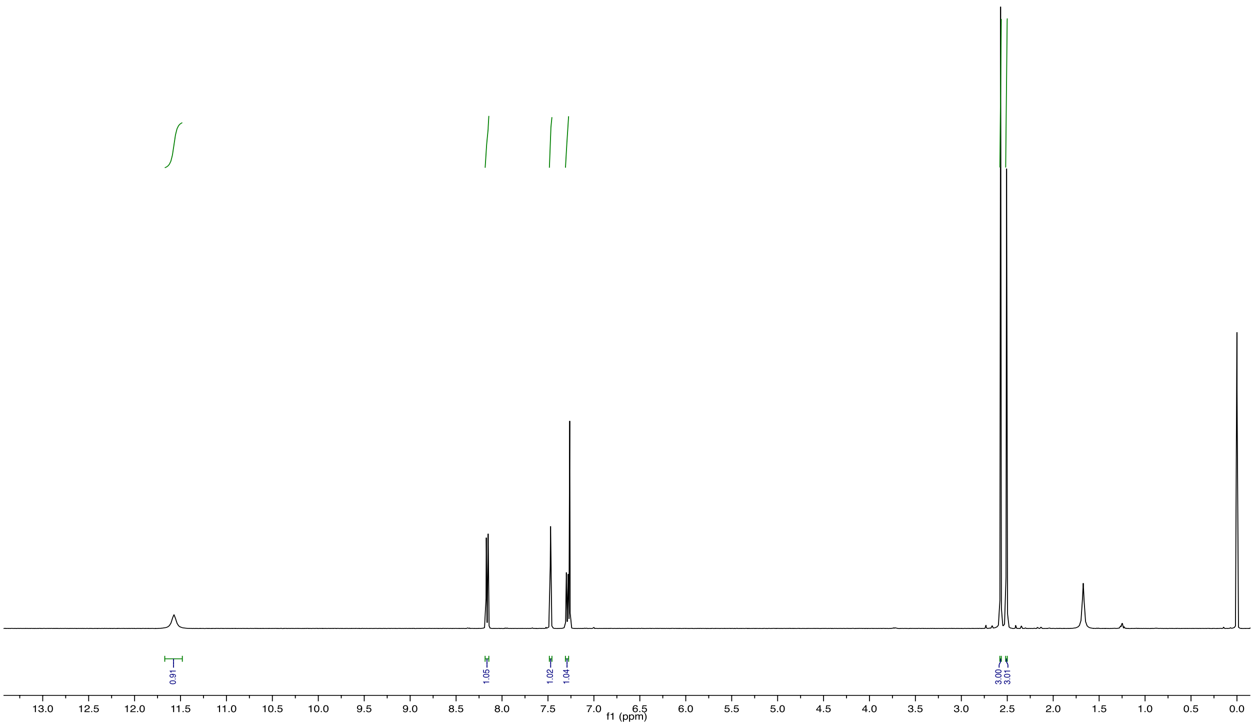

[13]C Spectrum of **8a**

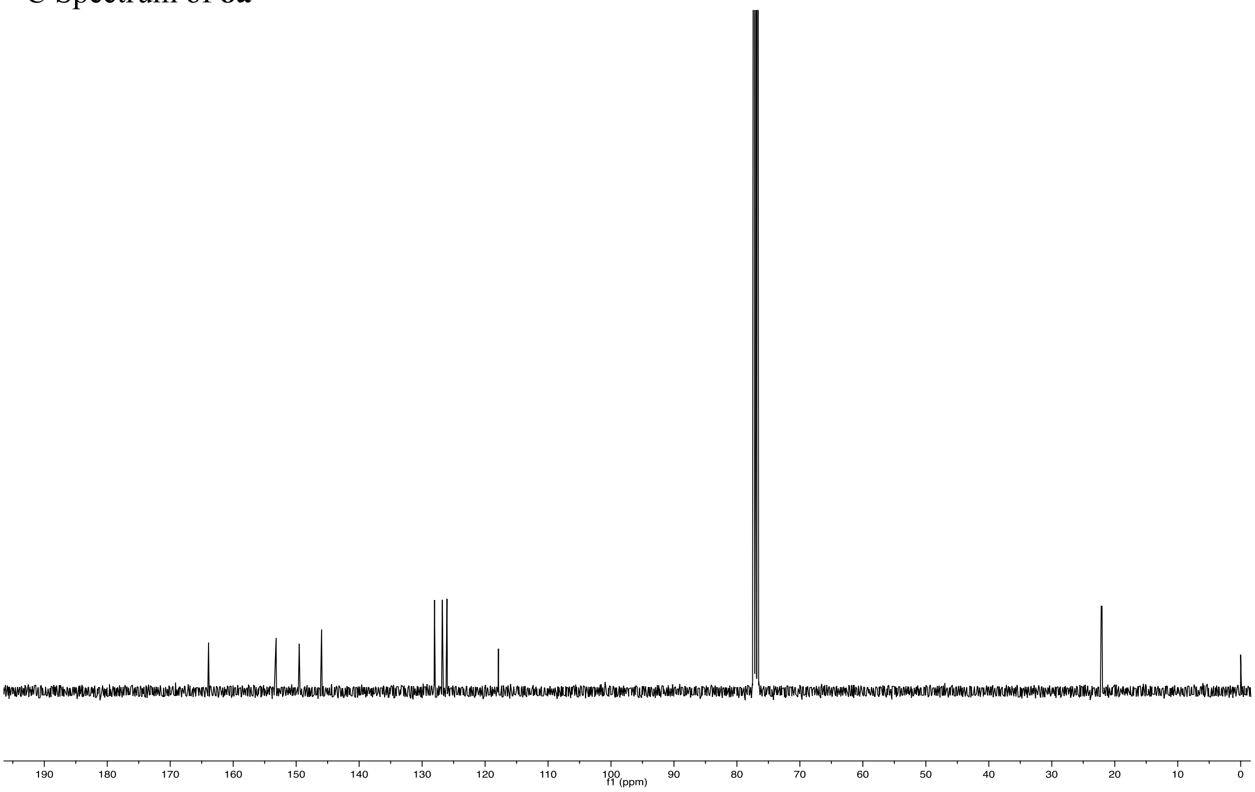

^{1}H Spectrum of 2-Ethyl-7-methylquinazolin-4(3*H*)-one (**8b**)

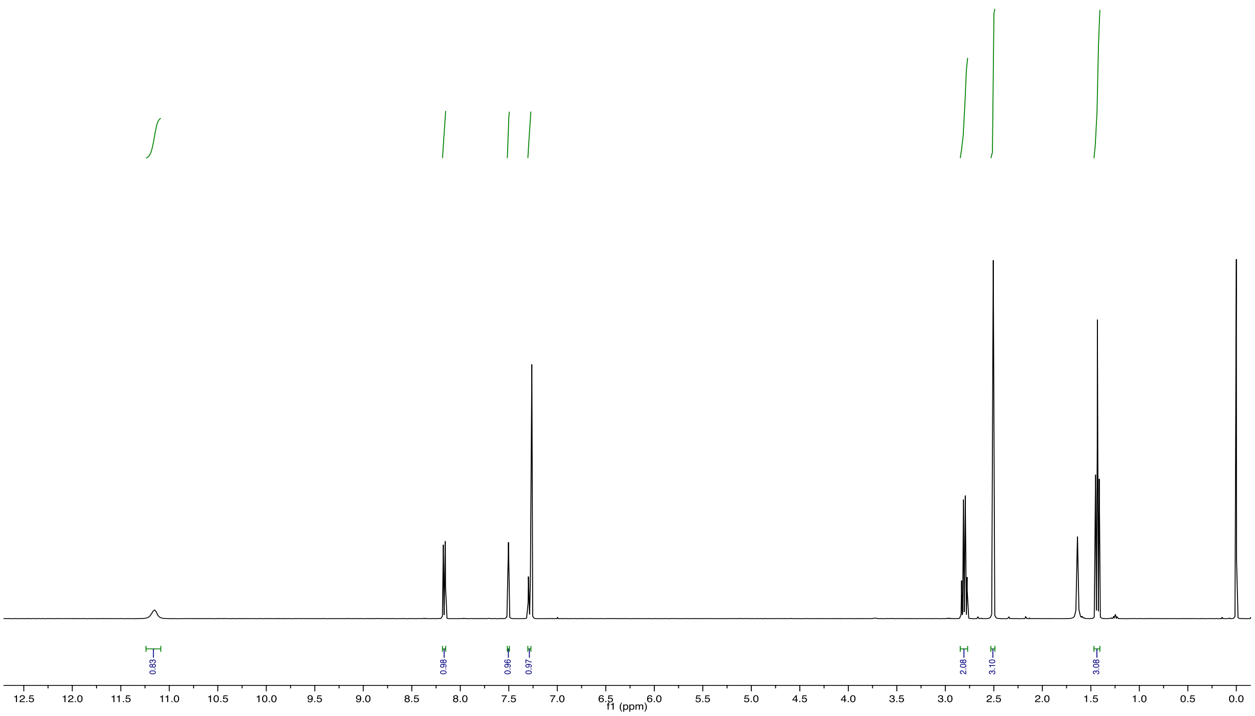

^{13}C Spectrum of **8b**

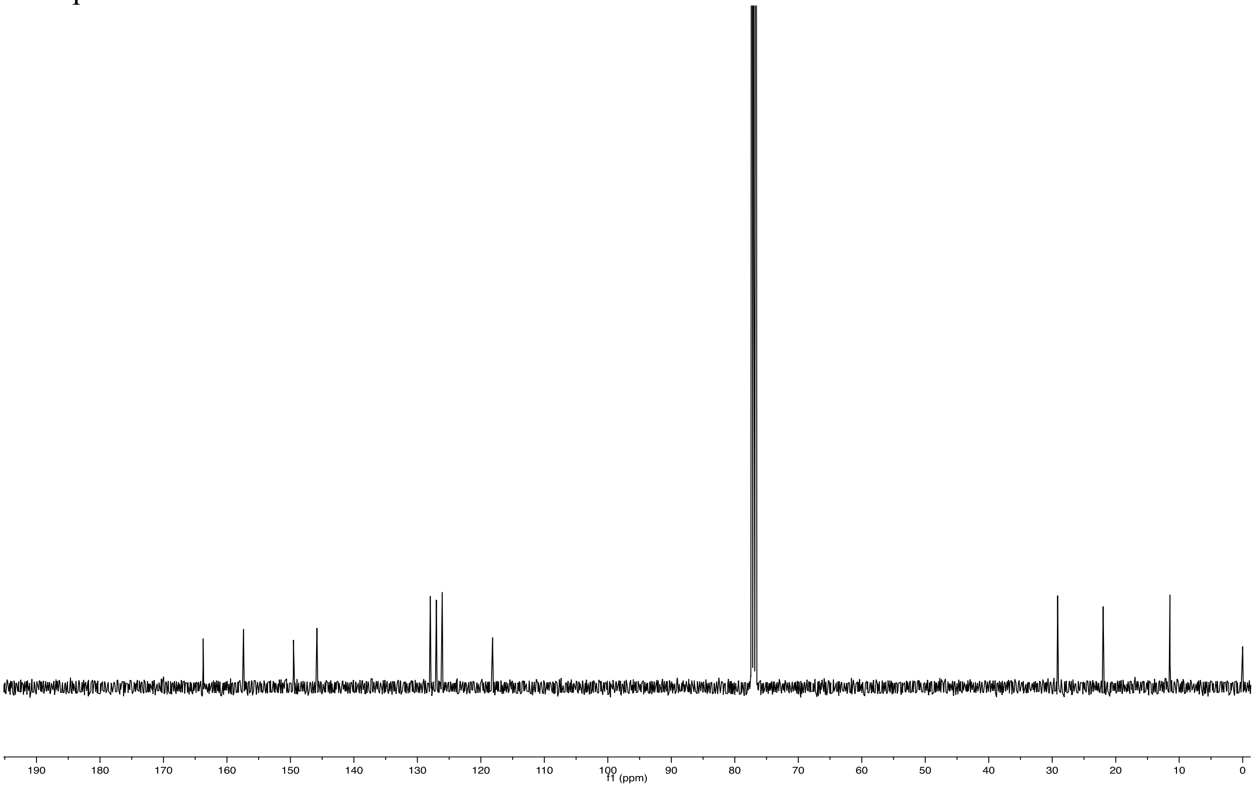

^{1}H Spectrum of 7-Methyl-2-phenylquinazolin-4(3*H*)-one (**8d**)

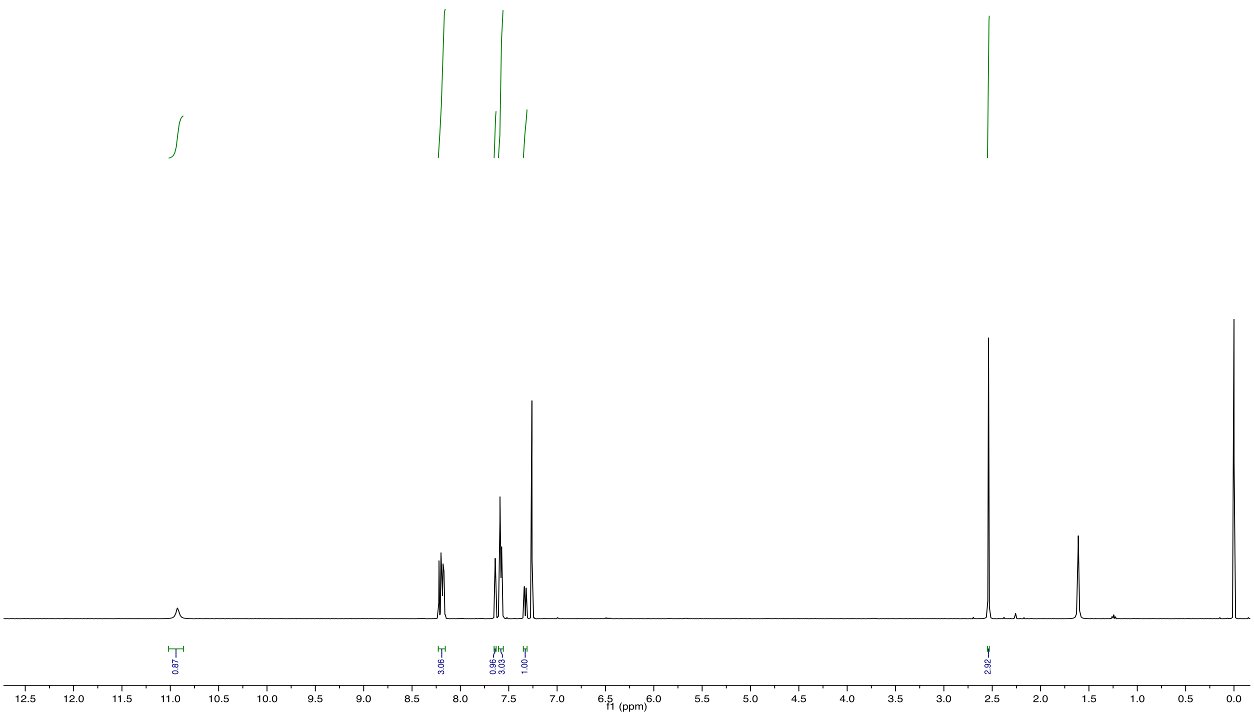

^{13}C Spectrum of **8d**

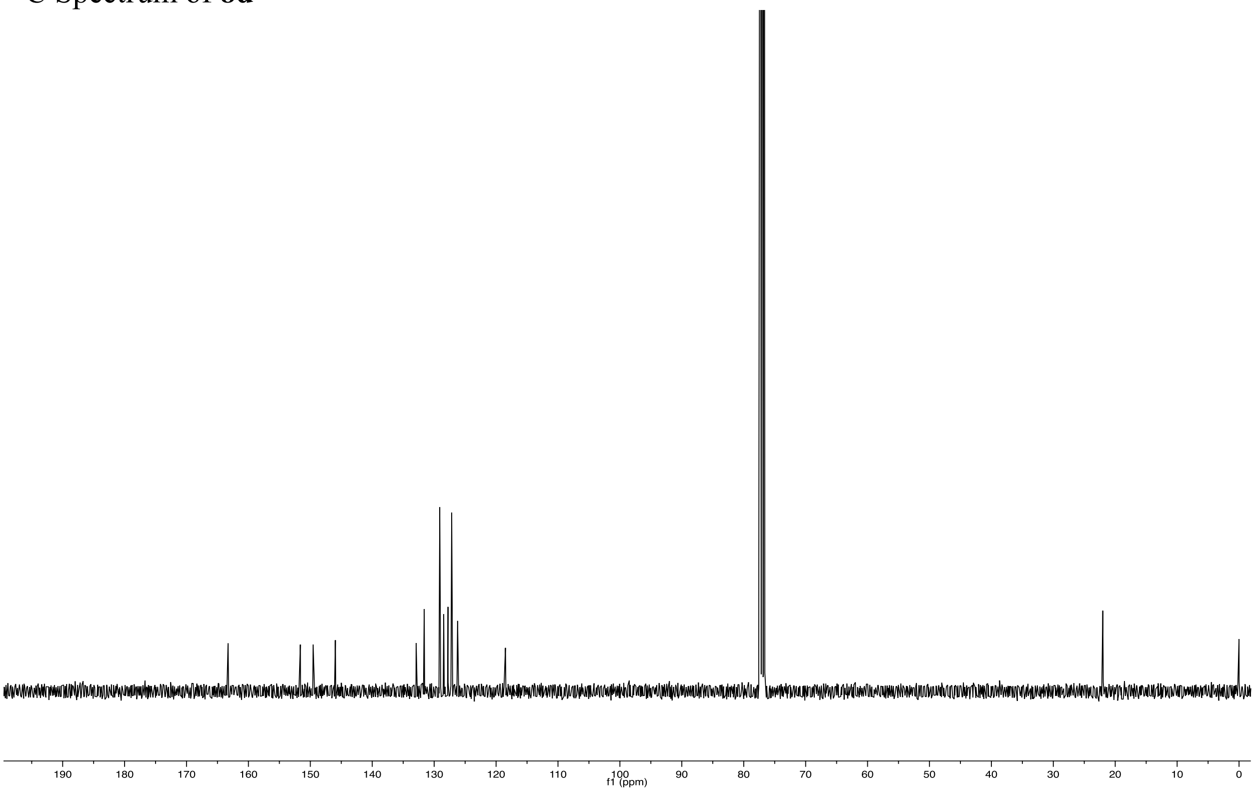

^{1}H Spectrum of 6-Chloro-2-methylquinazolin-4(3*H*)-one (**10a**)

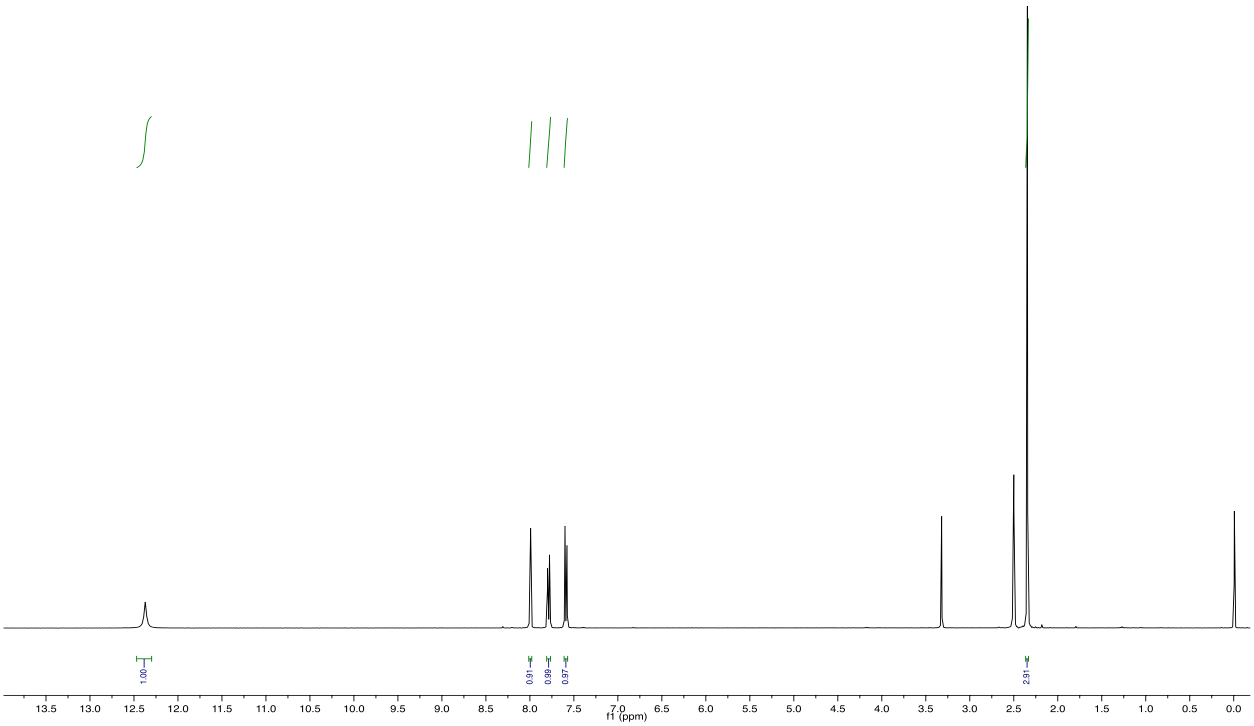

^{13}C Spectrum of **10a**

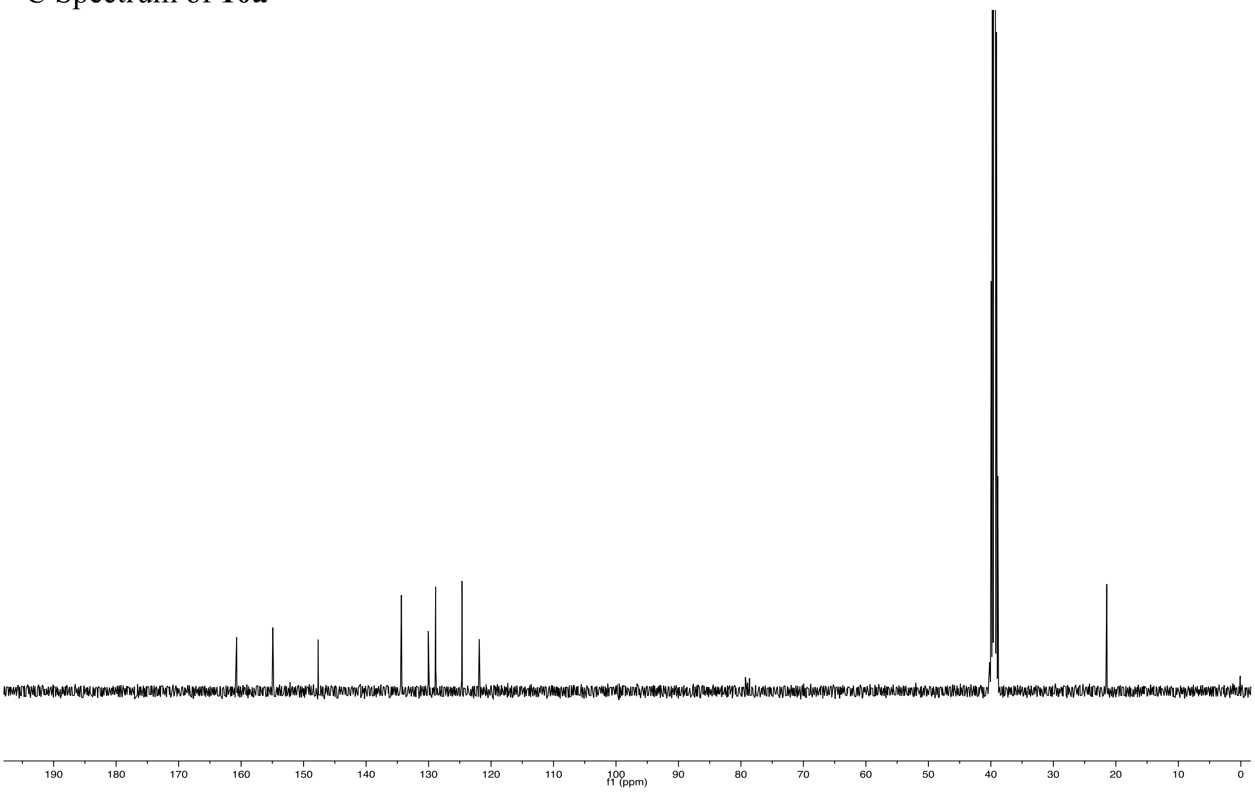

[1]H Spectra of 6-Chloro-2-ethylquinazolin-4(3*H*)-one (**10b**)

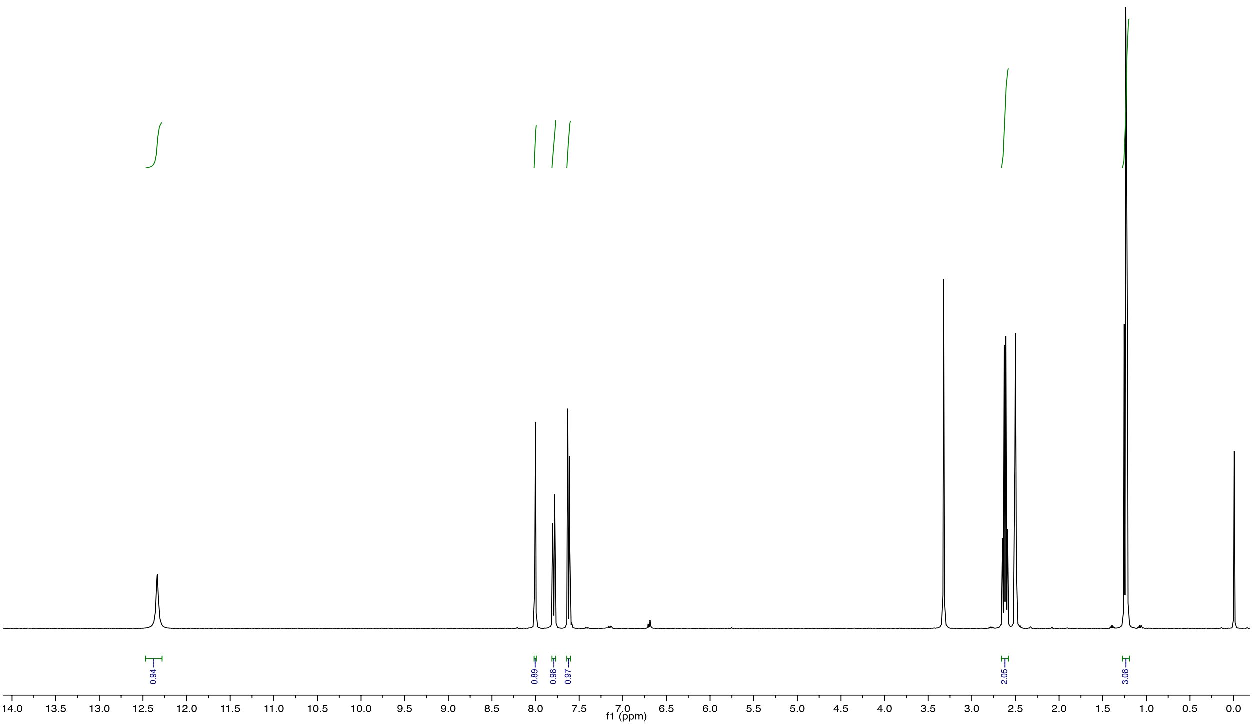

[13]C Spectrum of **10b**

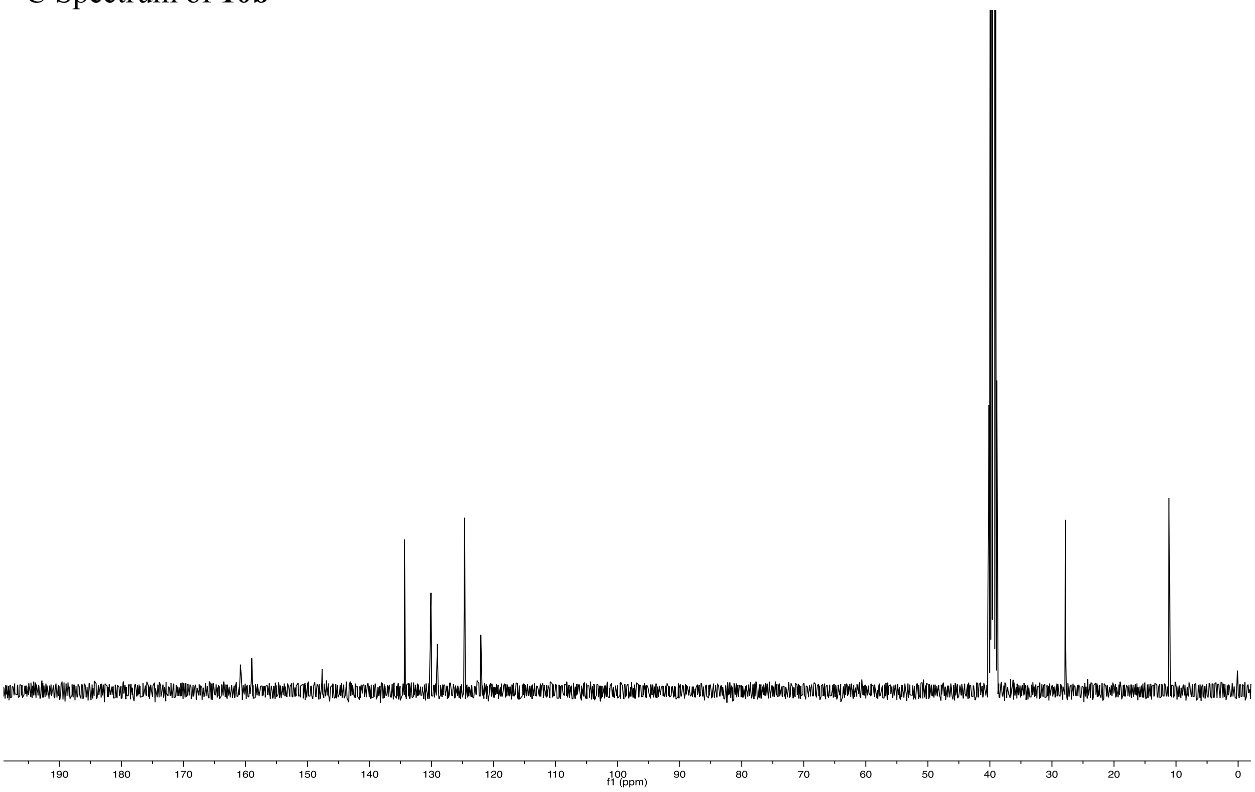

^{1}H Spectrum of 6-Chloro-2-propylquinazolin-4(3*H*)-one (**10c**)

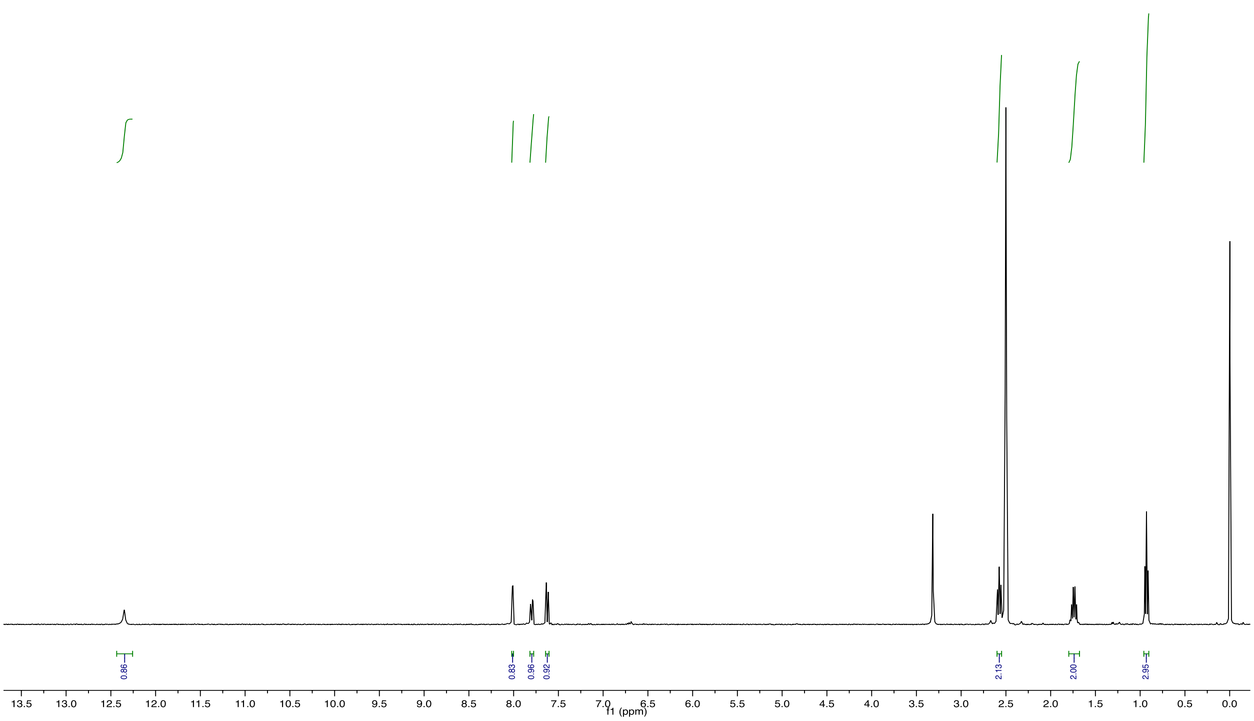

^{13}C Spectrum of **10c**

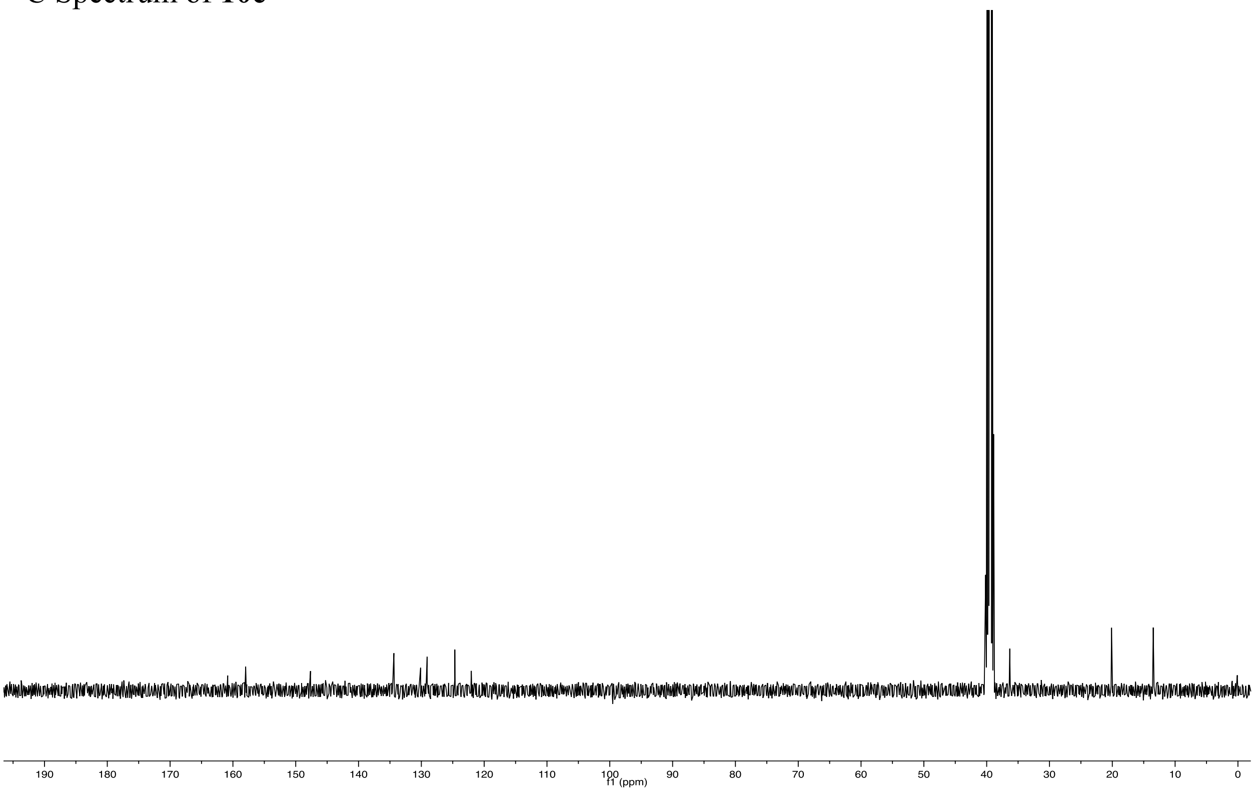

[1]H Spectrum of 6-Chloro-2-phenylquinazolin-4(3*H*)-one (**10d**)

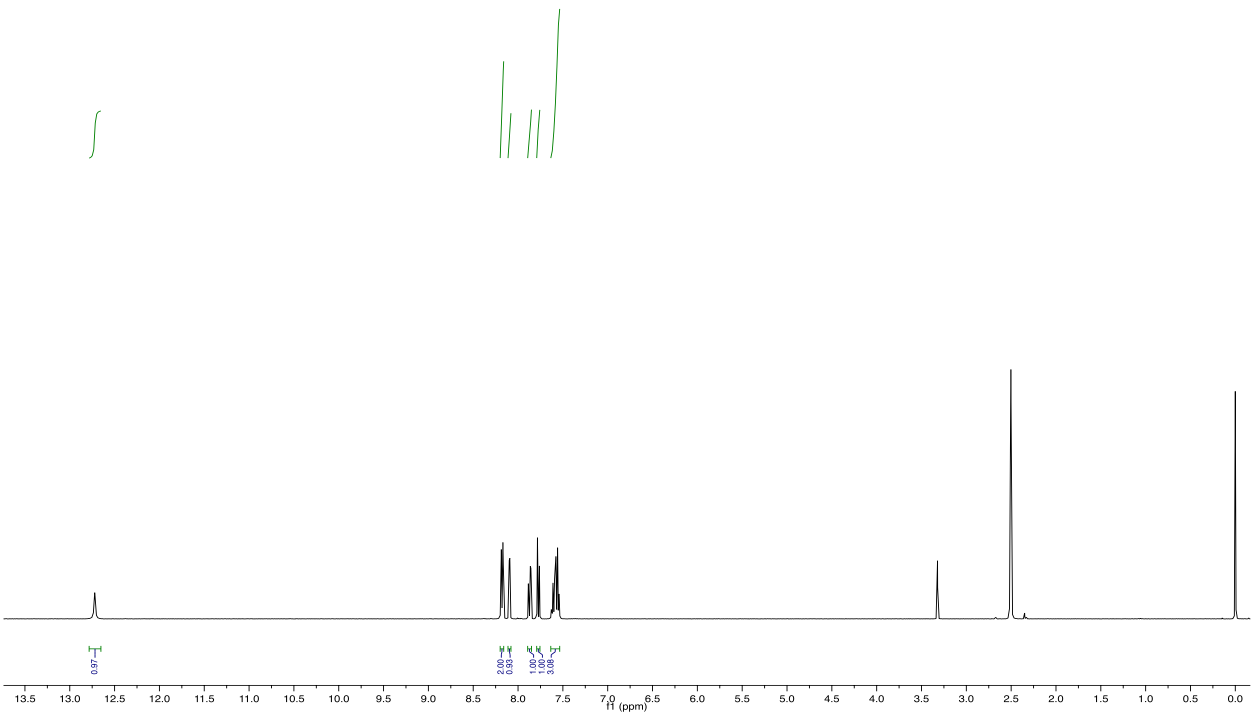

[13]C Spectrum of **10d**

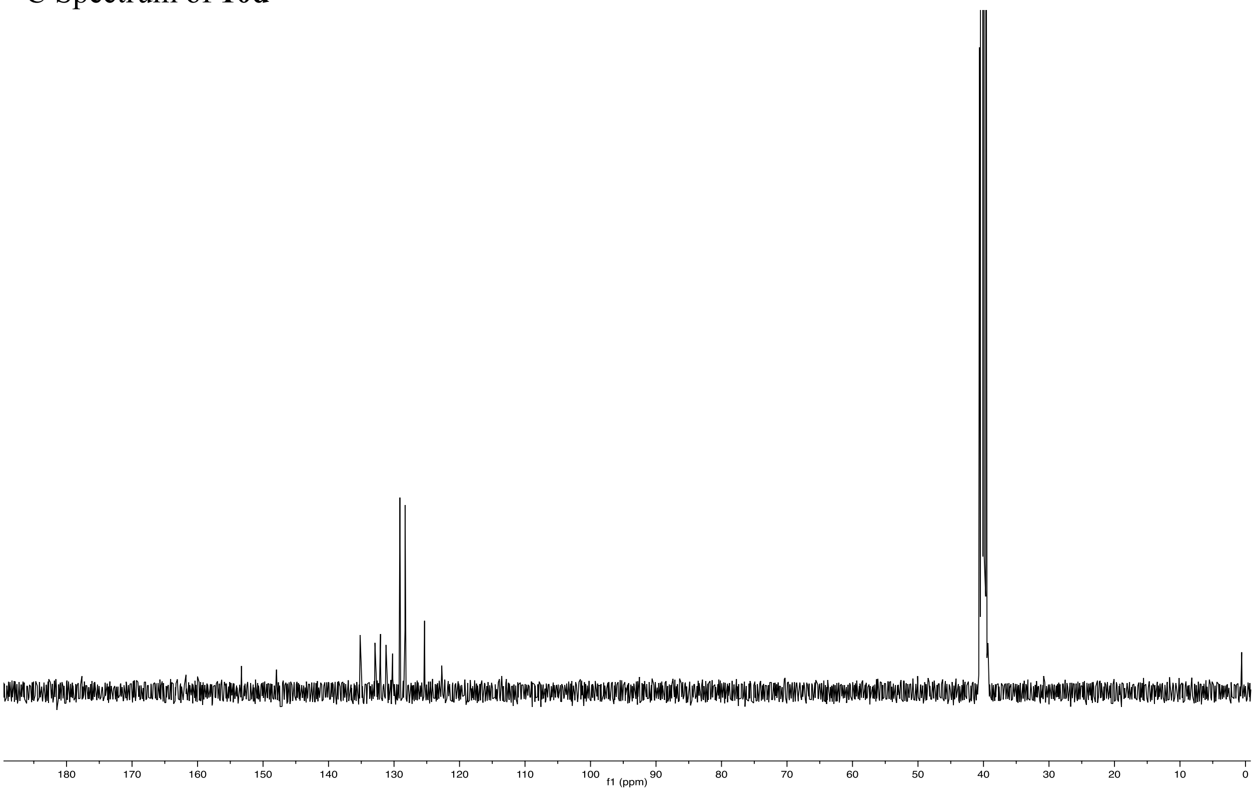

^{1}H Spectrum of 7-Chloro-2-methylquinazolin-4(3*H*)-one (**12a**)

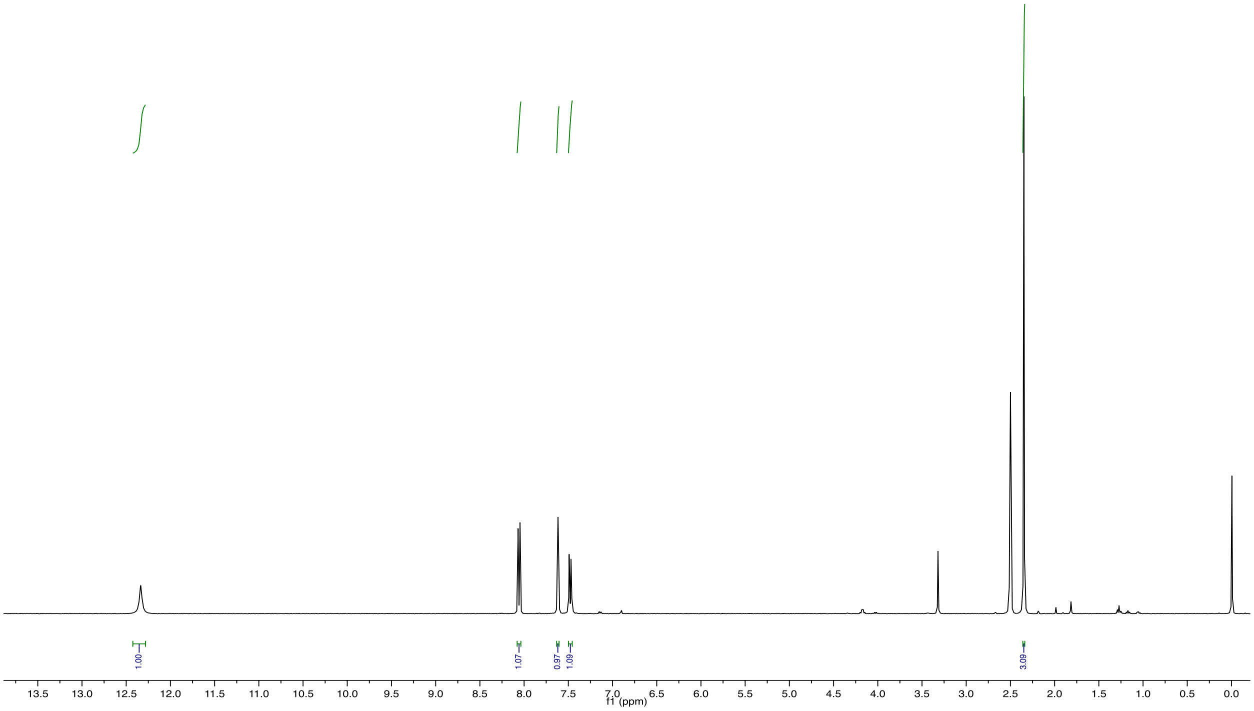

^{13}C Spectrum of **12a**

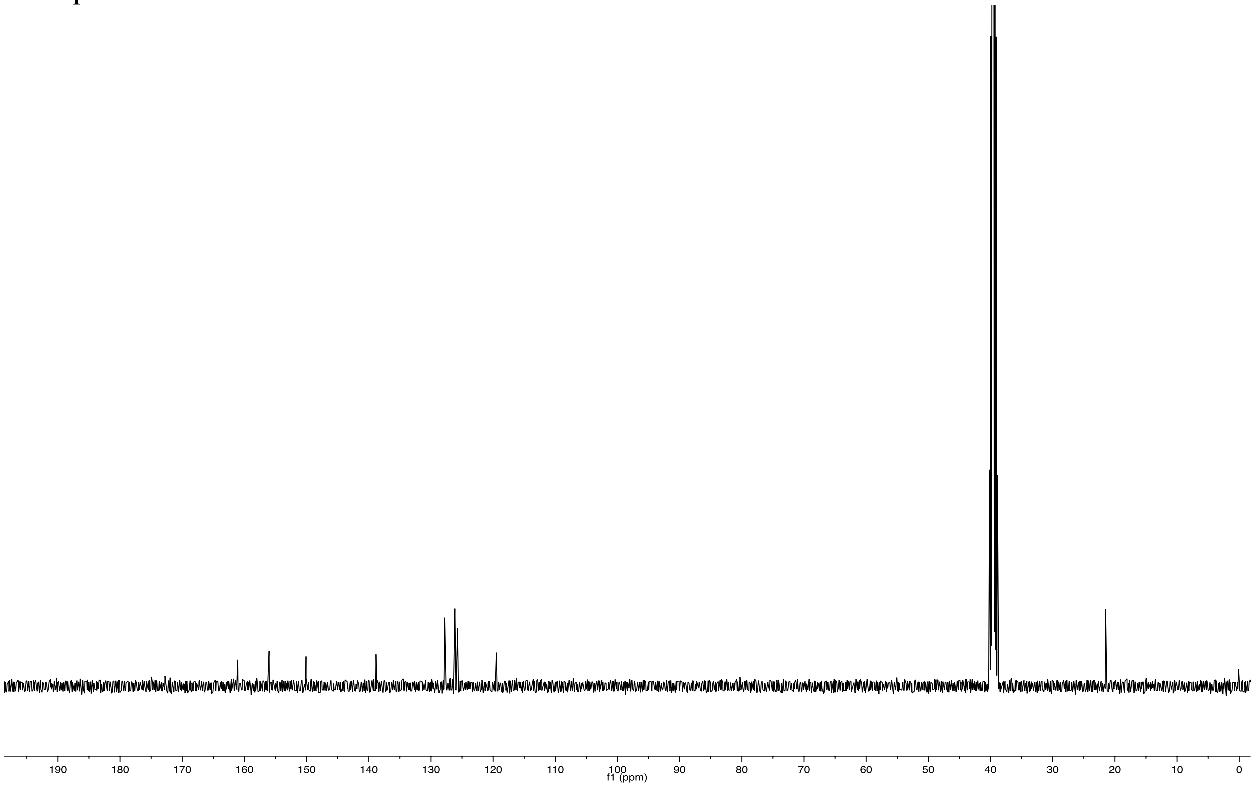

^{1}H Spectrum of 7-Chloro-2-ethylquinazolin-4(3*H*)-one (**12b**)

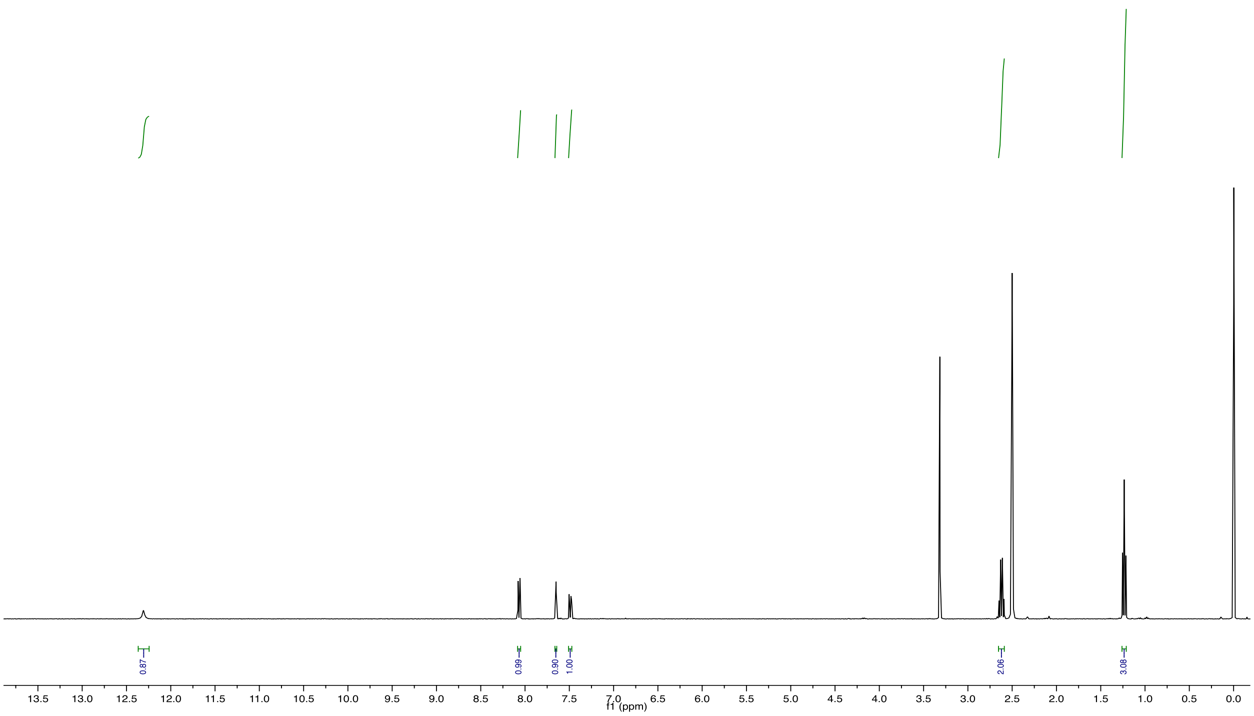

^{13}C Spectrum of **12b**

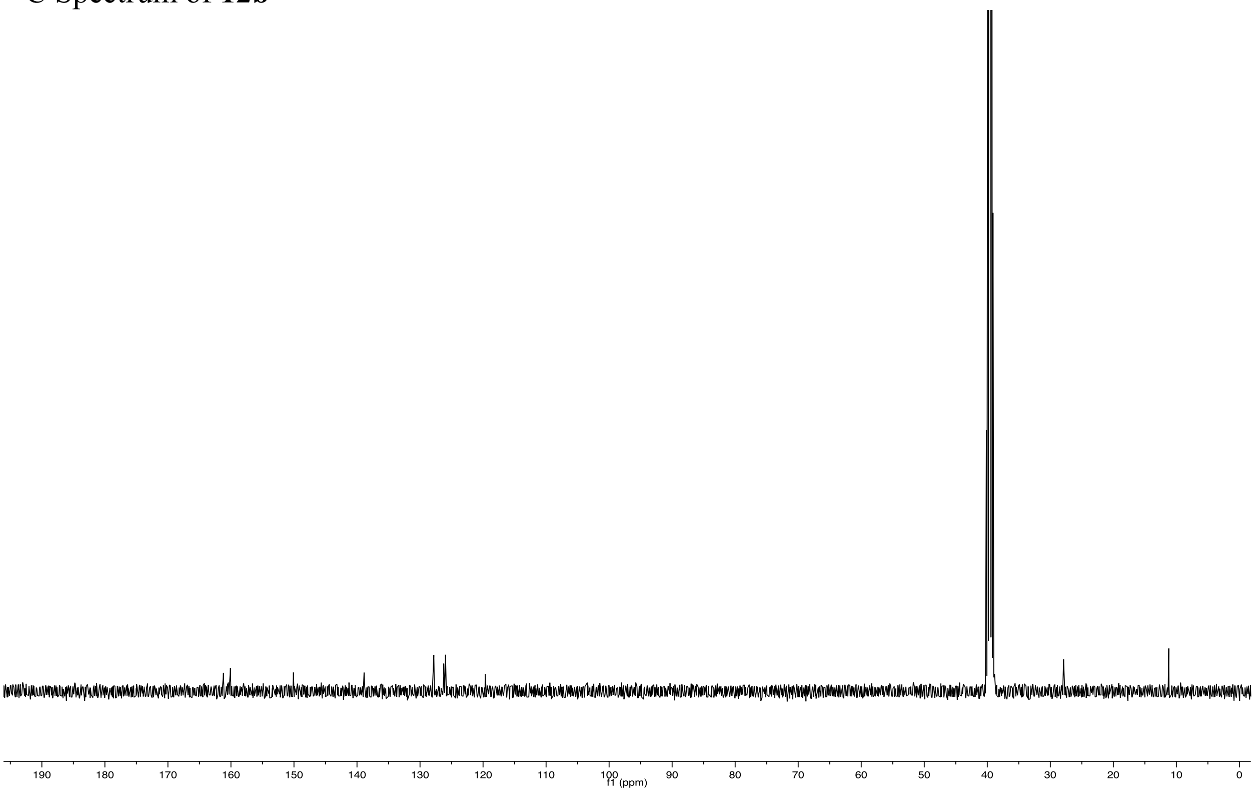

^{1}H Spectrum of 7-Chloro-2-propylquinazolin-4(3*H*)-one (**12c**)

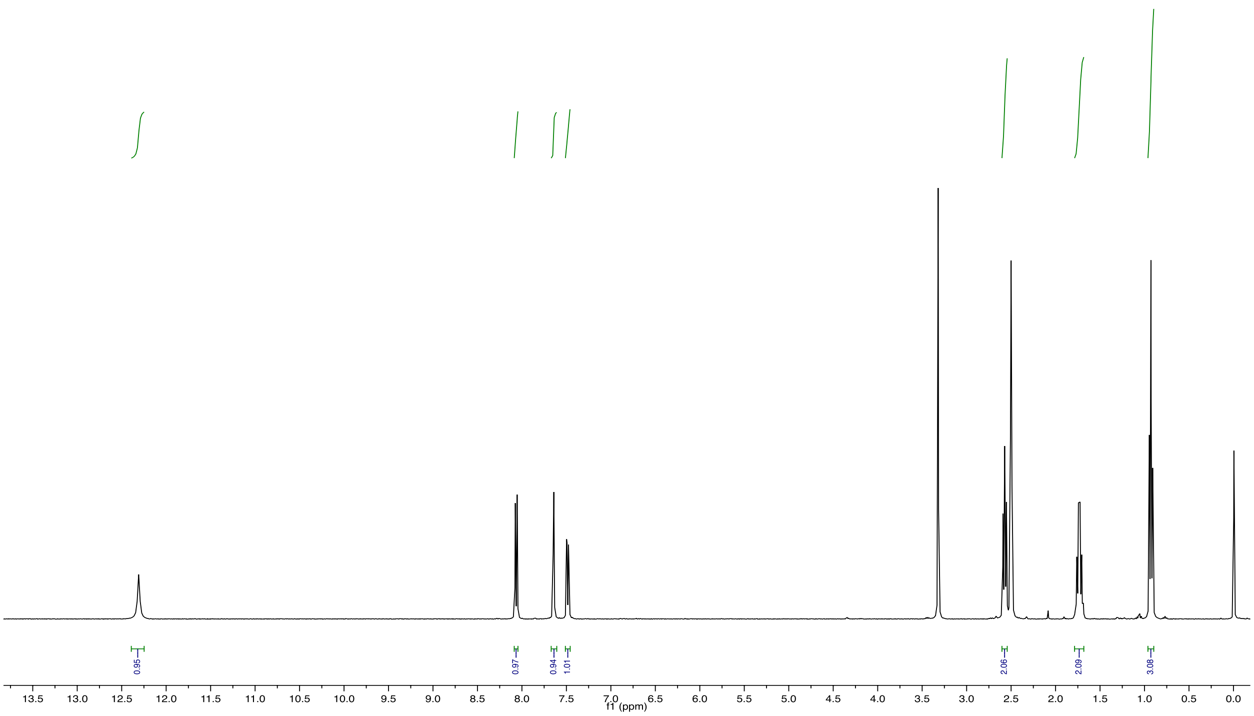

^{13}C Spectrum of **12c**

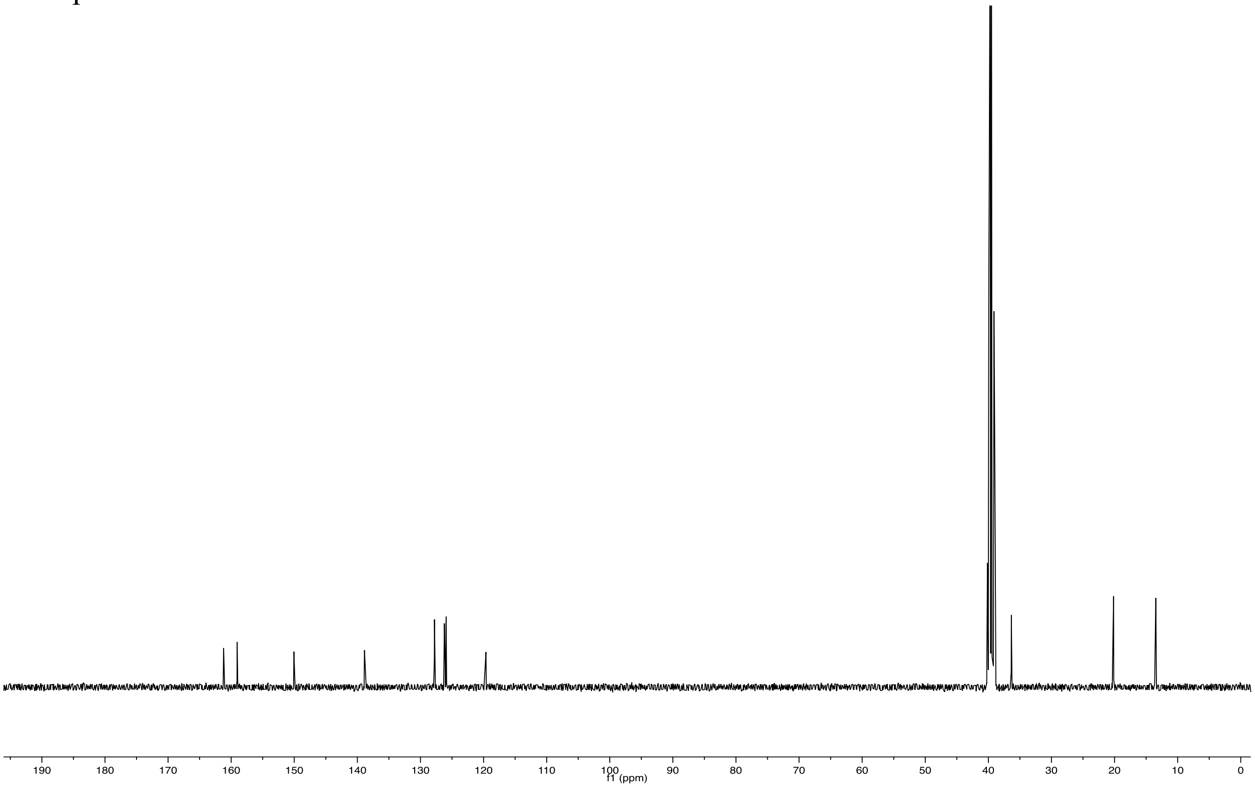

^{1}H Spectrum of 7-Chloro-2-phenylquinazolin-4(3*H*)-one (**12d**)

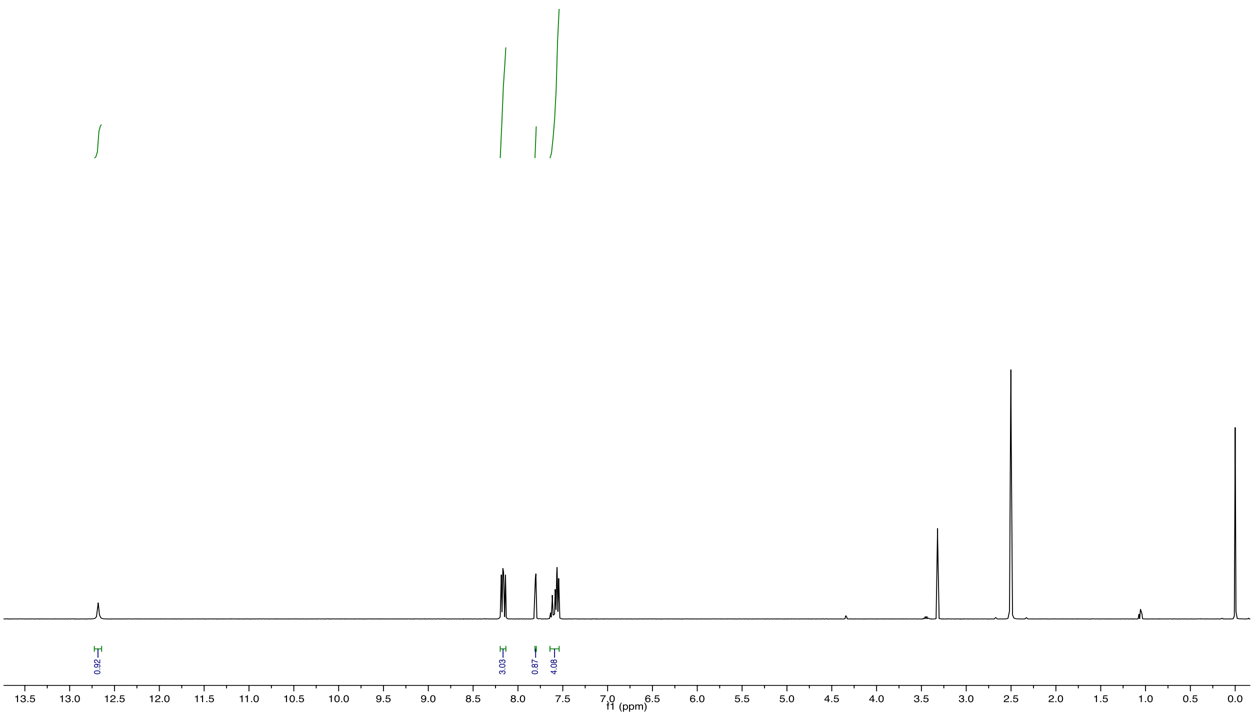

^{13}C Spectrum of **12d**

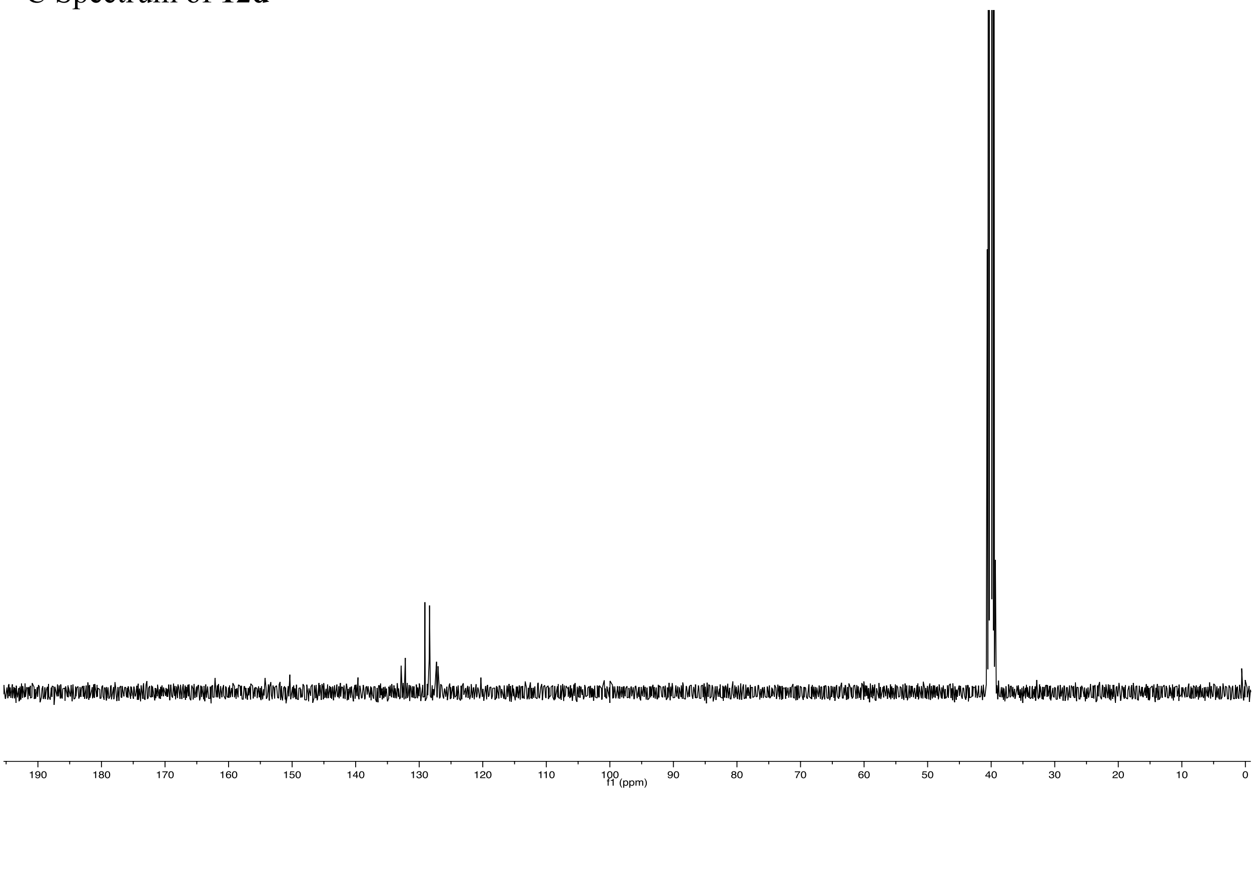

[1]H Spectrum of 2-Methylpyrido[2,3-*d*]pyrimidin-4(3*H*)-one (**14a**)

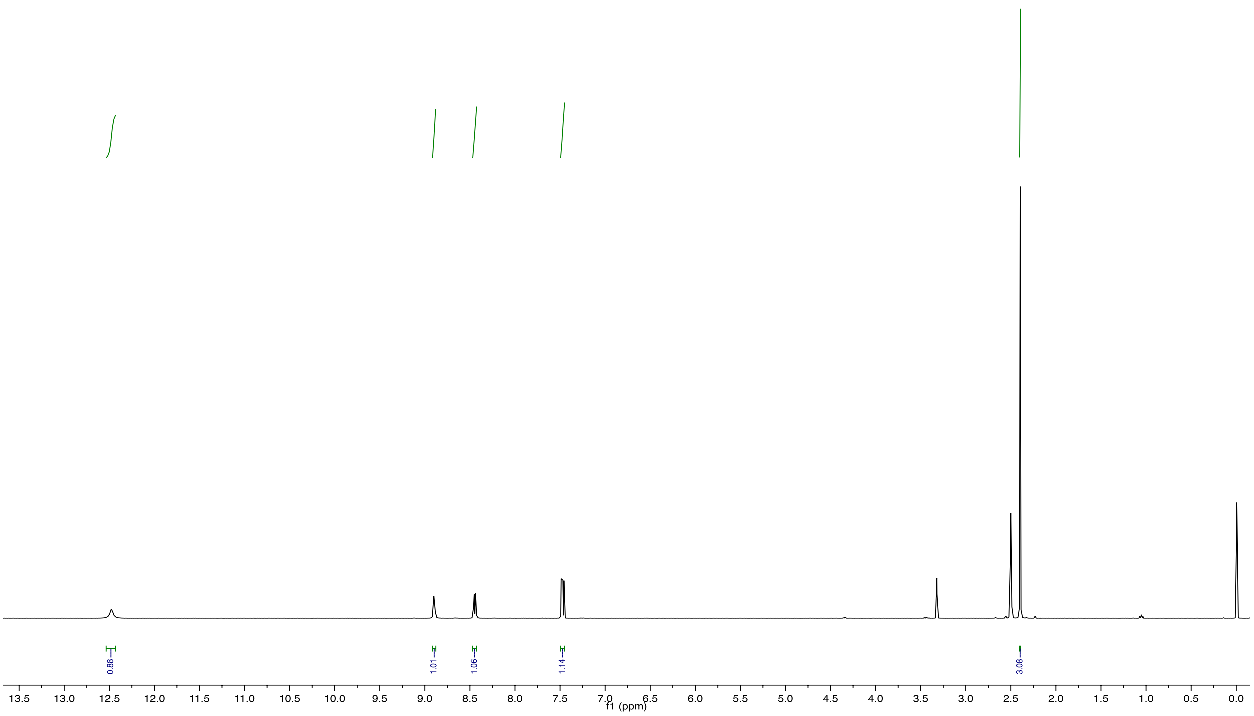

[13]C Spectrum of **14a**

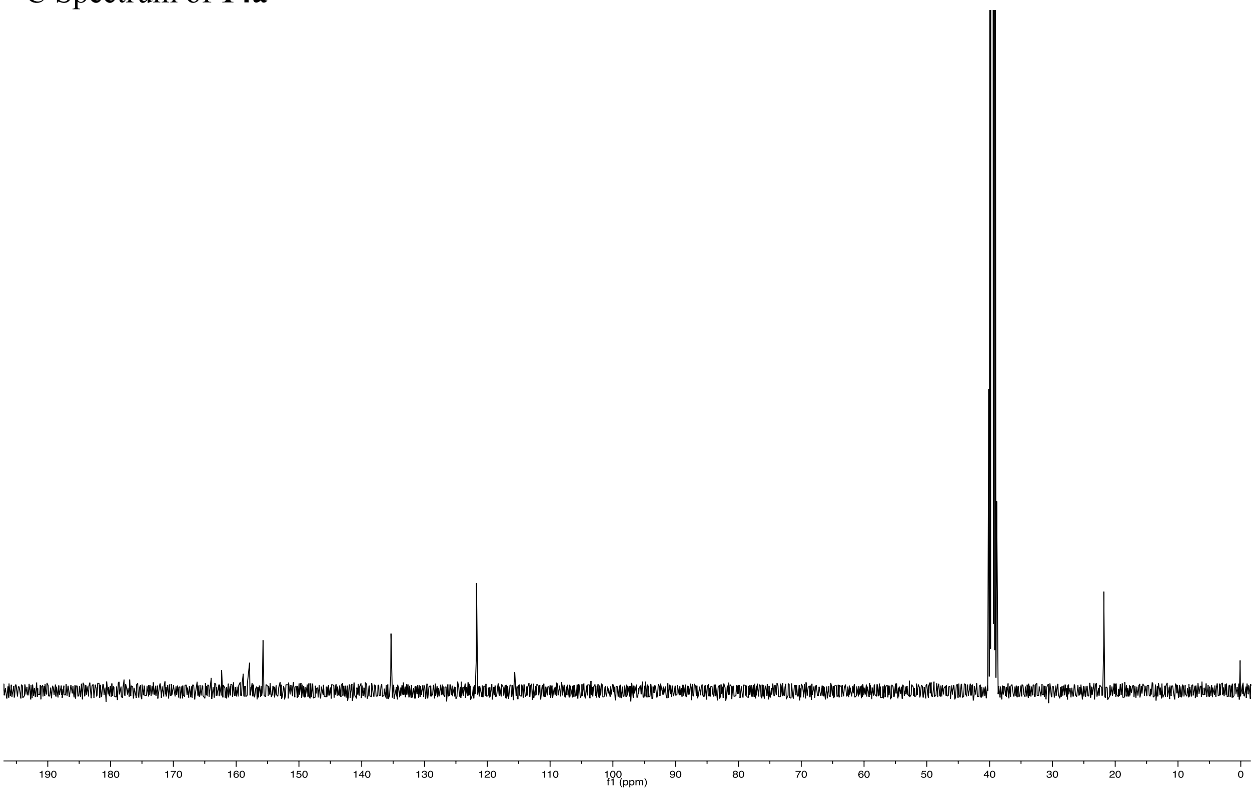

^{1}H Spectrum of 2-Ethylpyrido[2,3-*d*]pyrimidin-4(3*H*)-one (**14b**)

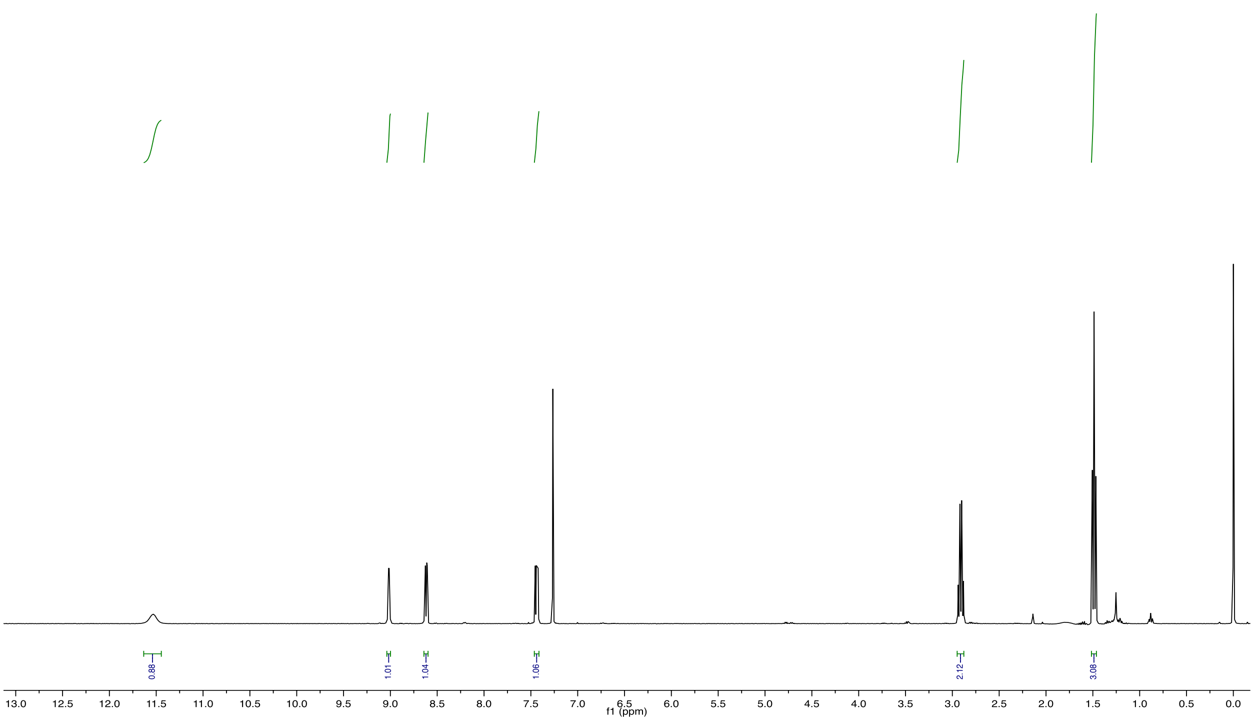

^{13}C Spectrum of **14b**

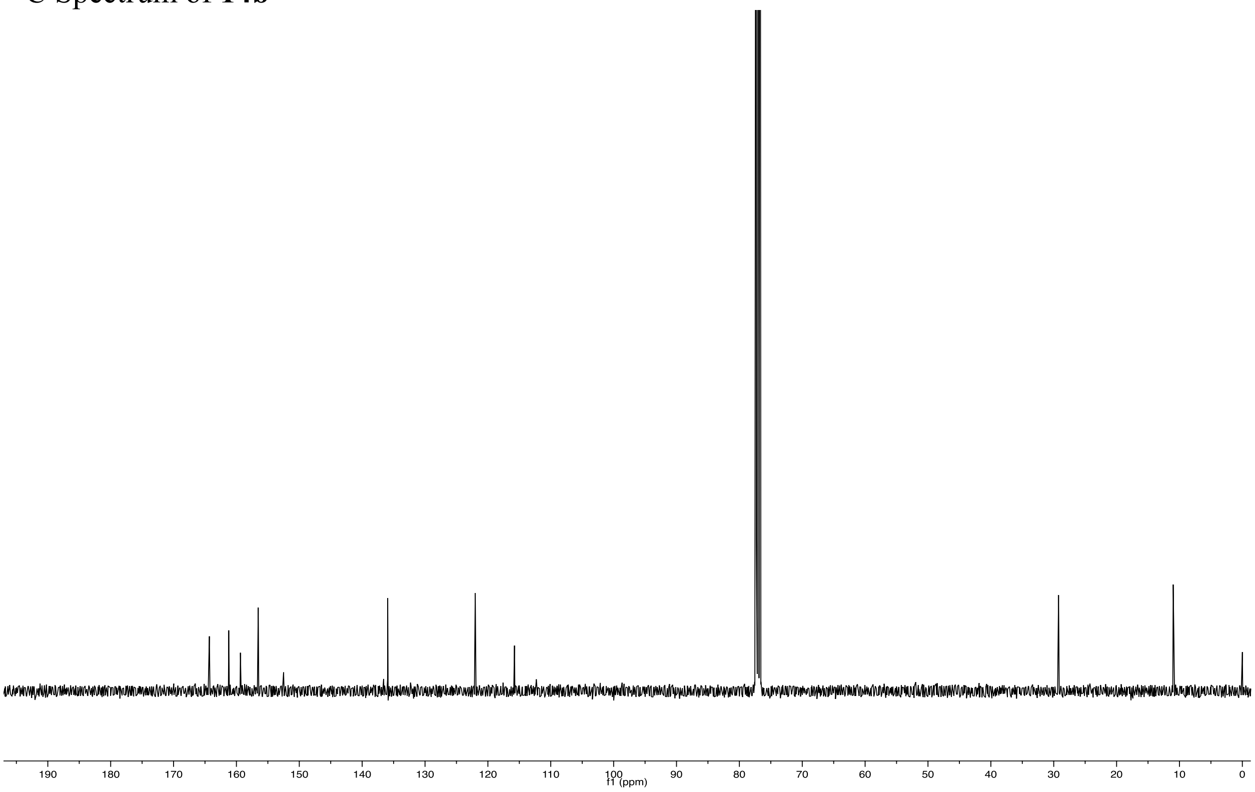

[1]H Spectrum of 2-Propylpyrido[2,3-*d*]pyrimidin-4(3*H*)-one (**14c**)

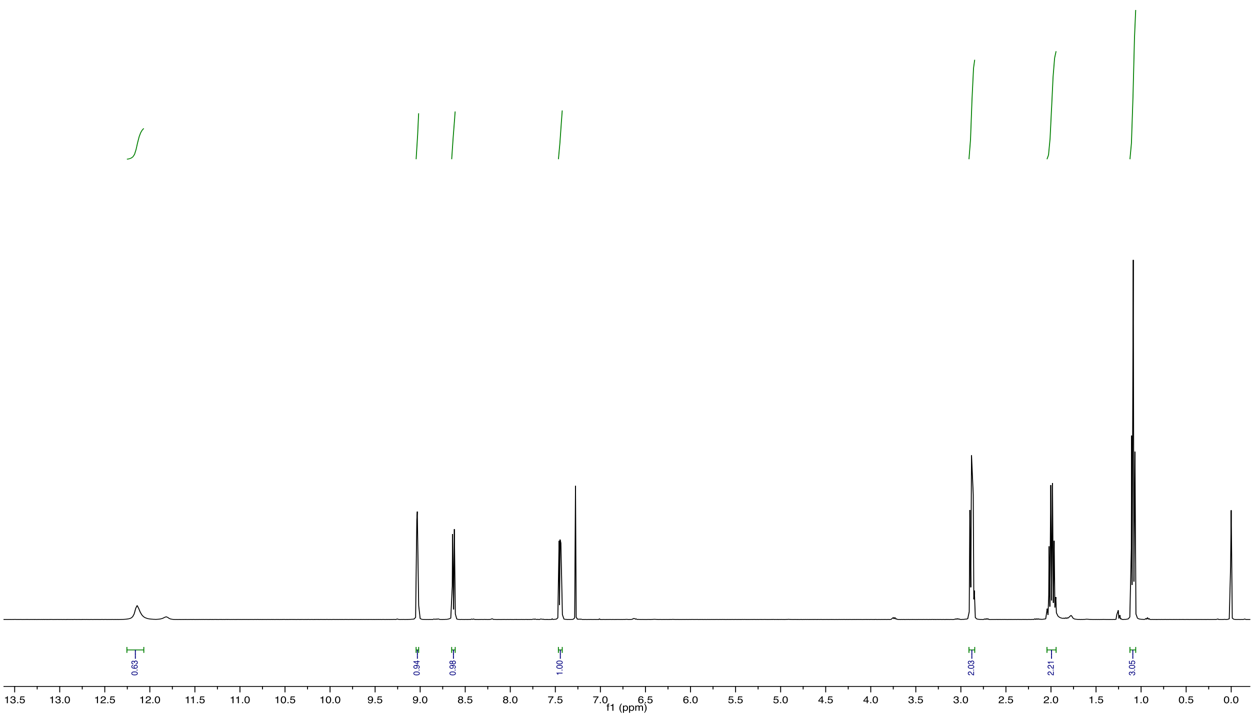

[13]C Spectrum of **14c**

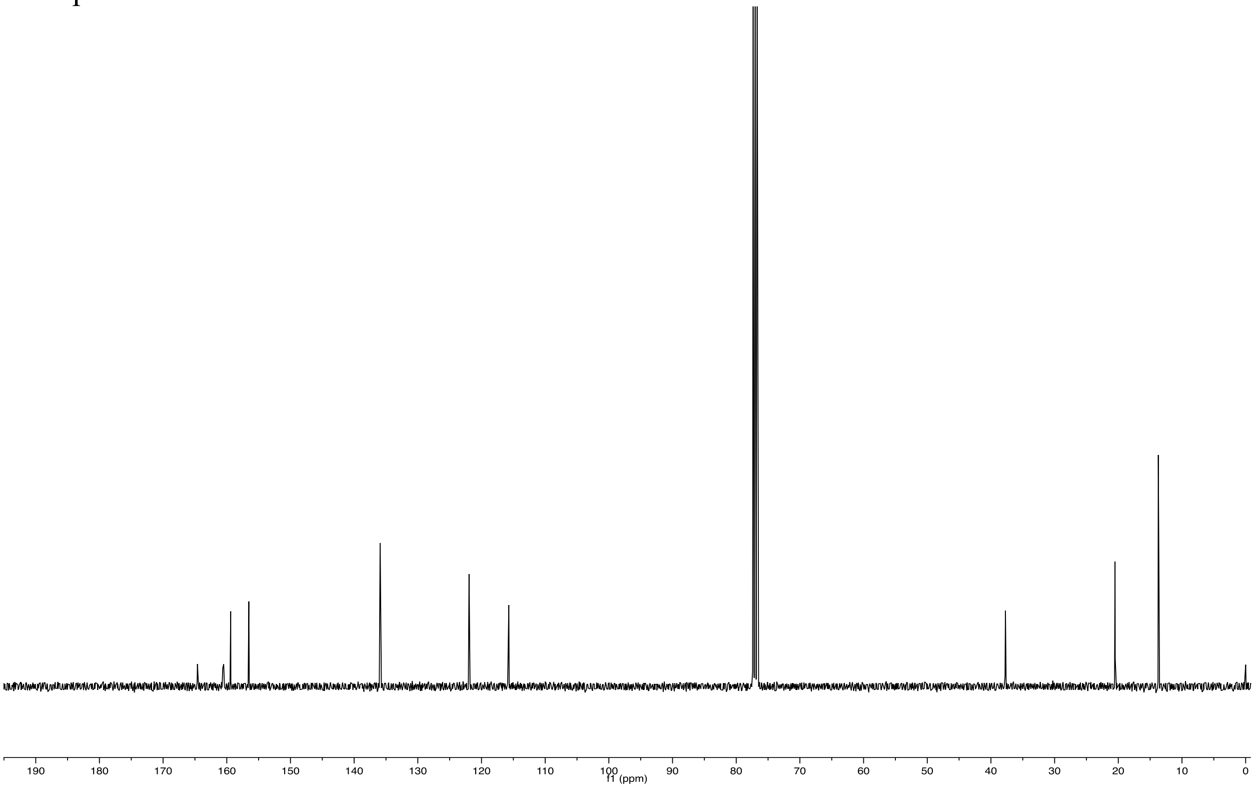

^{1}H Spectrum of 2-Phenylpyrido[2,3-*d*]pyrimidin-4(3*H*)-one (**14d**)

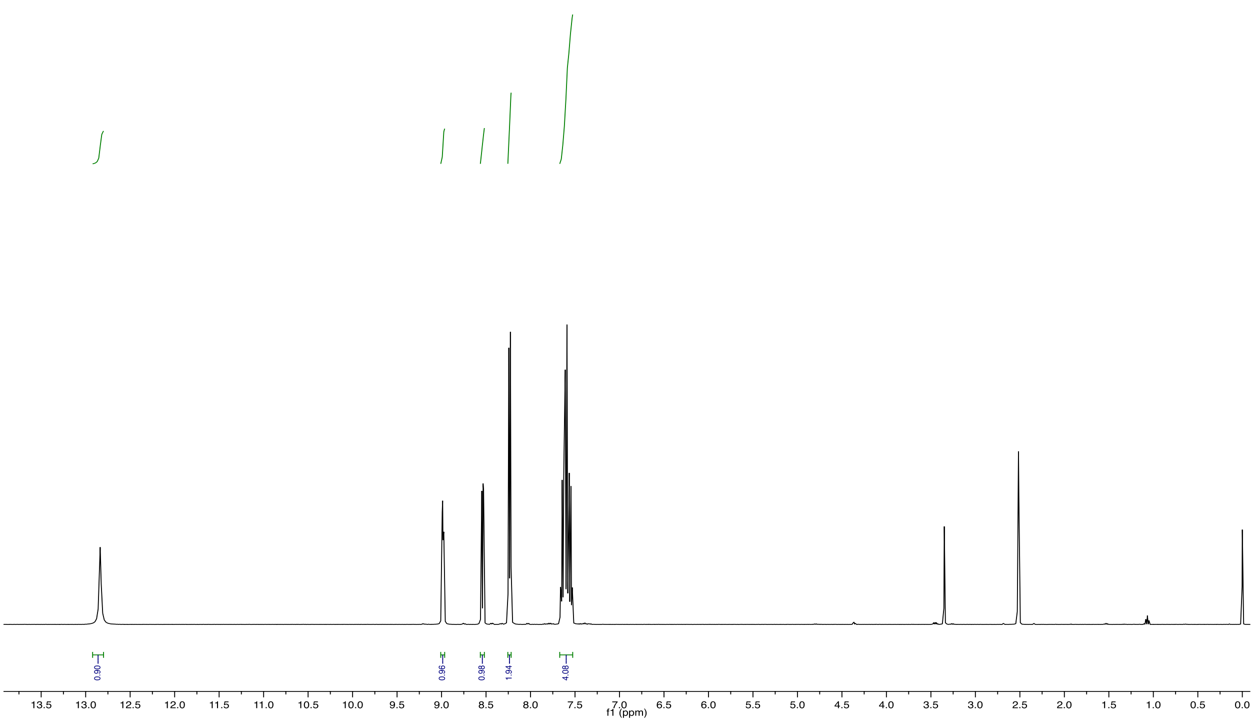

^{13}C Spectrum of **14d**

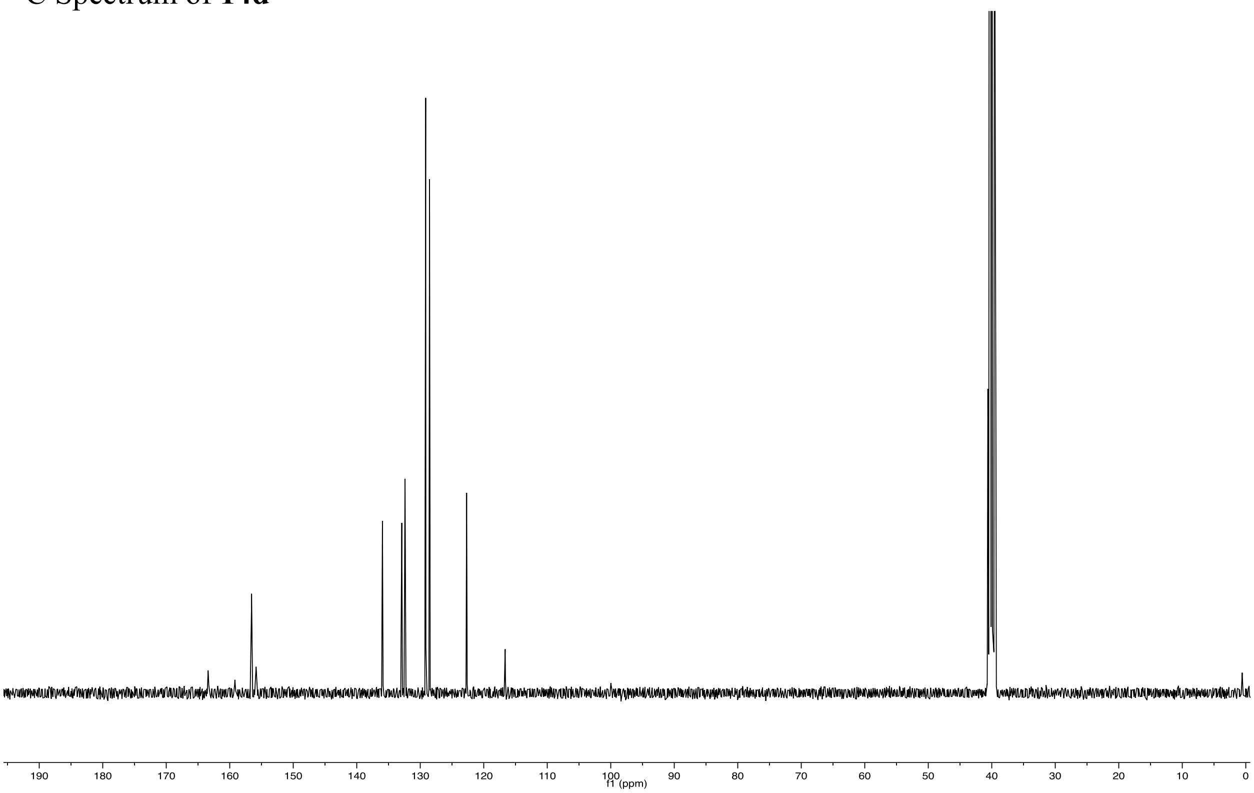

^{1}H Spectrum of 2,3-Dimethylquinazolin-4(3*H*)-one (**16a**)

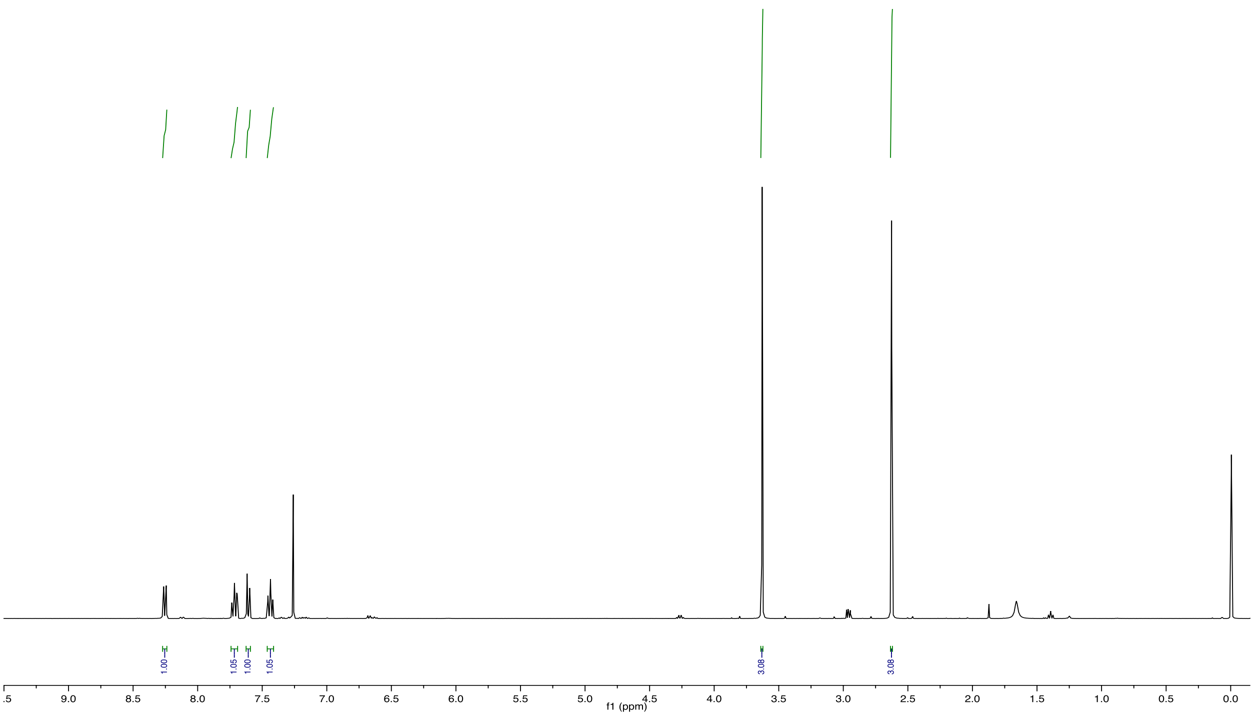

^{13}C Spectrum of **16a**

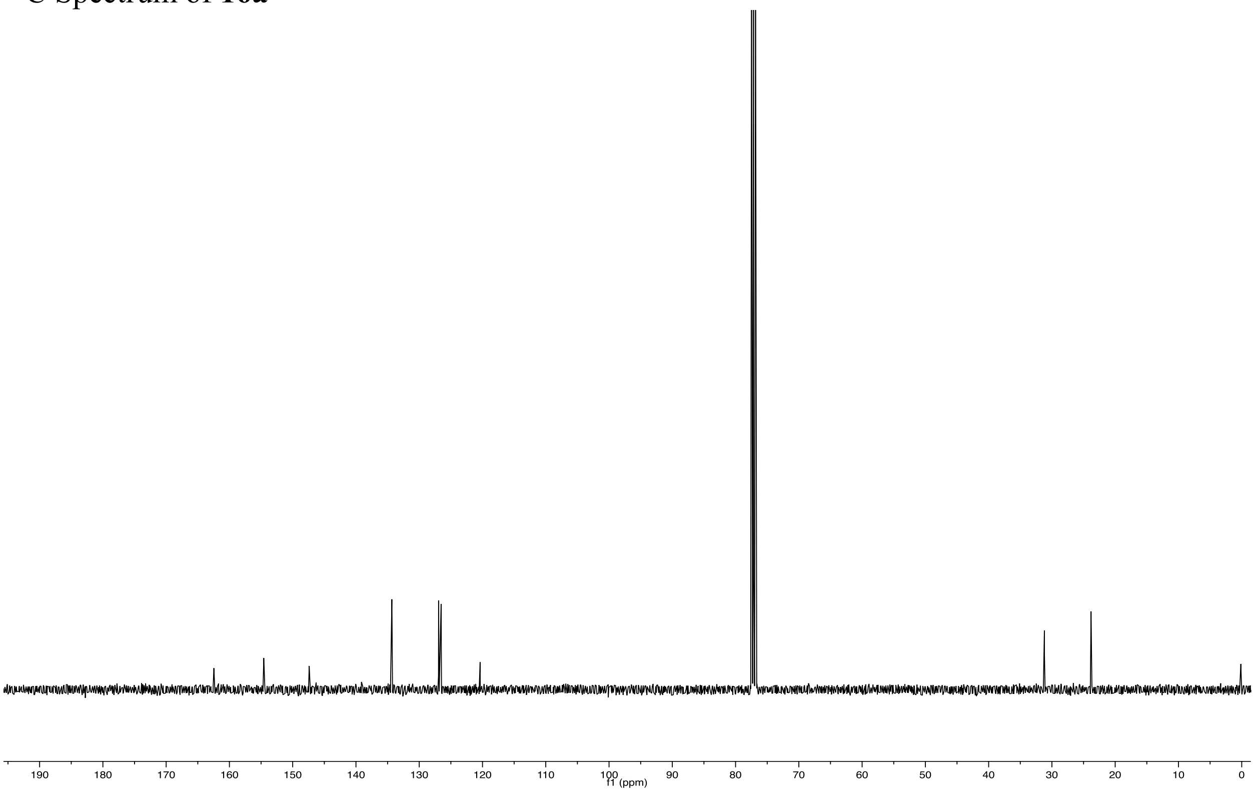

^{1}H Spectrum of 2-Ethyl-3-methylquinazolin-4(3*H*)-one (**16b**)

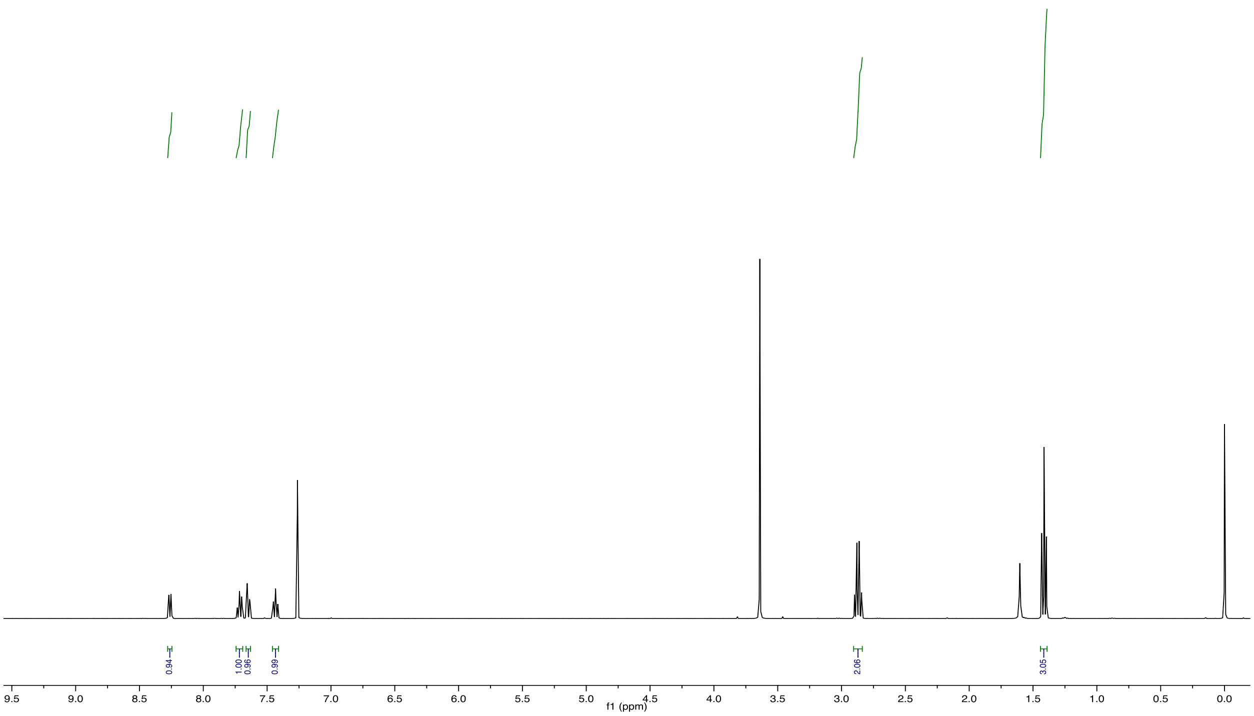

^{13}C Spectrum of **16b**

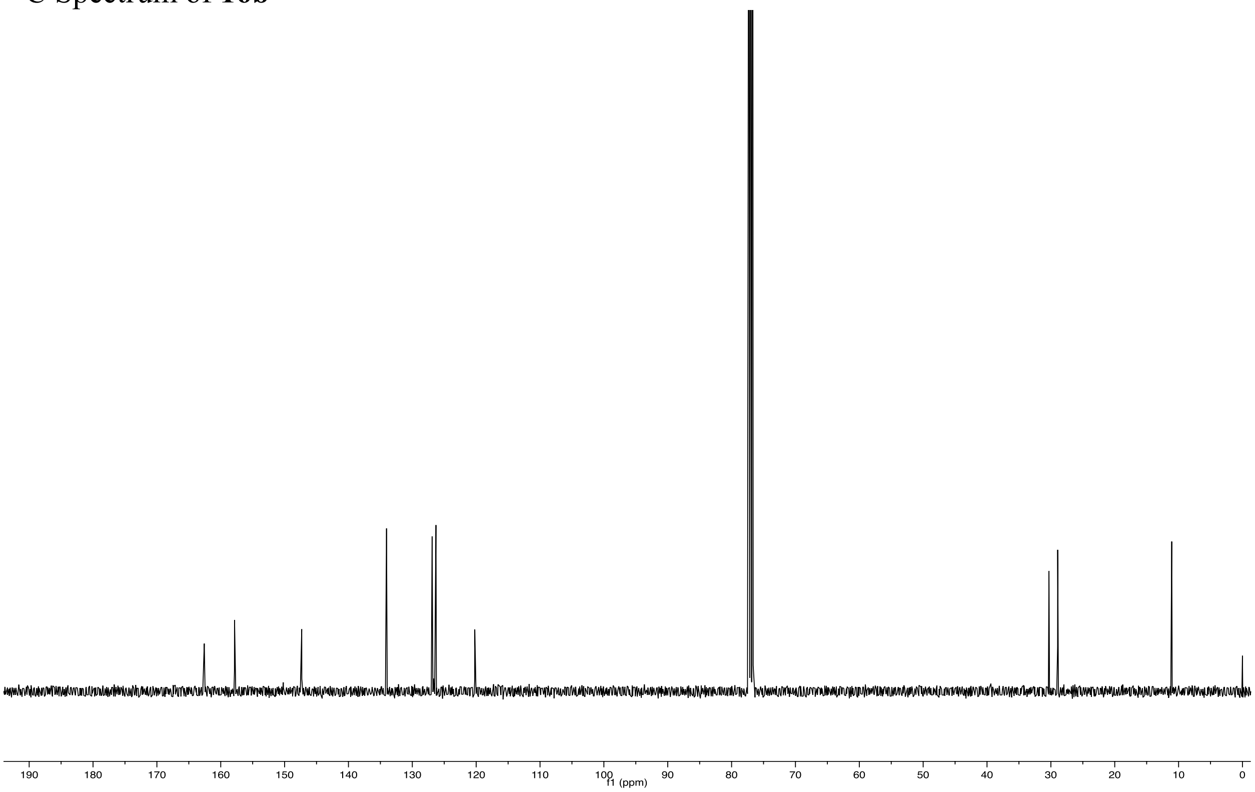

[1]H Spectrum of 2-Phenyl-3-methylquinazolin-4(3*H*)-one (**16d**)

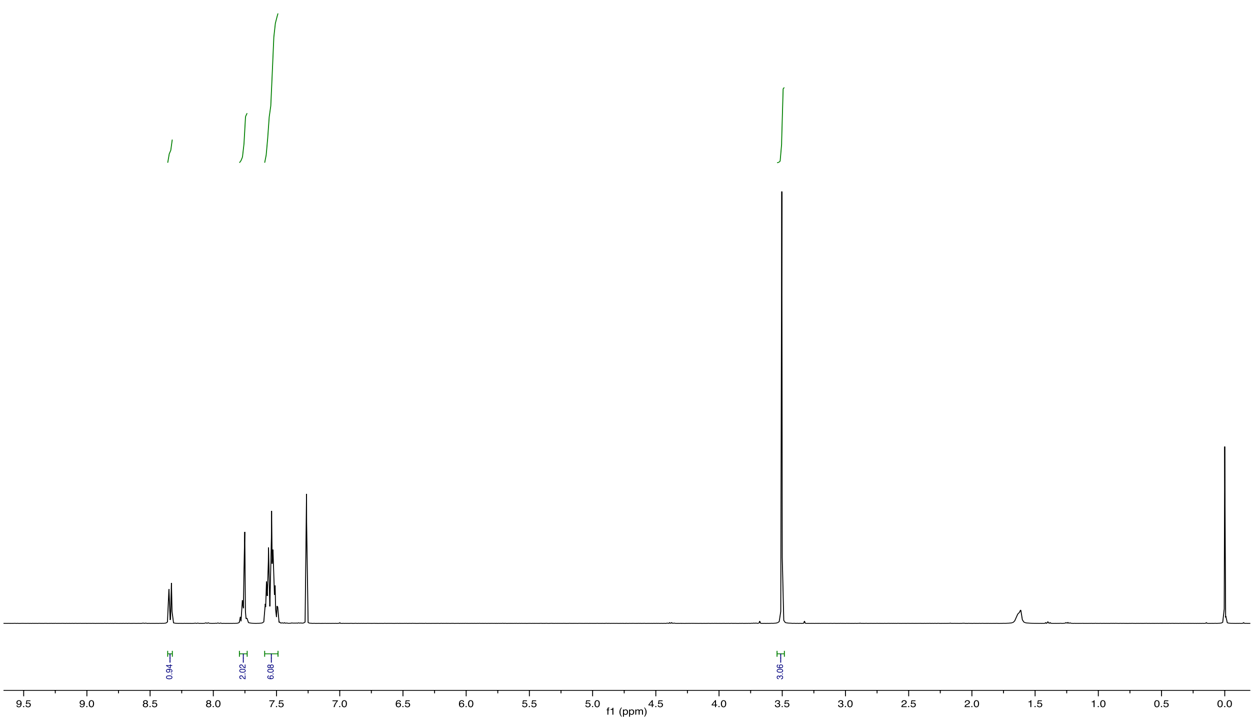

[13]C Spectrum of **16d**

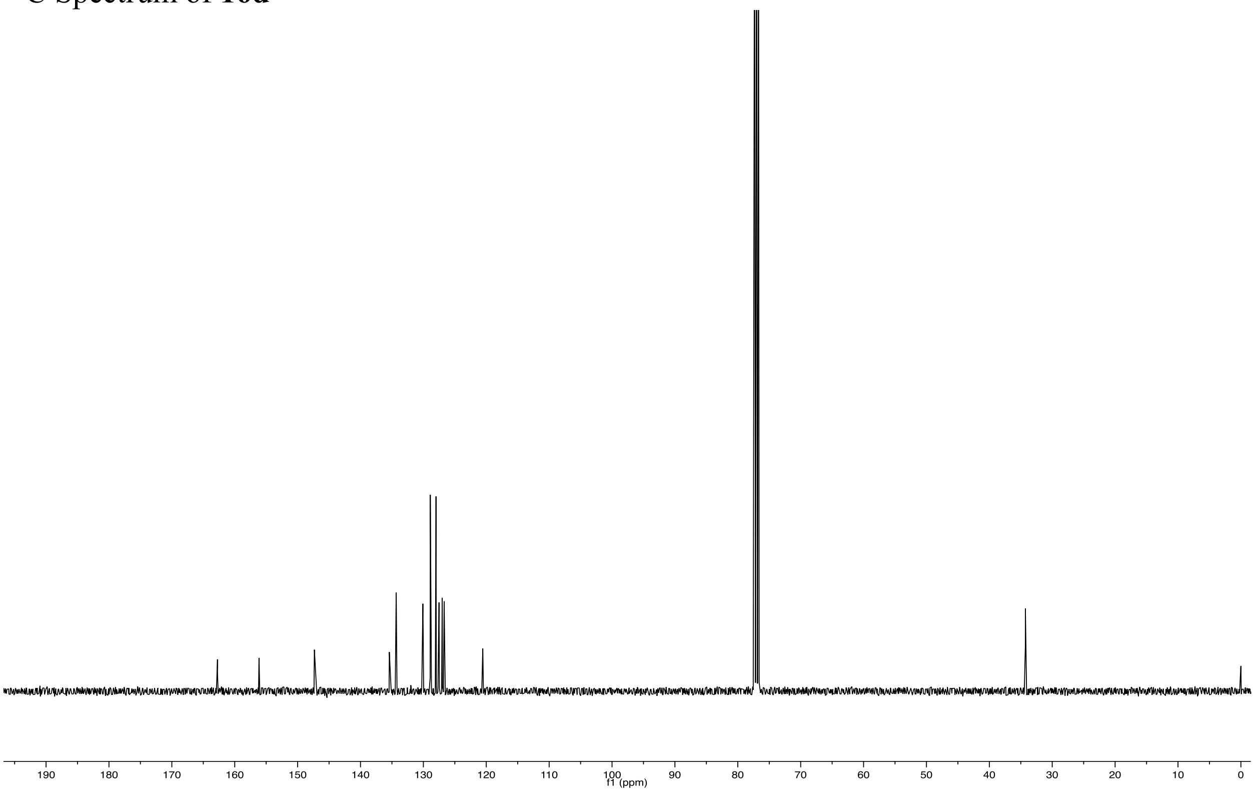

^{1}H Spectrum of 2-Methyl-3-phenylquinazolin-4(3*H*)-one (**18a**)

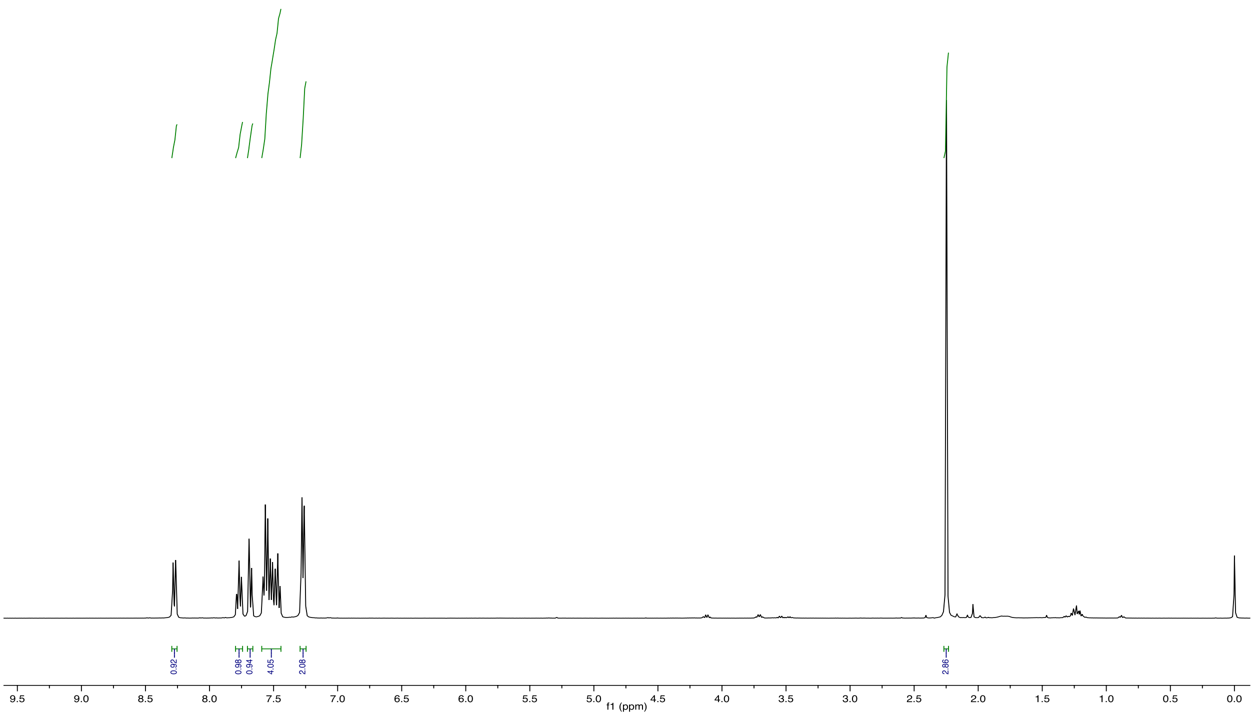

^{13}C Spectrum of **18a**

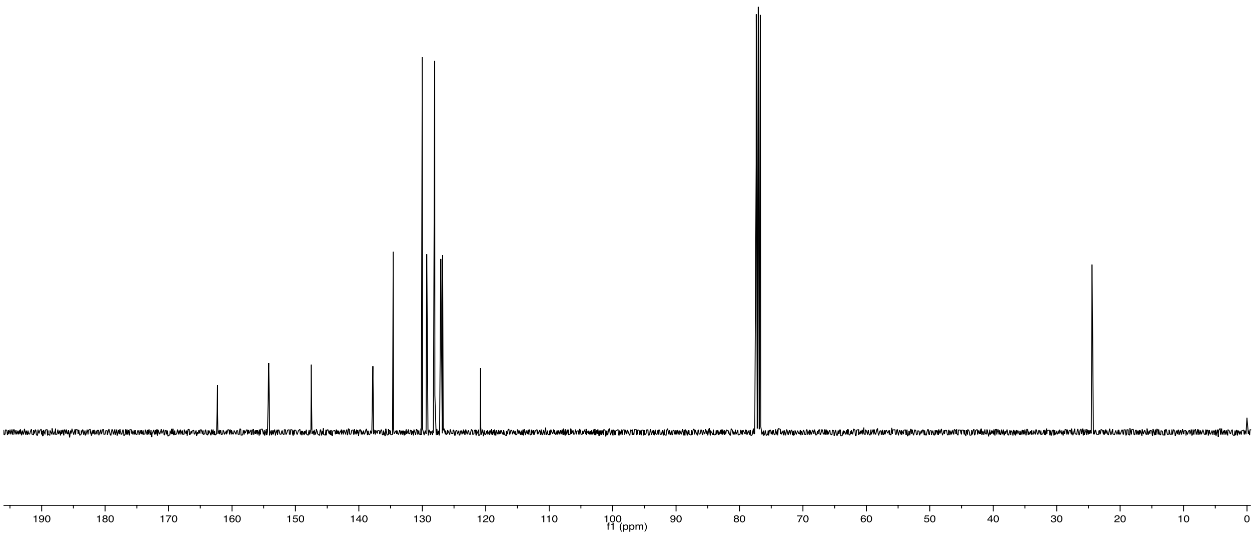

^{1}H Spectrum of 2-Ethyl-3-phenylquinazolin-4(3*H*)-one (**18b**)

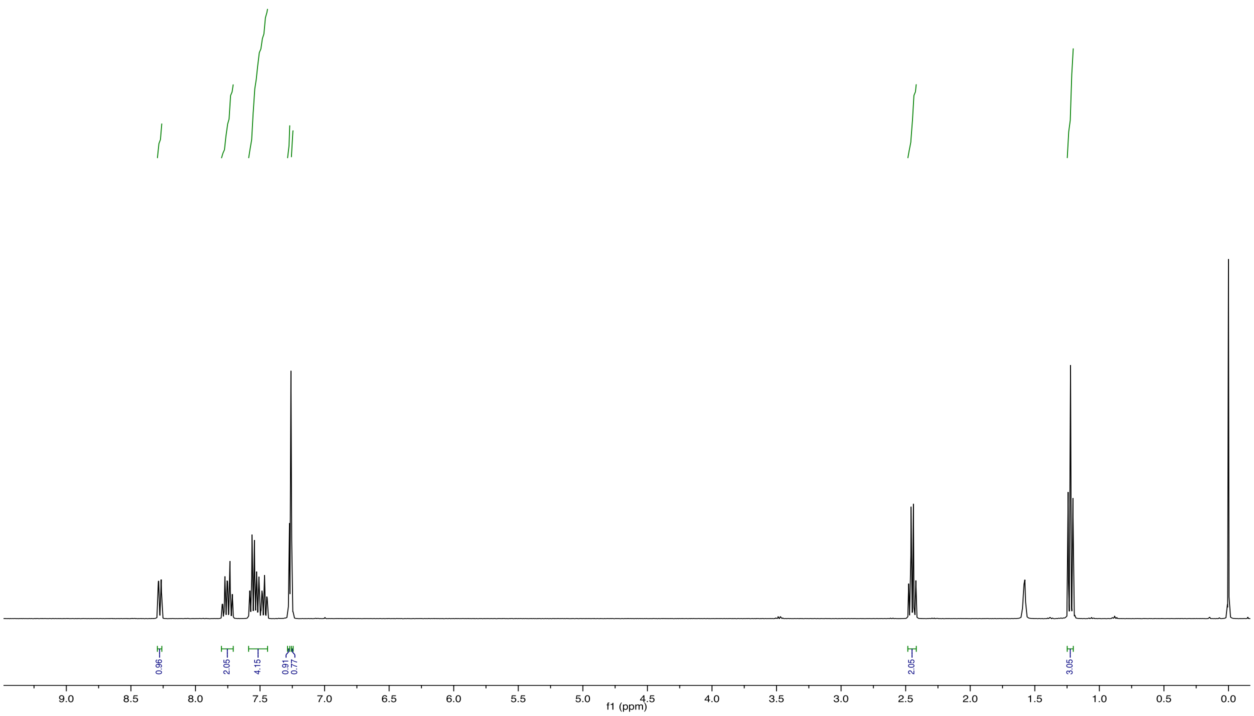

^{13}C Spectrum of **18b**

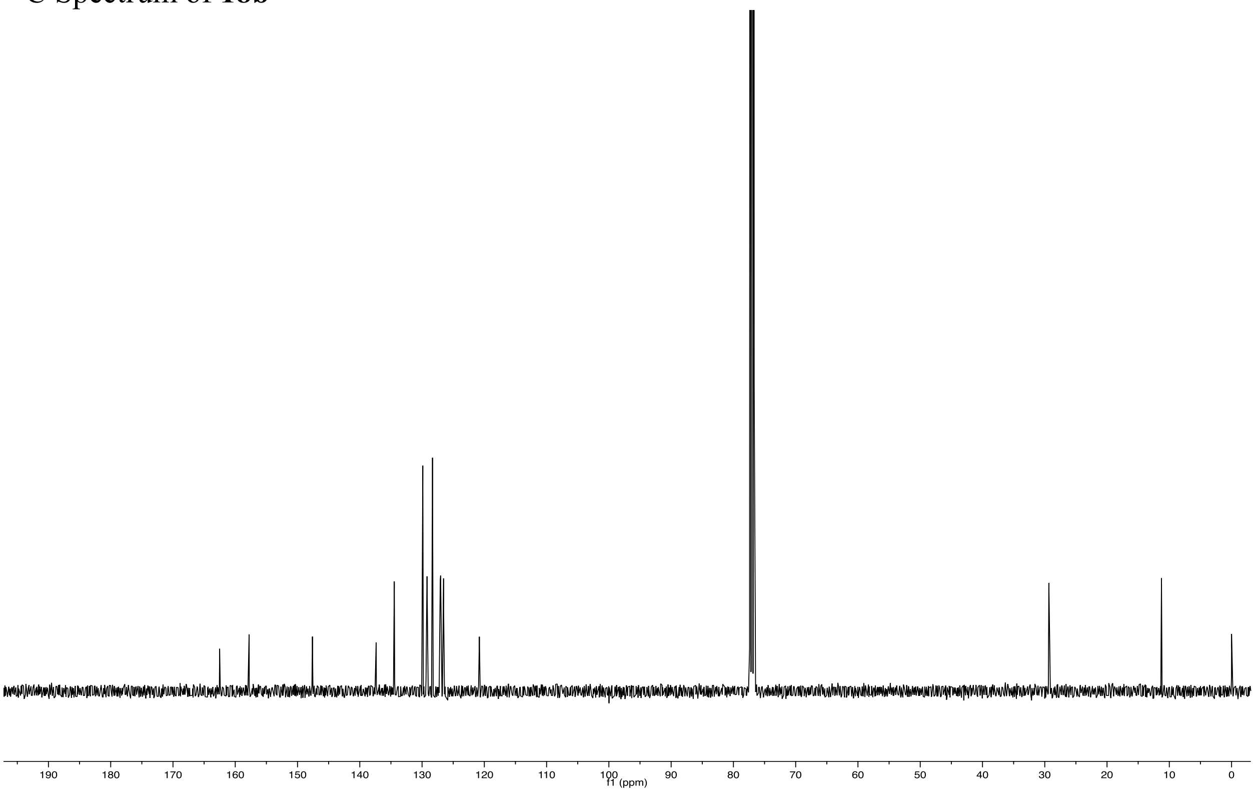

[1]H Spectrum of 2,3-Diphenylquinazolin-4(3*H*)-one (**18d**)

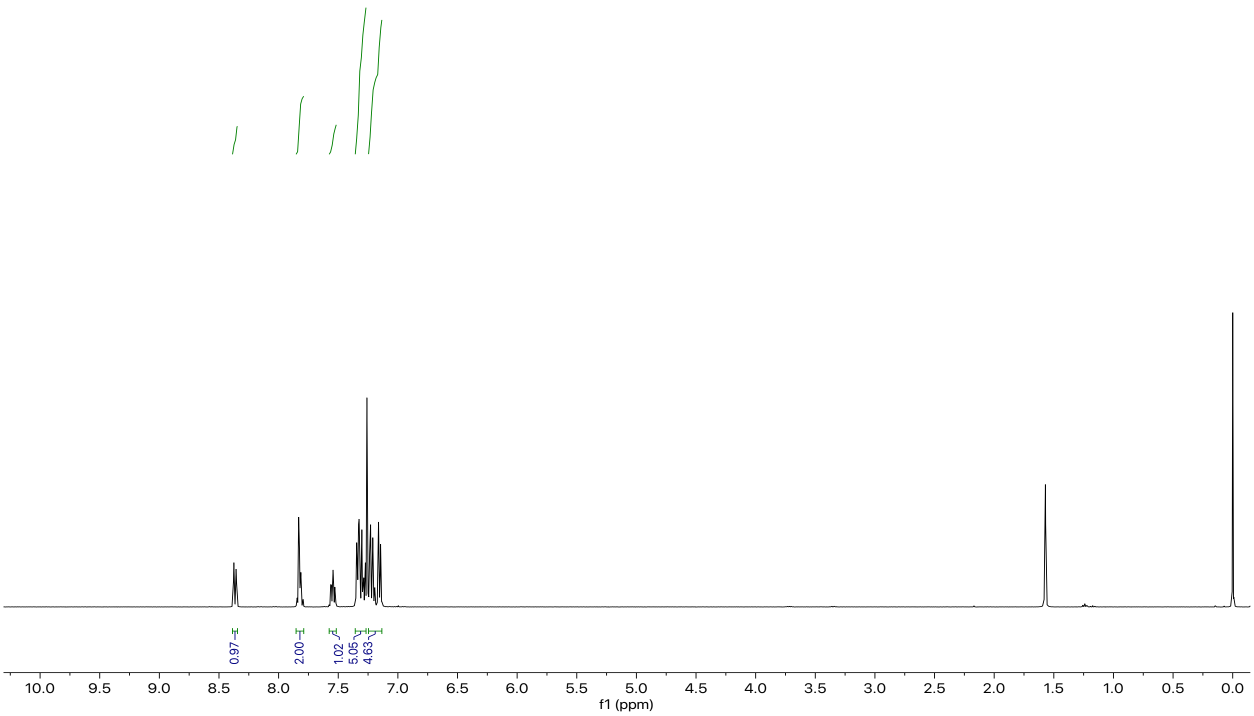

[13]C Spectrum of **18d**

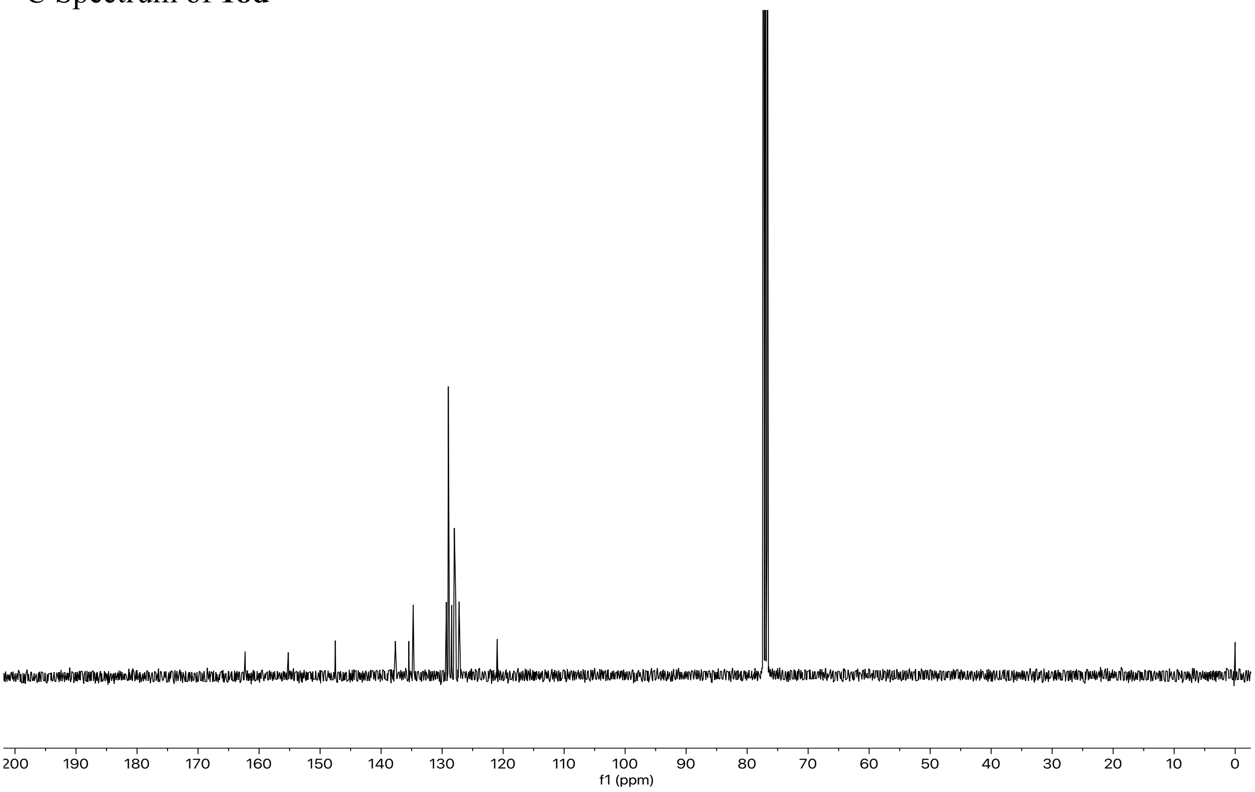

[1]H Spectrum of 3-Amino-2-methylquinazolin-4(3*H*)-one (**20a**)

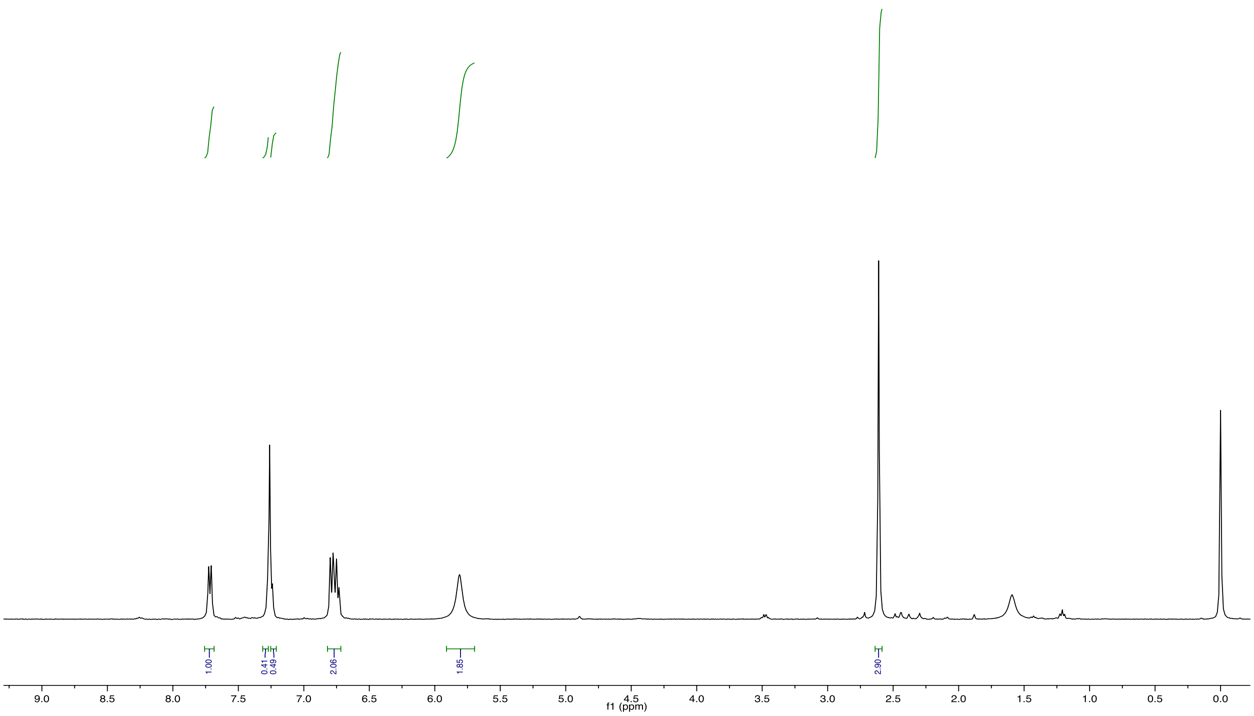

[13]C Spectrum of **20a**

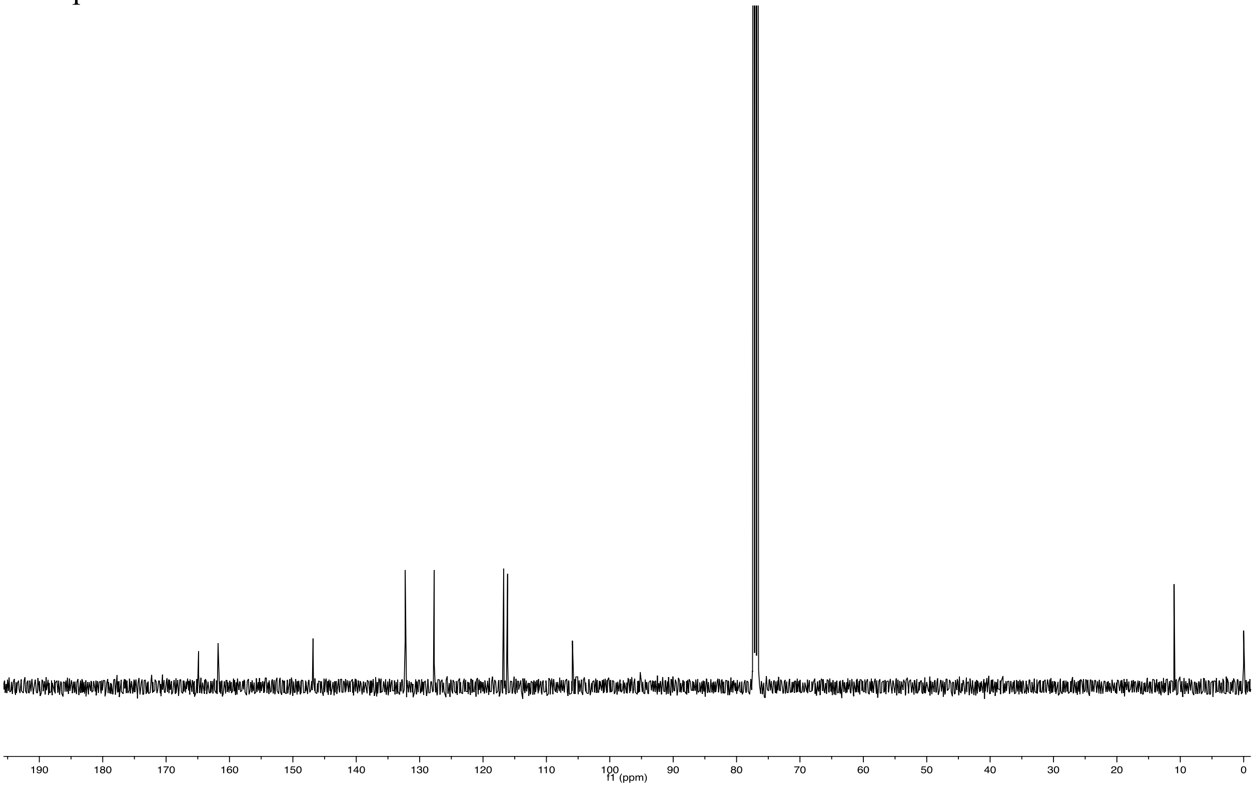

^{1}H Spectrum of 3-Amino-2-ethylquinazolin-4(3*H*)-one (**20b**)

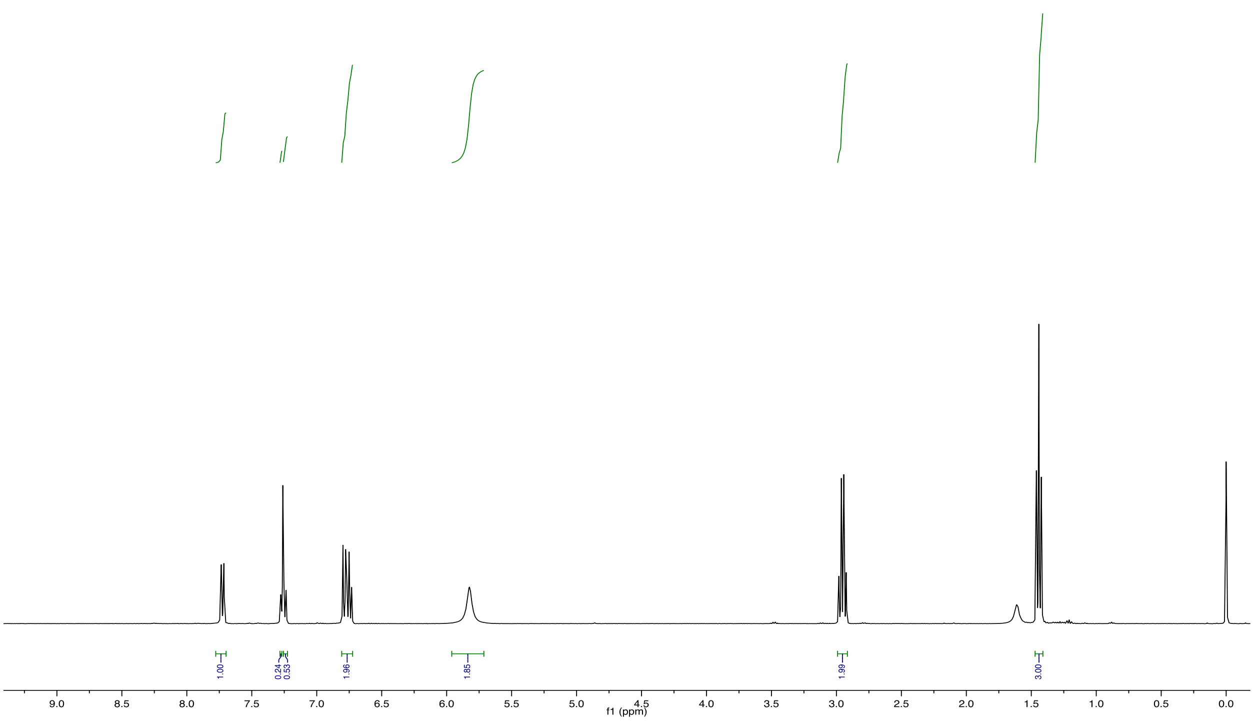

^{13}C Spectrum of **20b**

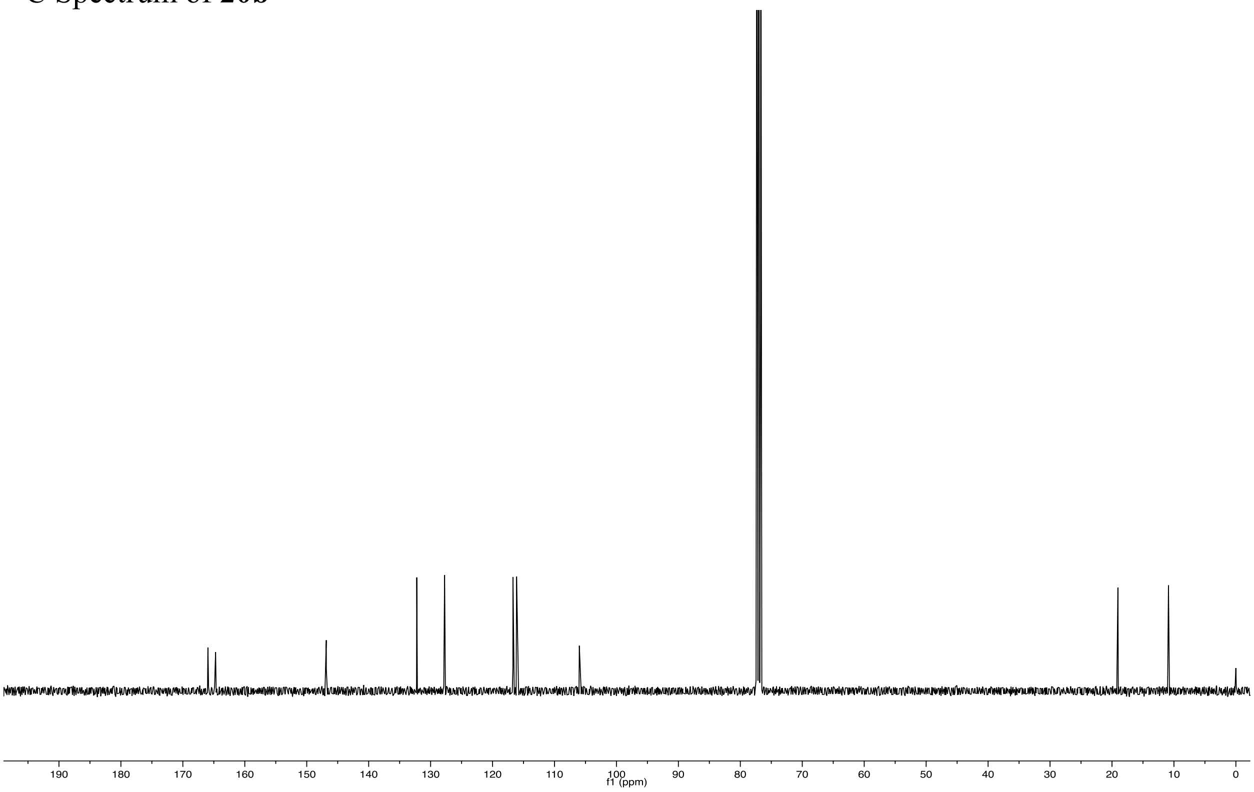

[1]H Spectrum of 3-Amino-2-phenylquinazolin-4(3*H*)-one (**20d**)

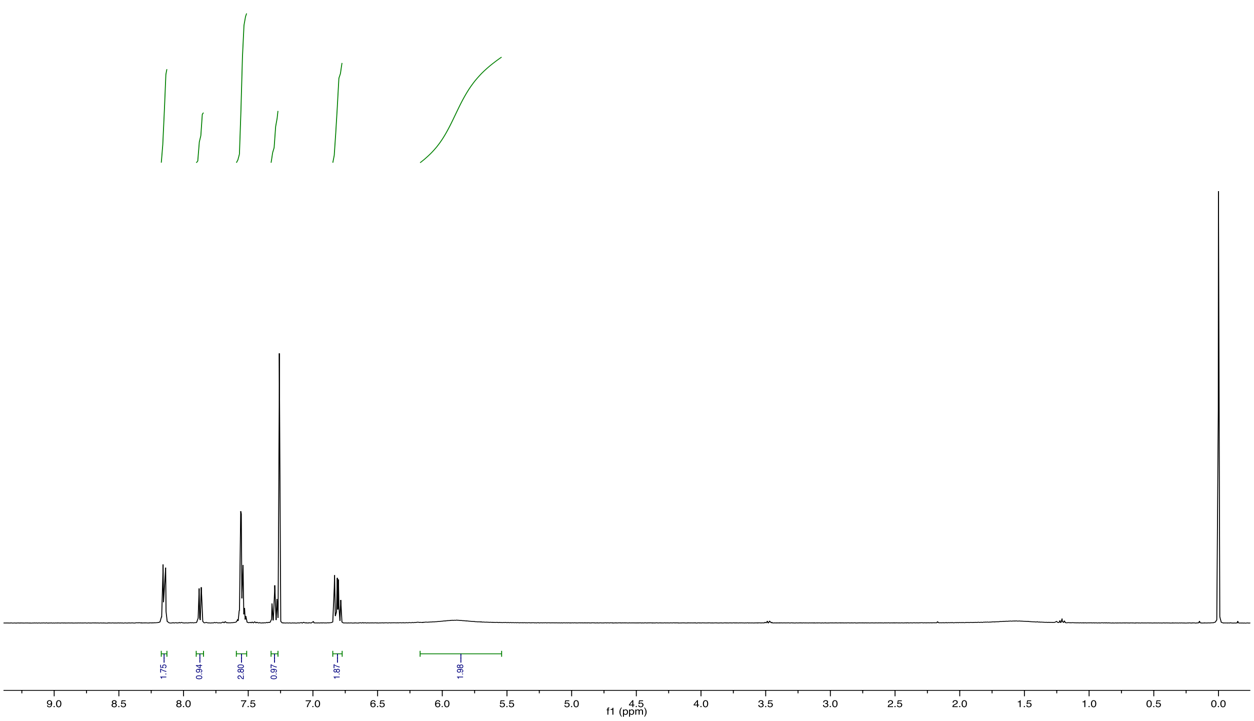

[13]C Spectrum of **20d**

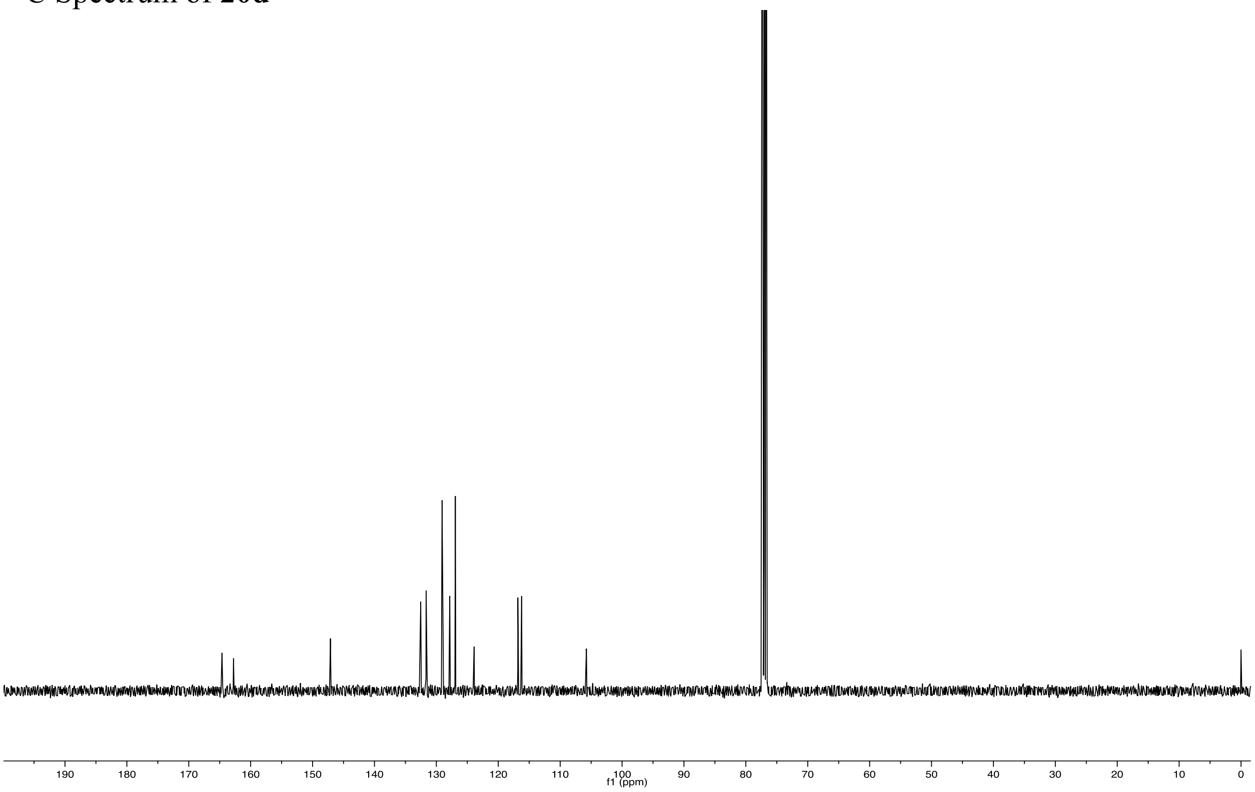

[1]H Spectrum of 2,5,5-Trimethyl-5,6-dihydropyrimidin-4(3*H*)-one (**22a**)

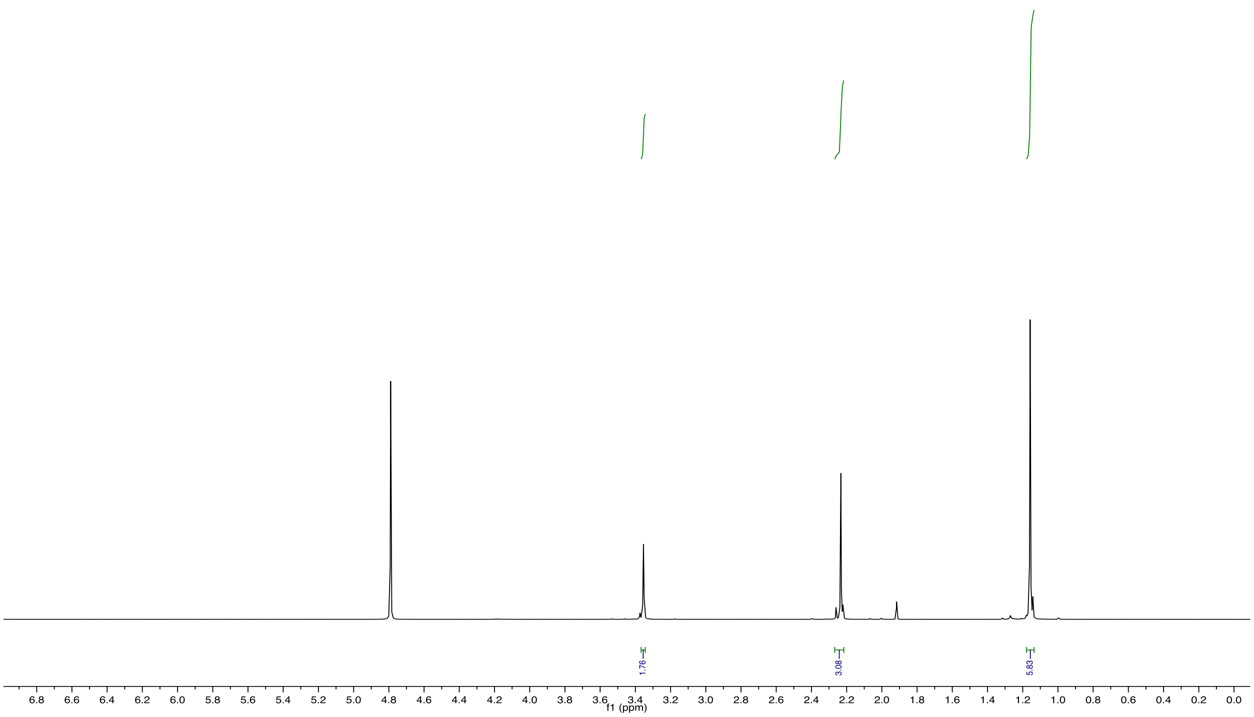

[13]C Spectrum of **22a**

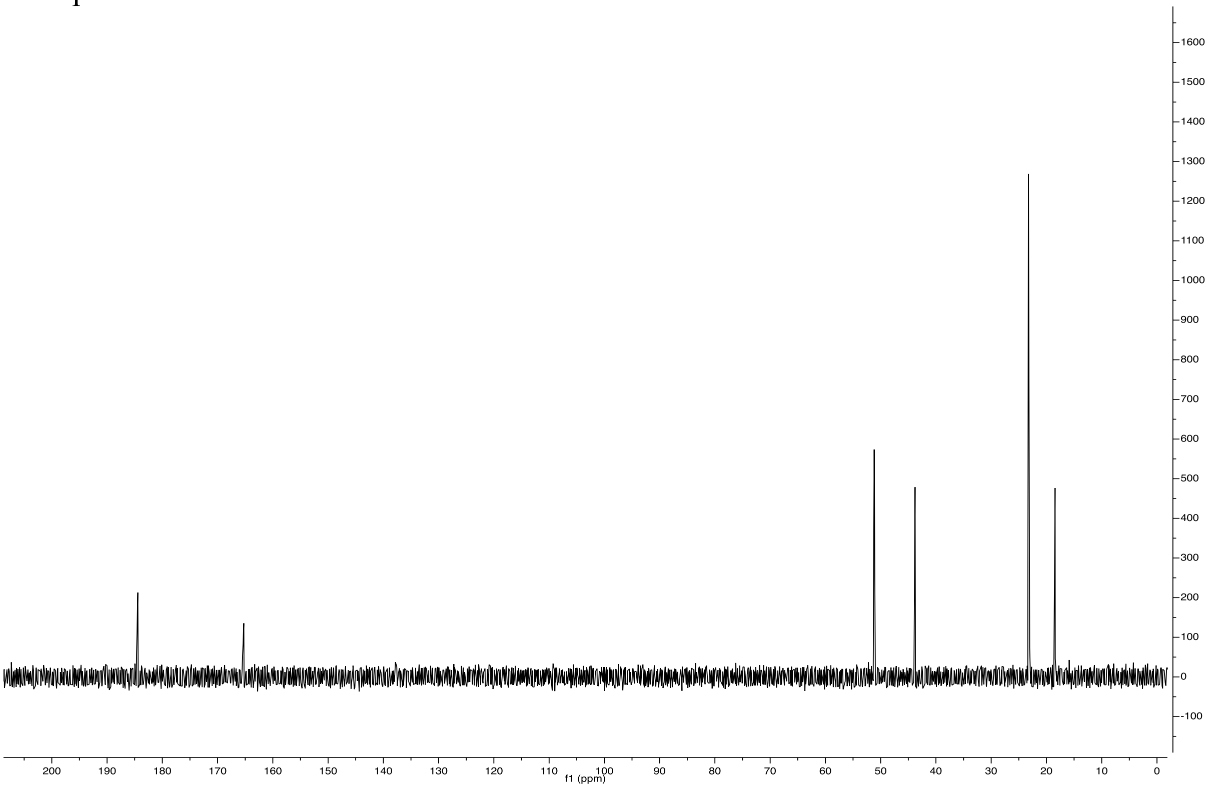

[1]H Spectrum of 2-Ethyl-5,5-dimethyl-5,6-dihydropyrimidin-4(3*H*)-one (**22b**)

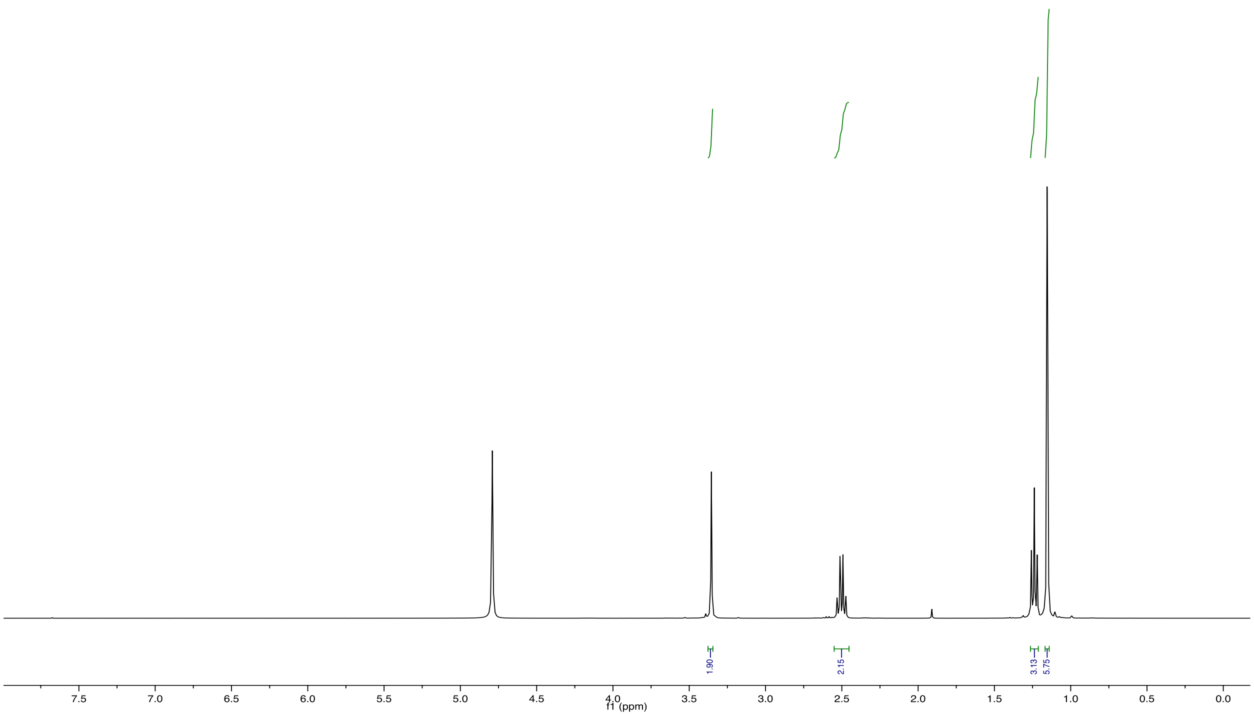

[13]C Spectrum of **22b**

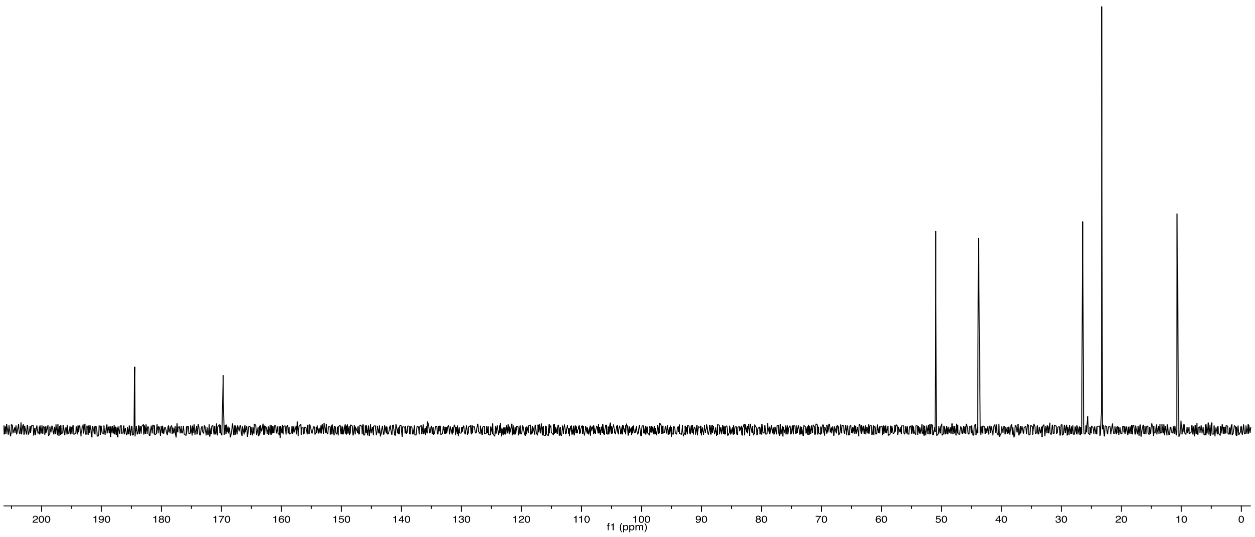

^{1}H Spectrum of 5,5-Dimethyl-2-propyl-5,6-dihydropyrimidin-4(3*H*)-one (**22c**)

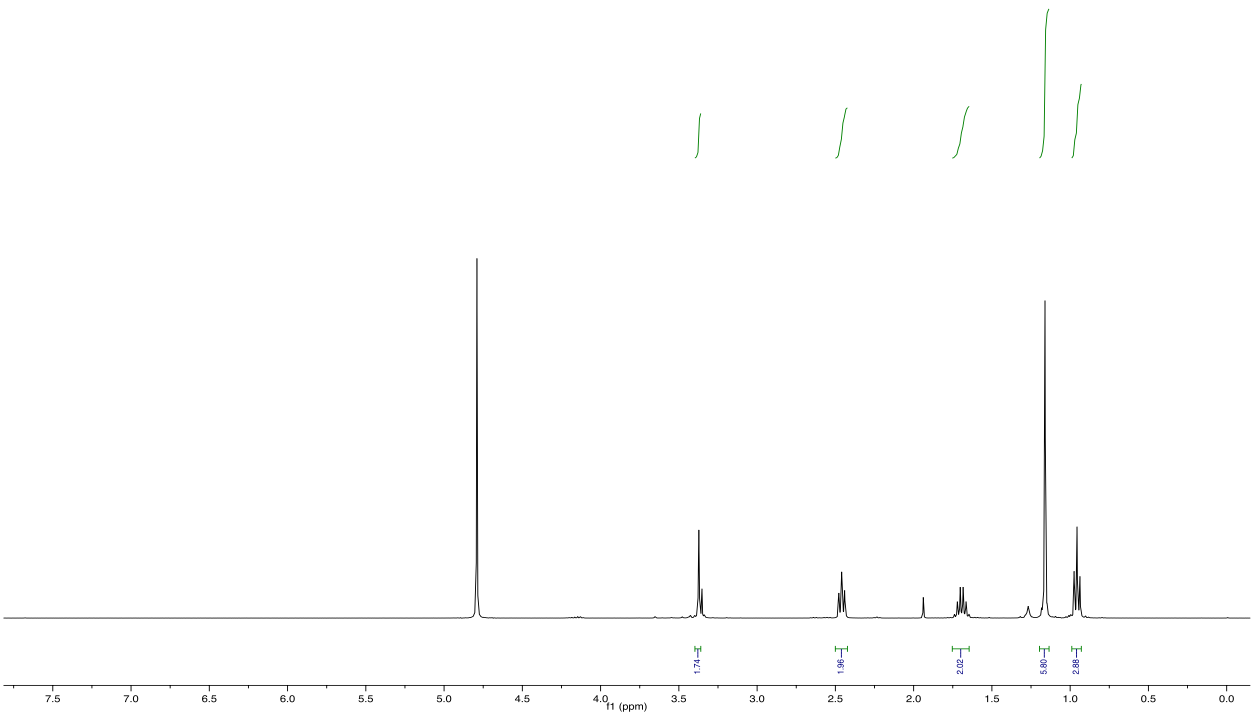

^{13}C Spectrum of **22c**

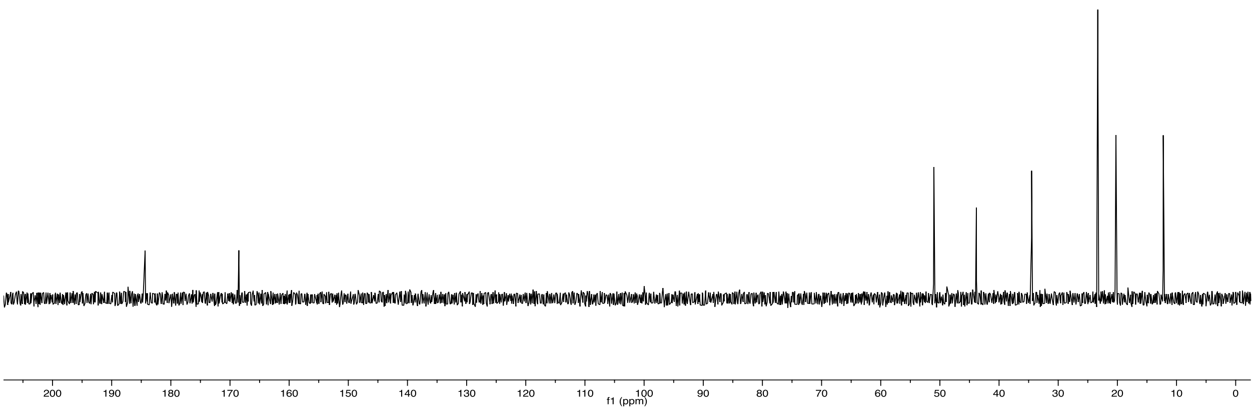

^{1}H Spectrum of 5,5-Dimethyl-2-phenyl-5,6-dihydropyrimidin-4(3*H*)-one (**22d**)

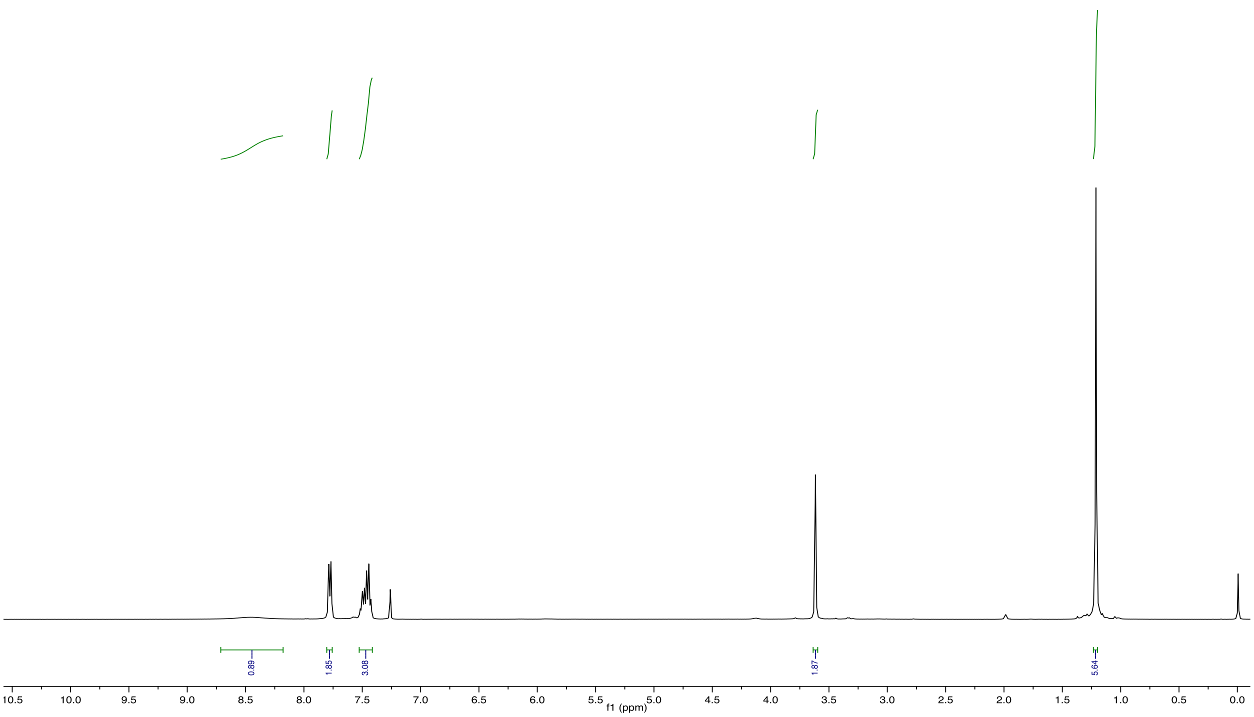

^{13}C Spectrum of **22d**

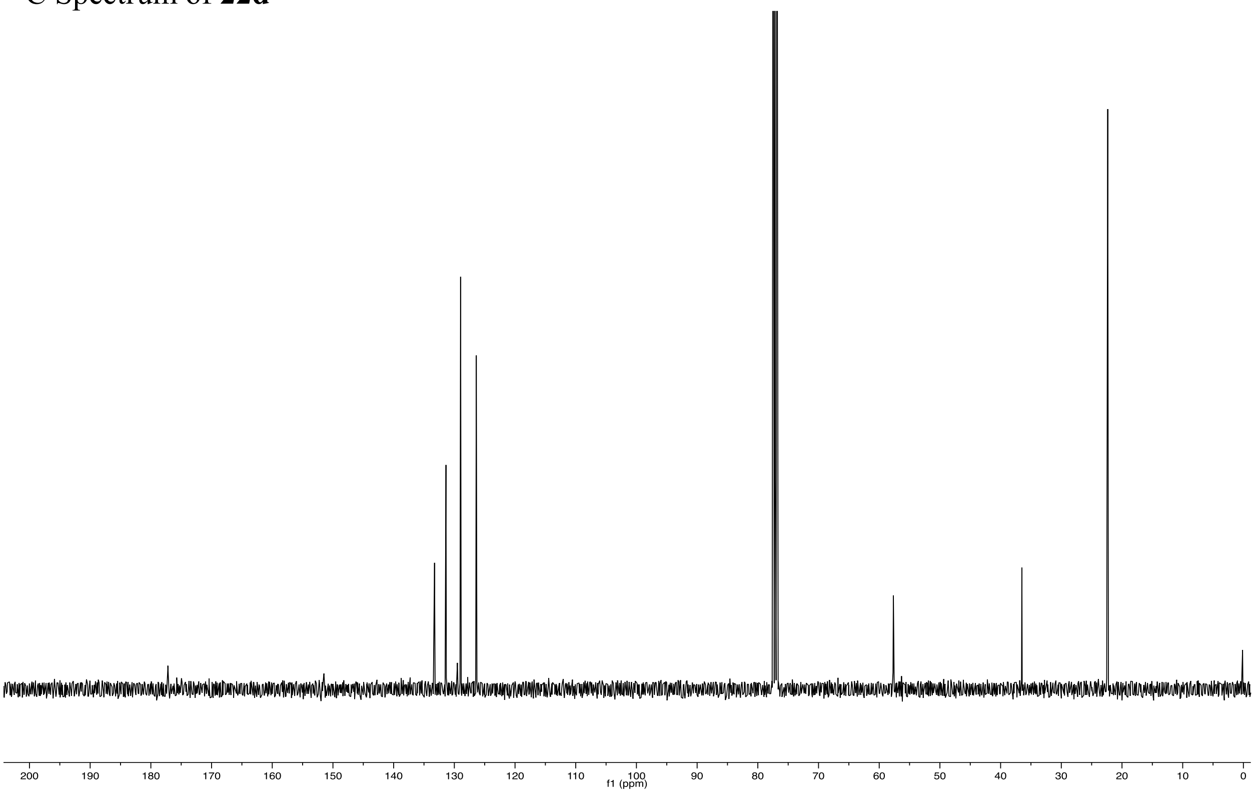

Supplement: Supplementary file 1 [file molecules-23-02925-s001.pdf]
